# Discovery of two novel cutinases from a gut yeast of plastic-eating mealworm for polyester depolymerization

**DOI:** 10.1128/aem.02562-24

**Published:** 2025-04-02

**Authors:** Tong Huang, Jingya Zhang, Xuena Dong, Yu Yang

**Affiliations:** 1 School of Life Science, Beijing Institute of Technology 428678 , Beijing, China; Shanghai Jiao Tong University, Shanghai, China

**Keywords:** plastic waste, mealworm, yeast, *Sakaguchia*, cutinase

## Abstract

Identification of novel plastic-degrading enzymes is crucial for developing enzymatic degradation and recycling strategies for plastic waste. Here, we report the discovery of two novel cutinases, SiCut1 and SiCut2, from a yeast strain *Sakaguchia* sp. BIT-D3 was isolated from the gut of plastic-eating mealworms. Their amino acid sequences share less than 25% identity with all previously described cutinases and reveal a conserved S-D-H catalytic triad with a unique GYSKG motif. Their recombinant proteins were successfully overexpressed in *Pichia pastoris*. The pH range for both enzymes was 4.0 to 11.0 and the temperature range for SiCut1 and SiCut2 was 10°C to 50°C and 10°C to 70°C, respectively. Both enzymes showed strong activity against apple cutin and short-chain fatty acid esters of *p*-nitrophenol and glycerol, substantiating their classification as true cutinases. SiCut1 and SiCut2 have been demonstrated to exhibit efficient degradation of polycaprolactone (PCL) film, polybutylene succinate (PBS) film, and polyester-polyurethane (PUR) foam. Molecular docking and molecular dynamics simulations were used to elucidate the underlying mechanisms of the observed catalytic activity and thermal stability. This study shows that SiCut1 and SiCut2 are novel yeast-derived cutinases with the potential for depolymerization and recycling of plastic waste.

**IMPORTANCE:**

The identification of novel plastic-degrading enzymes is critical in addressing the pervasive problem of plastic pollution. This study presents two unique cutinases, SiCut1 and SiCut2, derived from the yeast *Sakaguchia* sp. BIT-D3 isolated from the gut of plastic-feeding mealworms. Despite sharing less than 25% sequence identity with known cutinases, both enzymes exhibit remarkable degradation capabilities against various polyester plastics, including polycaprolactone (PCL) film, polybutylene succinate (PBS) film, and polyester-polyurethane (PUR) foam. Our results elucidate the catalytic mechanisms of SiCut1 and SiCut2 and provide insights into their potential applications in enzymatic degradation and recycling strategies. By harnessing the gut microbiota of plastic-degrading organisms, this research lays the foundation for innovative enzyme-based solutions to reduce plastic waste and promote sustainable practices in waste management.

## INTRODUCTION

The accumulation of plastic waste poses a significant threat to ecosystems and human health (1). Though well-established, the current approaches to managing plastic trash, such as landfilling and incineration, have come under fire for their long-term unsustainability and secondary pollution (2). Recycling is considered a more promising method that mostly uses mechanical, chemical, and biological processes (3–5). However, mechanical recycling often produces low-value products, which limits their effectiveness (3). Chemical recycling encounters challenges such as harsh reaction conditions and high energy consumption (4). Enzymatic recycling is becoming popular because it can depolymerize plastics into small molecular monomers under moderate conditions (5–7). Furthermore, the depolymerization products can be reused for re-synthesis of new polymers or upcycled into high-quality chemicals (8).

Currently, the most validated enzymes for plastic degradation are polyester-degrading enzymes, with cutinase being the most efficient (9). Cutinase (E.C. 3.1.1.74), which belongs to the α/β-hydrolase superfamily, does not have the hydrophobic lid that covers the active site like serine in true lipases, allowing the active site to accommodate high-molecular-weight substrates, such as natural polyester of plant cutins and synthetic polyesters (10). In general, the validated cutinases with the ability to degrade synthetic polyester can be divided into three categories according to the phylogenetic analysis: the filamentous fungi-derived cutinases, the yeast-derived cutinases, and the bacterial cutinases (10). The representative filamentous fungi-derived cutinases, including FsCut (11–13), AoCut (14), PCLE (15, 16), McCut (17), CgCut (18), FoCut5a (19), HiCut (20), and CpCut1 (21), were identified from the *Fusarium solani pisi*, *Aspergillus oryzae*, *Paraphoma* sp. B47-9, *Malbranchea cinnamomea*, *Colletotrichum kahawae*, *Colletotrichum gloeosporioides*, *Fusarium oxysporum*, *Humicola insolens*, and *Cladosporium* sp. P7, respectively. These filamentous fungi that secrete cutinases were found in a variety of habitats, including plant surfaces, plant residues, soil, fermented foods, and municipal waste compost. The currently known yeast-derived cutinases, including CLE (22, 23), PaE (24, 25), Acut (26), CmCut1 (27), Cr14CLE (28), Plcut1 (29), and BaCut1 (30), had less than 30% sequence identity with filamentous fungi-derived cutinases. They have been identified from yeast strains such as *Cryptococcus* sp. S-2, *Pseudozyma Antarctica*, *Arxula adeninivorans*, *Cryptococcus magnus*, *Cryptococcus nemorosus*, *Papiliotrema laurentii*, and *Blastobotrys* sp. G-9, which were isolated from a range of environments including air, rice husks, plant leaves, plant flowers, soil, fermented foods, aircraft, and landfills. The typical bacterial cutinases, such as TfCut (31, 32), Thc_Cut (33, 34), Tha_Cut (35), and LCC (36), shared less than 20% sequence identity with the filamentous fungi-derived cutinases and the yeast-derived cutinases (37). These bacterial cutinases were mainly detected in a variety of moderately thermophilic actinomycetes, including *Thermobifida fusca*, *Thermobifida alba*, *Thermobifida cellulosilytica*, and *Thermomonospora curvata*. These actinomycetes are aerobic, Gram-positive filamentous bacteria that grow in compost-containing plant debris (37). Overall, the cutinase-producing microorganisms described above, including filamentous fungi, yeasts, and bacteria, are typically isolated from open environments. It is therefore a subject worthy of further study to discover cutinase-secreting microorganisms and polyester-degrading cutinases from other closed or semi-closed bio-environments, such as the animal gut system.

Our previous studies first reported that mealworms (*Tenebrio molitor* larvae) can ingest and degrade polystyrene, commonly known as Styrofoam (38). Subsequent studies have found that mealworms can degrade various polyester plastics, including polylactic acid (PLA), poly (butylene adipate-co-terephthalate) (PBAT), poly (ethylene terephthalate) (PET), polyhydroxyalkanoates (PHA) (39–42). Furthermore, this biodegradation process has been suggested to be driven by the gut microbiota, with several plastic-degrading bacteria being isolated from the guts of mealworms (43–48). The specific polyester-degrading enzymes have been identified in the gut bacteria of mealworms (49). Fungi are also present in the gut microbiota of insects (50), however, the possible role of these fungi in plastic degradation remains to be further explored. Therefore, we wondered whether the specific fungi or enzymes capable of breaking down commercial polyester plastic waste are also present in the gut microbiota of plastic-eating mealworms.

In this study, we isolated a yeast-like fungus *Sakaguchia* sp. BIT-D3 from the gut of plastic-eating mealworms and demonstrated that strain BIT-D3 can secrete enzymes to hydrolyze synthetic polyester. Genomic and transcriptomic analyses identified two genes encoding cutinases in BIT-D3. These two cutinases, SiCut1 and SiCut2, were successfully expressed in *Pichia pastoris*. The catalytic activities of SiCut1 and SiCut2 were characterized using small molecule substrates of *p*-nitrophenol esters and triglycerides, and their ability to degrade natural and synthetic polyesters was tested with apple cutin and various synthetic polyesters. Structural modeling, molecular docking, and molecular dynamics (MD) simulations revealed the mechanisms underlying the catalytic activity and thermal stability. This study demonstrates that SiCut1 and SiCut2 are novel yeast-derived cutinases with the potential for depolymerizing and recycling plastic waste and help elucidate the mechanisms by which insect gut microbiota, particularly fungi, facilitate plastic degradation.

## RESULTS

### Isolation of a polyester-degrading yeast from the gut of plastic-eating mealworm

**Fig 1:**
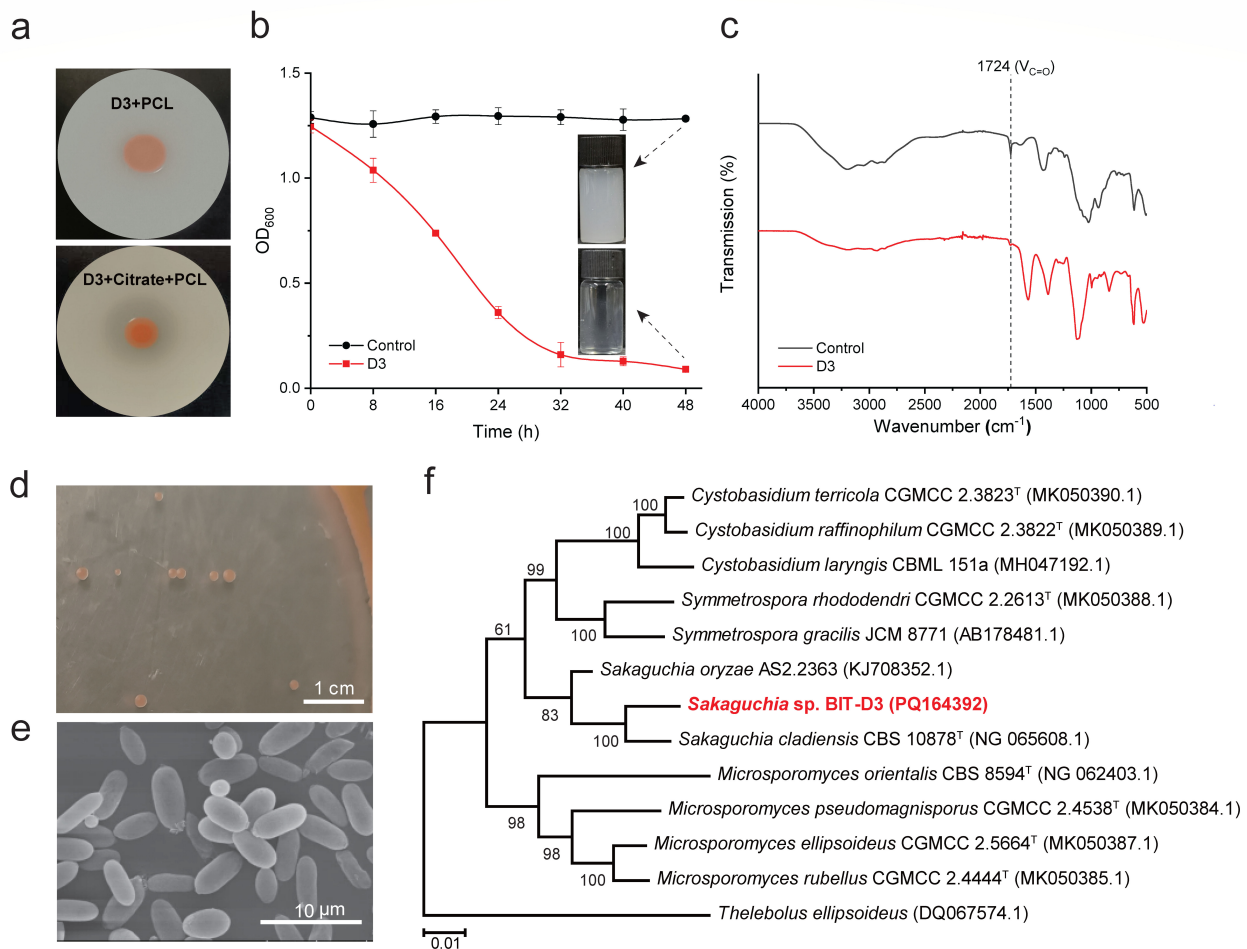
Isolation and characterization of a polyester-degrading yeast strain BIT-D3. (**a**) The clear zone formed by the colony of strain BIT-D3 cultured on YES agar plates containing 0.2% (vol/vol) PCL emulsion with or without citrate supplementation. (**b**) Degradation of PCL emulsion by the extracellular enzymes secreted by strain BIT-D3 in cell-free culture supernatant. The cell-free culture supernatant was harvested from the culture by incubating strain BIT-D3 grown in liquid YES medium with 20 g/L citrate supplementation. Negative controls were subjected to thermal inactivation at 100°C for a period of 2 h before incubation. Error bars represent one standard deviation, *n* ≥ 3. (**c**) Fourier transform infrared (FTIR) spectra of PCL emulsion after treatment with active extracellular enzymes or thermally inactivated controls. (**d**) Photograph of strain BIT-D3 colonies growing on yeast extract peptone dextrose (YPD) agar plate. (**e**) Scanning electron micrograph (SEM) of strain BIT-D3. The cells’ morphology was observed using a scanning electron microscope (SEM, SU8010, Hitachi), and the image was captured using the Hitachi SU-8000 series scanning electron microscope software. It is noteworthy that the presence of straight horizontal lines during the scan of an uneven substrate is a possibility. (**f**) Multilocus sequence analysis (MLSA) based phylogenetic tree of ribosomal RNA genes (18S rRNA, ITS rRNA, and 26S D1/D2 rRNA) of BIT-D3 and its closest related species. Bootstrap values were calculated on the basis of 1,000 replicates. The rRNA sequence numbers are indicated in parentheses.

In this study, five distinct yeasts were isolated and identified from the guts of the plastic-eating mealworms (Table S2). Subsequently, the capability of these yeasts to degrade polyester was determined by observing their ability to form clear zones in the solid assay plates containing polycaprolactone (PCL) emulsion as the substrate. Among the five yeasts, only one, designated strain BIT-D3, was observed to form a clear zone in the solid assay plates when each citrate, pyruvate, or succinate was introduced as an additional carbon source (Fig. 1a; Fig. S2). It was unexpected that the introduction of glucose or lactose as an additional carbon source or with no additional carbon source resulted in the absence of a clear zone. The utilization of citrate, pyruvate, or succinate as an additional carbon source may facilitate the expression of specific enzyme systems, thereby promoting polyester degradation.

We further investigated the PCL polyester degradation ability of the cell-free supernatant of BIT-D3, harvested from yeast extract salts (YES) liquid medium with citrate as a carbon source. Upon exposure to the supernatant, the PCL emulsion underwent significant clarification, which was paralleled by a progressive decrease in optical density (OD_600_) over time (from 1.2 to less than 0.1) (Fig. 1b). After 48 h of incubation, the treated PCL emulsion was almost completely clarified, while the heat-inactivated negative control showed no detectable degradation (Fig. 1b). The residues were collected and lyophilized into dry powders, which were subsequently subjected to Fourier transform infrared spectroscopy (FTIR) analyses. The FTIR spectra revealed a significant decline in the intensity of the carbonyl peak at 1,724 cm^−1^ for the residues obtained from the experimental group in comparison to the control group (Fig. 1c). The results demonstrate that the polyester has been degraded through the cleavage of ester bonds, confirming the capability of strain BIT-D3 to degrade polyester.

Colonies of strain BIT-D3 formed on yeast extract peptone dextrose (YPD) agar exhibited a circular, smooth, convex morphology with an orange-red color and a diameter of 1.0–1.5 mm (Fig. 1d). SEM observations showed that the cells of strain BIT-D3 were composed of short rod-shaped cells (approx 5–7 µm × 3–4 µm) without hyphae or pseudohyphae (Fig. 1e). Strain BIT-D3 showed growth at temperatures ranging from 10°C to 40°C and pH values from 5.0 to 9.0, with optimal growth at 30°C and pH 7.0 (Fig. S3). These characteristics align with the typical morphology of yeast, as previously documented by Casalone et al. (51). In addition, the ITS rDNA sequence analysis showed that strain BIT-D3 is most closely related to the type strain *Sakaguchia dacryoidea* in the genus *Sakaguchia*, with a similarity of 92.11% (Table S2). A multilocus sequence analysis (MLSA) was carried out in order to further clarify the relationship between strain BIT-D3 and its closely related strains, as well as the intra- and inter-genus relationships. An MLSA-based phylogenetic tree was constructed using the concatenated nucleotide sequences of three housekeeping genes, including 18S rRNA, ITS rRNA, and 26S D1/D2 rRNA sequences. As shown in Fig. 1f, the neighbor-joining (NJ) tree indicates that strain BIT-D3 is closely related to *Sakaguchia cladiensis* CBS 10878^T^ in the genus *Sakaguchia*. Based on the results of the physiological characteristics and genetic phylogenetic analyses, strain BIT-D3 can be classified into the genus *Sakaguchia*. We proposed that the polyester-degrading strain BIT-D3 can be named *Sakaguchia* sp. BIT-D3.

### Identification of two potential polyester depolymerases—SiCut1 and SiCut2

Polyester plastic is composed of long-chain macromolecules. The molecular weight of these macromolecules is too high to be directly absorbed into microbial cells; thus, microbial degradation of polyester can only occur as a process outside the cells (1). It can thus be inferred that strain BIT-D3 is capable of secreting the extracellular enzymes necessary for the cleavage of the ester bonds in polyester. To identify the specific enzymes secreted by strain BIT-D3 for polyester degradation, a combination of genomic and transcriptomic analysis was conducted.

**Fig 2:**
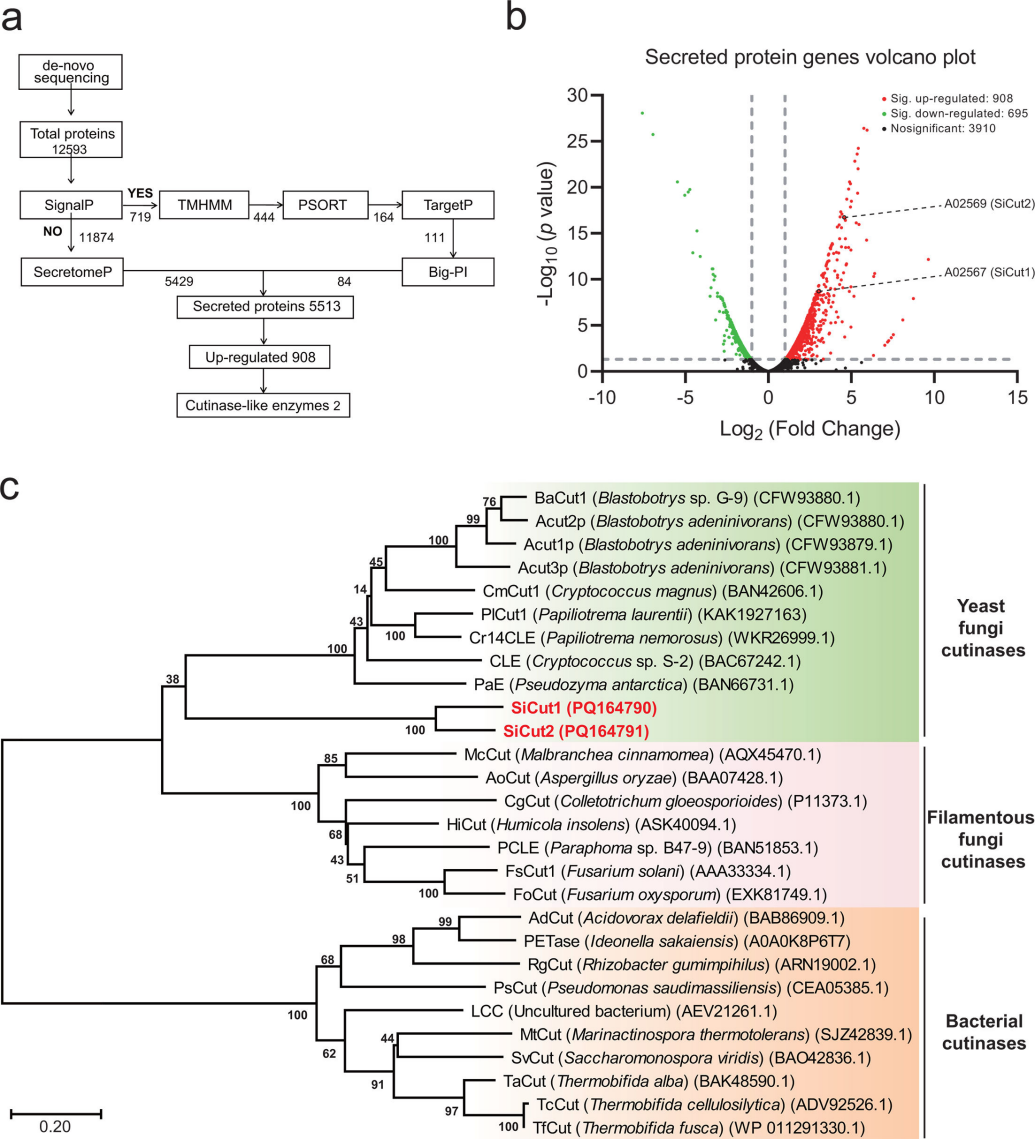
Identification of potential polyester depolymerase from the yeast BIT-D3. (**a**) The procedure for predicting secretory proteins involved in polyester degradation through *in silico* prediction and transcriptomic experiments. Proteins with signal peptides, determined by SignalP-6.0, were identified as secreted proteins by TMHMM Server V2.0, WoLF PSORT, TargetP 2.0 Server, Big-Pi Predictor predicting the presence of transmembrane domains, the subcellular localization of proteins, and GPI anchor points. The other proteins lacking N-terminal signal peptide were screened by SecretomeP 2.0, which was used to predict non-classical secreted proteins. (**b**) Volcano plots showing differences in gene expression of all predicted secretory proteins (5513) in strain BIT-D3 grown in liquid YES medium with PCL emulsion as the substrate in the presence of citrate or glucose as a carbon source. Horizontal coordinates indicate fold changes in gene expression in different samples. Vertical coordinates indicate statistical significance of gene expression differences. Red dots represent significant up-regulated genes with the values of fold change >2 and *P* < 0.05. Green dots represent significant down-regulated genes with the values of fold change <−2 and *P* < 0.05. Black dots indicate no significant changed genes with the values of fold change <2 or >−2 and *P* > 0.05. The spot of two genes *A02567* and *A02569*, encoding the cutinase-like proteins of SiCut1 and SiCut2, was marked out. (**c**) NJ phylogenetic trees constructed with the amino acid sequences of SiCut1 and SiCut2, as well as the previously reported cutinases derived from filamentous fungi, yeast, and bacteria. The sequence numbers of all cutinases are indicated in parentheses. Bootstrap analysis was performed on 1,000 replicates.

The whole genome of strain BIT-D3 was sequenced, resulting in the prediction of a total of 12,593 proteins. In order to annotate the secretory proteins from the total of 12,593 predicted proteins, an *in silico* analysis was conducted using the state-of-the-art protein prediction tools (Fig. 2a). Initially, 12,593 proteins were examined for the presence of a signal peptide using SignalP-6.0 (52). On one hand, 719 proteins of the total 12,593 predicted proteins exhibited N-terminal signal peptides. The 719 proteins were subsequently examined for the presence of transmembrane domains using TMHMM Server V2.0 (53). The results show that 444 proteins of the 719 proteins lacked the transmembrane domains. The subcellular localizations of the 444 proteins were subsequently predicted by using the WoLF PSORT (54) and TargetP 2.0 Server (55). The results reveal that 111 proteins of the 444 proteins were secreted outside the cell membrane. Furthermore, the glycosylphosphatidylinositol (GPI) anchor sites of the aforementioned 111 proteins were determined using the Big-Pi Predictor (56). The results show that 84 proteins of the above 111 proteins lacked the GPI anchor sites. The results of the above analysis indicate that the 84 proteins, which originate from the 719 proteins that contain N-terminal signal peptides, exhibit the characteristics of classical secreted proteins. (Fig. 2a). On the other hand, 11,874 proteins of the total 12,593 predicted proteins lacked N-terminal signal peptides. The non-classical secreted proteins of the 11,874 proteins were examined using SecretomeP 2.0 (57, 58). The results show that a total of 5,429 proteins were predicted to be non-classical secreted proteins (Fig. 2a). In total, 5,513 secreted proteins were annotated in the strain BIT-D3.

Furthermore, transcriptomic analyses were employed to ascertain the secreted enzymes capable of degrading polyesters (Fig. 2b). As the utilization of citrate as an additional carbon source has been demonstrated to promote the facilitation of the expression of specific enzymes capable of degrading polyester by strain BIT-D3, while glucose has not (Fig. 1a; Fig. S2), the strain was grown in liquid YES medium with PCL emulsion as the substrate and citrate as an additional carbon source, and in liquid YES medium with PCL emulsion as the substrate and glucose as an additional carbon source. After a 48 h of incubation period, the transcriptomes of the two groups were sequenced. The results show that 908 secreted proteins of the total 5,513 secreted proteins were significantly up-regulated in the cells cultivated in the presence of citrate in comparison with those cultivated in the presence of glucose (Fig. 2b). The functions of the 908 up-regulated secreted proteins were identified through a BLAST search against the UniProt database (Table S3). Among the 908 up-regulated secreted proteins, two secreted proteins (*A02567*, *A02569*) exhibited the highest amino acid sequence identity (46.9% and 46.2%) with the potential cutinase *V565_000460* of *Rhizoctonia solani* 123E (Uniprot ID: A0A074T1B9). Consequently, these two proteins could be annotated as “cutinase-like enzyme.” Given the known ability of cutinase to cleave ester bonds in polyester, it can be postulated that these two secreted proteins may be involved in the cleavage of ester bonds in polyester (9). Accordingly, these two cutinase-like enzymes will henceforth be designated as SiCut1 (GenBank: PQ164790) and SiCut2 (GenBank: PQ164791) and selected as candidates for protein expression and characterization.

The amino acid sequence identities of SiCut1 and SiCut2 with previously reported cutinases derived from yeast, filamentous fungi, and bacteria were less than 20.7%, 24.2%, and 15.7%, respectively (Table S4). The alignment of the amino acid sequences of SiCut1 and SiCut2 with different yeast-derived cutinases showed the presence of a consensus catalytic triad (S91, D145, H157) with a GYSKG motif (Fig. S4), which is distinct from the GYSQG motif of most cutinases from other yeast (22–30). A phylogenetic tree (Fig. 2c) was constructed by comparing the protein sequences of SiCut1 and SiCut2 with those of the known cutinases capable of degrading a range of polyester plastics. The results showed that SiCut1 and SiCut2 clustered with yeast-derived cutinases, but formed a separate clade within the phylogenetic tree (Fig. 2c). The 3D structures of SiCut1 and SiCut2, as predicted by AlphaFold2, exhibit a high degree of similarity (root-mean-square difference: 1.78 Å or 1.49 Å) to that of CLE (Protein Data Bank code: 2CZQ), which represents the sole experimentally resolved structure for the previously reported yeast-derived cutinase (Fig. S5a). SiCut1 and SiCut2 are primarily composed of five parallel β-sheets and four α-helices, which differs from the structure of CLE, which consists of six parallel β-sheet strands and four α-helices (Fig. S5a). All of the structures exhibit a narrow groove and lack a typical lid structure. In comparison to CLE, SiCut1 and SiCut2 display a more positive electrostatic potential (Fig. S5b) and a comparable hydrophobic interface surrounding the substrate-binding groove (Fig. S5c). These findings indicate that SiCut1 and SiCut2 may represent a novel type of yeast-derived cutinases.

### Expression and biochemical characterization of SiCut1 and SiCut2

**Fig 3:**
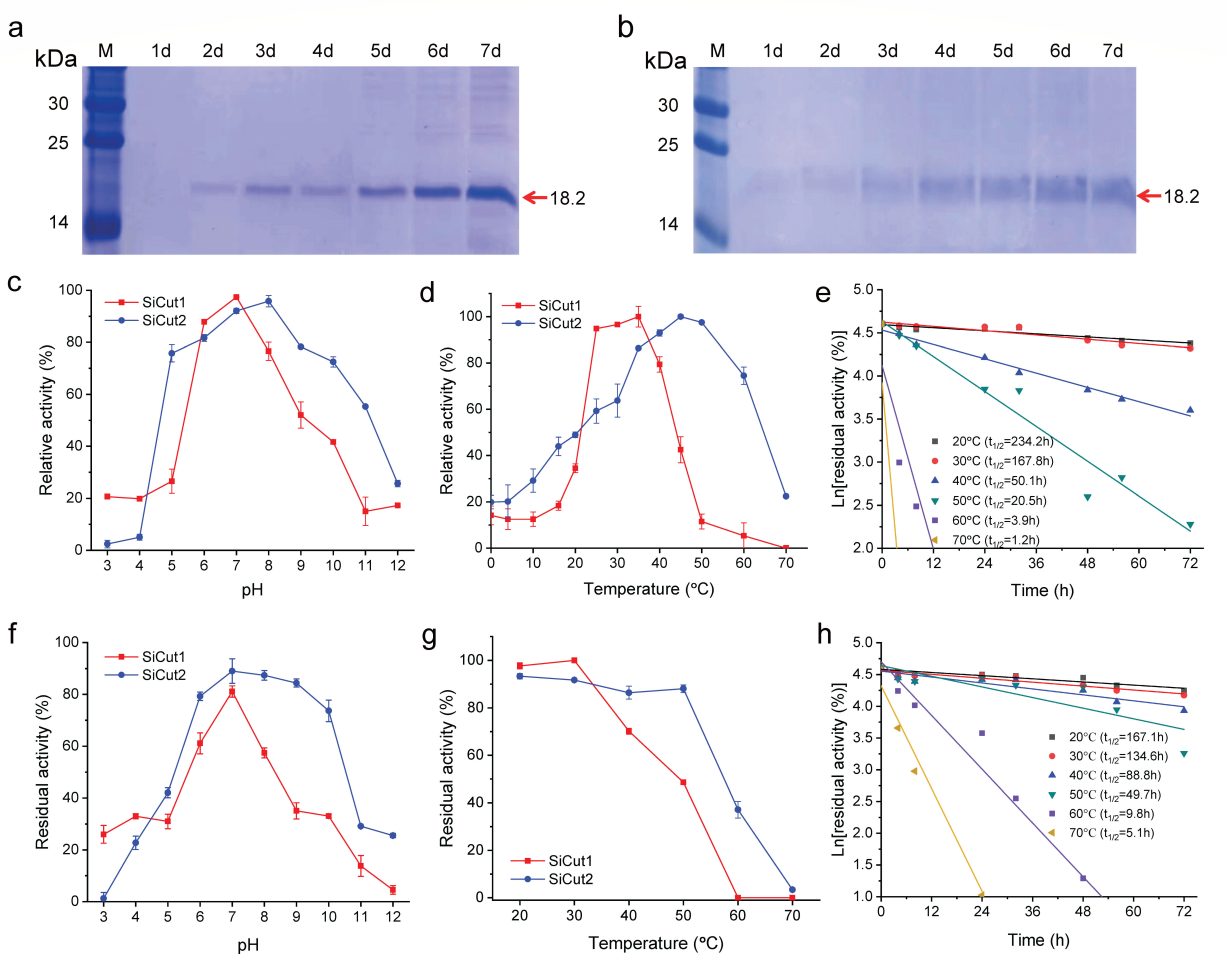
Recombinant expression and biochemical characterization of SiCut1 and SiCut2. (**a**) SDS-PAGE analysis of the crude enzyme of recombinant SiCut1 over 7 days fermentation in *Pichia pastoris*. Lane M: low-molecular-weight standard protein markers; lanes 1 to 7 indicate the expression of SiCut1 at each time point. (**b**) SDS-PAGE analysis of the crude enzyme of recombinant SiCut2 over 7 days fermentation in *Pichia pastoris*. (**c**) Effect of pH on the enzyme activity of SiCut1 and SiCut2. (**d**) Effect of temperature on the enzyme activity of SiCut1 and SiCut2. (**f**) Effect of pH on the enzyme stability of SiCut1 and SiCut2. The assays were conducted using various buffers exhibiting a pH value range from 3.0 to 12.0, including 50 mM sodium citrate buffer (pH: 3.0 to 5.0), 50 mM sodium phosphate (pH: 6.0 to 8.0), 50 mM Tris-HCl (pH: 7.0 to 9.0), and 50 mM glycine-NaOH buffer (pH: 9.0 to 12.0). (**g**) Effect of temperature on the enzyme stability of SiCut1 and SiCut2. (e and h) Half-life curves of SiCut1 (**e**) and SiCut2 (**h**) in different temperatures. The half-life of enzymes was obtained through the relation t_1/2_ = (ln 2)/*k*_d_.

The two cutinase-like enzymes, SiCut1 and SiCut2, were overexpressed in *P. pastoris* GS115 under the control of the *AOX1* promoter. SDS-PAGE analysis revealed the presence of a predominant 18.2 kDa protein in cell culture supernatants, with minimal secretion of other proteins into the medium (Fig. 3a and b). During a 7-day incubation period, the protein concentration of SiCut1 and SiCut2 increased gradually and reached a maximum point of 160 ± 2 µg/mL and 108 ± 7 µg/mL, respectively (Fig. S6). Using *p*NP-butyrate (*p*NPB) as a substrate, the enzyme activities of the recombinant proteins of SiCut1 and SiCut2 exhibited a gradual increase and reached a maximum point of 0.41 ± 0.03 U/mL and 0.37 ± 0.02 U/mL, respectively.

The optimal pH for SiCut1 and SiCut2 was found to be 7.0 and 8.0, respectively (Fig. 3c), while the highest stability for SiCut1 and SiCut2 was both observed at pH 7.0 (Fig. 3f). This result indicates that the two enzymes are more active under neutral conditions. The optimal temperature for SiCut1 was observed at 35°C, while that for SiCut2 was 45°C (Fig. 3d). At temperatures below 30°C, SiCut1 showed superior thermal stability compared to SiCut2. However, at temperatures above 30°C, SiCut2 exhibited enhanced thermal stability compared to SiCut1 (Fig. 3g). The half-life of SiCut1 was longer than that of SiCut2 when the temperature was below 30°C, but it was significantly shorter than that of SiCut2 when the temperature was above 30°C (Fig. 3e; Fig. 3h). This indicates that SiCut2 is markedly more stable than SiCut1 at elevated temperatures above 30°C.

### Hydrolytic activity of SiCut1 and SiCut2 against *p*-nitrophenol esters, triglycerides, and cutin

The lipolytic and esterolytic activities of SiCut1 and SiCut2 were examined using a series of *p*-nitrophenol esters and triglycerides with varying acyl chain lengths as substrates. The highest activity for SiCut1 and SiCut2 was observed with *p*NP-hexanoate (C6) and *p*NP-octanoate (C8), respectively (Table 1; Fig. S7). With regard to triglyceride substrates, both SiCut1 and SiCut2 exhibited comparable activity profiles, with increased activities observed for the short-chain substrates compared to the long-chain substrates (Table 2). These properties are consistent with the common feature of cutinases (26).

**TABLE 1:** Hydrolysis of *p*-nitrophenyl (*p*NP) esters by SiCut1 and SiCut2

*p*NP ester	SiCut1			SiCut2		
	*K*_cat_ (min^−1^)	*K*_m_ (µM)	*K*_cat_/*K*_m_ (min^−1^ µM^−1^)	*K*_cat_ (min^−1^)	*K*_m_ (µM)	*K*_cat_/*K*_m_ (min^−1^ µM^−1^)
*p*NP-acetate (2C)	19.27 ± 0.16	9.12 ± 0.04	2.11 ± 0.18	39.89 ± 4.2	6.18 ± 0.49	6.46 ± 0.68
*p*NP-butyrate (4C)	47.76 ± 0.22	1.73 ± 0.99	27.59 ± 0.14	68.01 ± 3.96	2.82 ± 0.58	24.09 ± 0.3
*p*NP-hexanoate (6C)	51.53 ± 0.43	0.66 ± 2.77	78.32 ± 0.11	75.36 ± 1.05	1.44 ± 1.51	52.52 ± 0.7
*p*NP-octanoate (8C)	60.14 ± 0.16	2.01 ± 0.63	30.06 ± 0.09	124.71 ± 0.15	0.89 ± 0.12	140.66 ± 0.83
*p*NP-decanoate (10C)	40.31 ± 1.56	7.57 ± 0.01	5.33 ± 0.19	88.91 ± 2.05	2.47 ± 0.09	36.04 ± 0.04
*p*NP-laurate (12C)	43.40 ± 0.17	3.21 ± 0.63	13.50 ± 0.03	58.95 ± 2.1	2.22 ± 1.9	26.58 ± 0.26
*p*NP-myristate (14C)	12.81 ± 0.13	3.46 ± 0.02	3.70 ± 0.08	49.92 ± 0.7	4.44 ± 0.5	11.25 ± 0.6
*p*NP-palmitate (16C)	12.93 ± 0.12	7.09 ± 0.27	1.82 ± 0.13	36.19 ± 2.5	4.01 ± 1.3	9.03 ± 0.23

**TABLE 2:** Hydrolysis of triglycerides by SiCut1 and SiCut2

Triglyceride	Value (U/mg)	
	SiCut1	SiCut2
Triacetin (2C)	1.75 ± 0.42	1.66 ± 0.26
Tributyrin (4C)	1.6 ± 0.21	1.53 ± 0.15
Tricaproin (6C)	1.5 ± 0.07	1.47 ± 0.06
Tricaprylin (8C)	1.04 ± 0.14	1.43 ± 0.1
Tricaprin (10C)	0.31 ± 0.05	0.8 ± 0.13
Trilaurin (12C)	0 ± 0	0.34 ± 0.02

**Fig 4:**
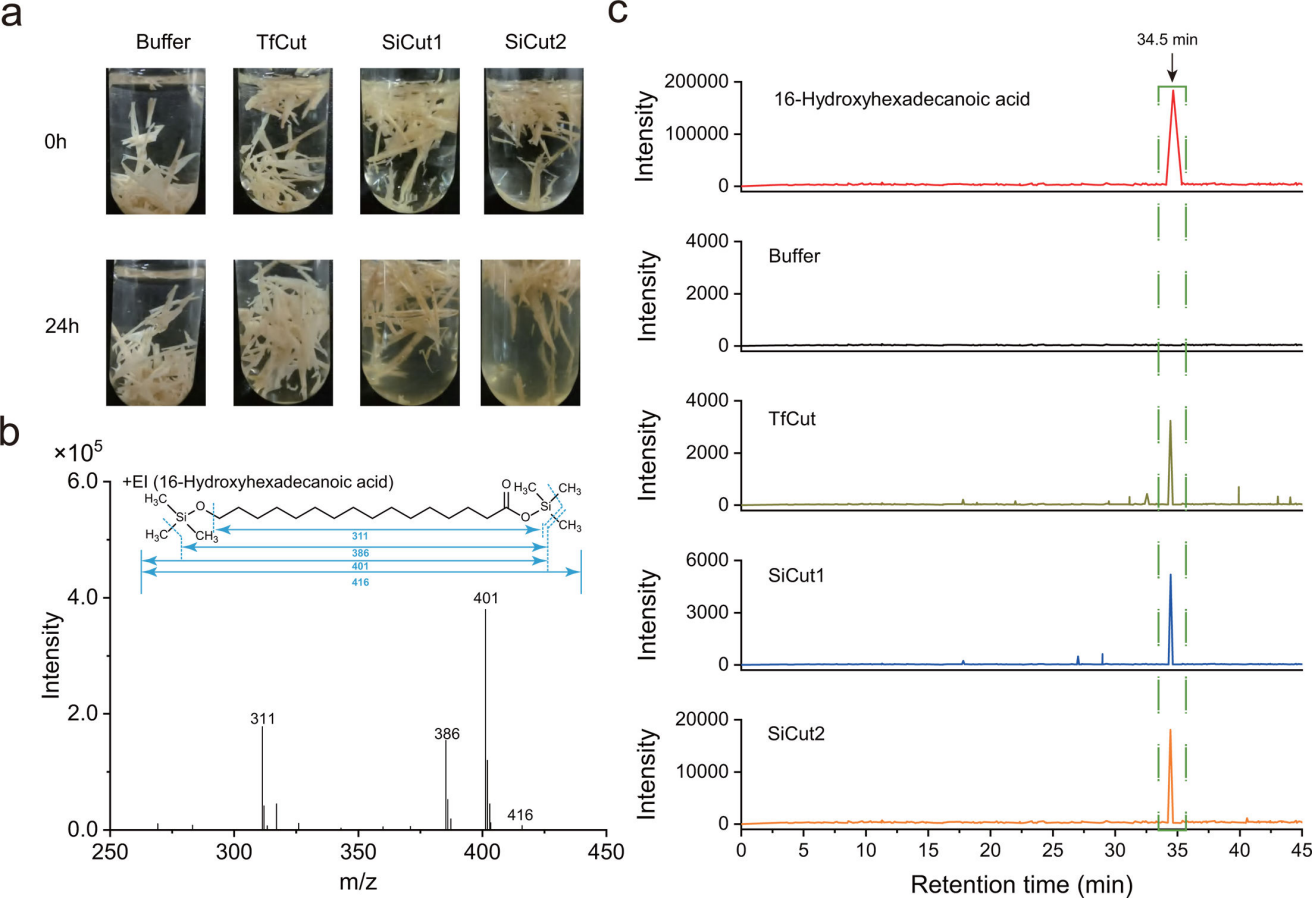
Degradation of cutin by SiCut1 and SiCut2. (**a**) Photographs of tubes containing apple cutin treated with each of purified SiCut1, SiCut2, TfCut (positive control), or buffer control (no enzyme) at the optimal temperature (35°C for SiCut1, 45°C for SiCut2, 55°C for TfCut) after 24 h. The crude cutin was separated from Red Fuji apple peels. (**b**) Standard mass spectrum (MS) of the derivative of 16-hydroxyhexadecanoic acid. The m/z of 401 is a steady-state product of the derivative of 16-hydroxyhexadecanoic acid (m/z = 416). (**c**) GC-MS analyses of degradation products of cutin by SiCut1, SiCut2, and TfCut. The EICs were obtained using a mass window of m/z 401, corresponding to a typical cutin monomer (16-hydroxyhexadecanoic acid, C_16_H_32_O_3_). The standard 16-hydroxyhexadecanoic acid and all samples of hydrolysis products were silylated with N-methyl-N-(trimethylsilyl) trifluoroacetamide (MSTFA) before GC-MS analysis.

The defining characteristic of cutinases is their ability to degrade cutin into a mixture of long alkyl chain alcohols and carboxylic acids, with 16-hydroxyhexadecanoic acid representing the predominant monomer product (32). Therefore, we tested their ability to degrade apple cutin and analyzed the degradation products via gas chromatography-mass spectrometry (GC-MS) (Fig. 4a). The extracted ion chromatogram (EIC) was employed to demonstrate the presence of the target products of 16-hydroxyhexadecanoic acid in the samples, based on the intensity of a selective mass-to-charge ratio (m/z) of 401 that corresponds to a steady-state product of 16-hydroxyhexadecanoic acid in the standard mass spectra (Fig. 4b). As illustrated in Fig. 4c, the EIC of the standard 16-hydroxyhexadecanoic acid exhibited a pronounced peak at a retention time of 34.5 min. As a positive control, the EIC of the degradation products released by the previously documented bacterial cutinase TfCut also exhibited a notable peak at 34.5 min, which aligns with the findings previously reported by Chen et al. (32). Moreover, the EIC of the SiCut1- or SiCut2-treated sample also exhibited an evident peak at 34.5 min. In contrast, the EIC of the negative control (buffer-treated sample), which had not undergone enzymatic treatment, did not display a peak at 34.5 min. In summary, we proposed that SiCut1 and SiCut2 can be classified as members of the cutinase family.

### Hydrolytic activity of SiCut1 and SiCut2 against various polyester plastics

**Fig 5:**
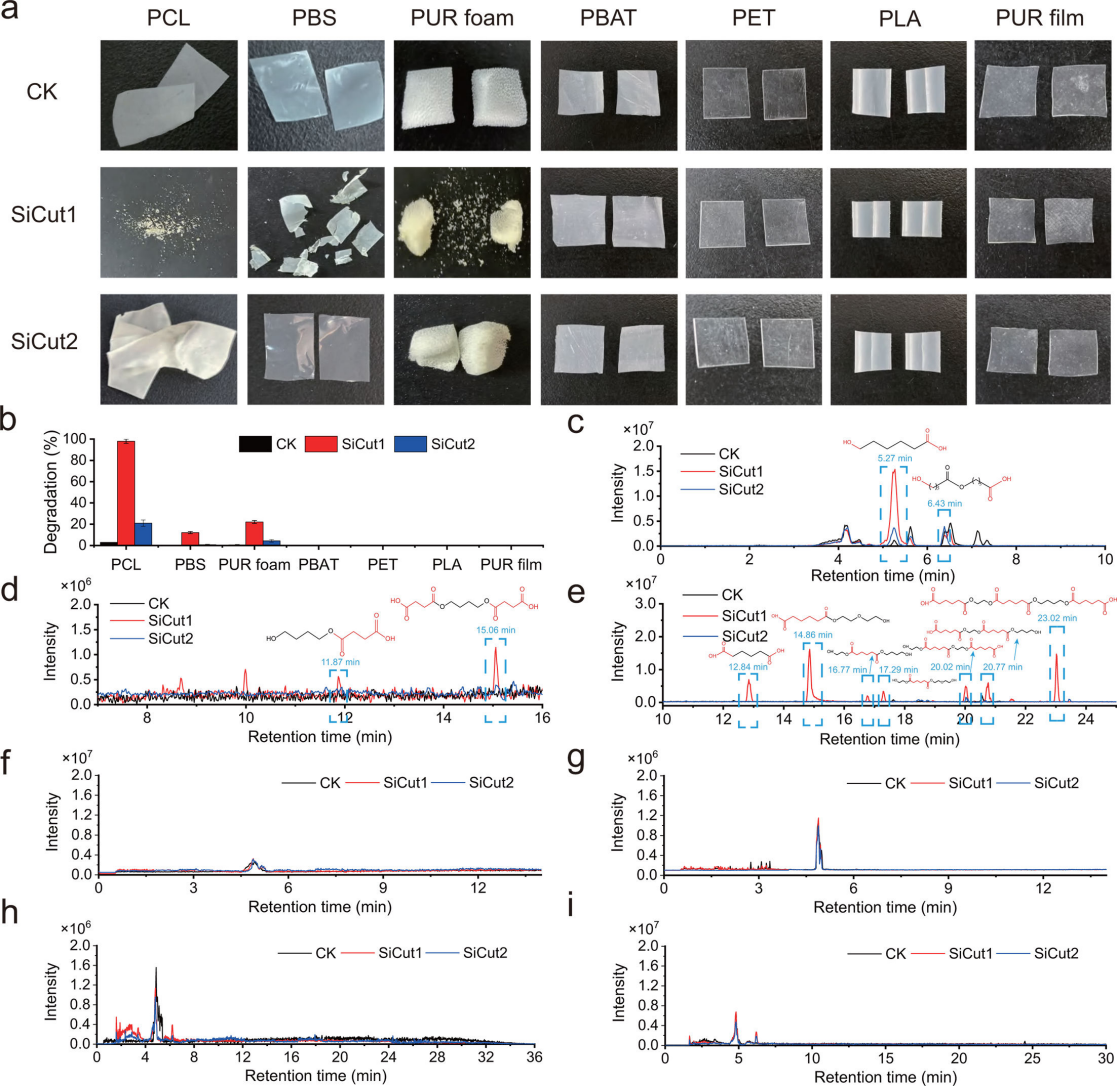
Hydrolytic activity of SiCut1 and SiCut2 against various polyester plastics. (**a**) Photographs of PCL films, PBS films, polyester-PUR foams, PBAT films, PET films, PLA films, and polyester-PUR films degraded by SiCut1 and SiCut2 for 48 h. The reaction mixture contained 100 µL of 100 µM enzyme solution, 900 µL of Tris-HCl buffer (50 mM, pH 7.5), and 10 mg of each film (2.0 cm × 2.0 cm) and was incubated at the optimal temperature (35°C for SiCut1, 45°C for SiCut2) for 48 h. (**b**) Degradation efficiency of PBS film, PLA film, PBAT film, PET film, polyester-PUR film, and polyester-PUR foam by SiCut1 and SiCut2. The reaction mixture contained 100 µL of 100 µM enzyme solution, 900 µL of Tris-HCl buffer (50 mM, pH 7.5) and films (1.2 cm × 1.2 cm) and was incubated at the optimal temperature (35°C for SiCut1, 45°C for SiCut2) for 48 h. (**c to i**) High-performance liquid chromatography-mass spectrometry (HPLC-MS) analyses of degradation products of (c) PCL film, (**d**) PBS film, (**e**) polyester-PUR foam, (**f**) PBAT film, (**g**) PET film, (**h**) PLA film, and (**i**) polyester-PUR film by SiCut1 and SiCut2. Reaction mixtures (2.0 mL) contained 50 M Tris-HCl (pH 7.5), polyester plastic films (20 mg), and enzyme (10 µM). Negative electrospray ionization tandem mass spectrometry.

To assess the capabilities of SiCut1 and SiCut2 in degrading synthetic polyester plastics, various polyester plastics, including PCL film, polybutylene succinate (PBS) film, polyester-polyurethane (PUR) foam, PBAT film, PET film, PLA film, and polyester-PUR film, were utilized as substrates for enzymatic degradation over a 48 h incubation period. The PCL films exhibited a near-total disappearance following degradation by SiCut1, while the PBS films and polyester-PUR foams demonstrated substantial fragmentation (Fig. 5a). The PBAT films, PET films, PLA films, and PUR films remained largely undisturbed after degradation by SiCut1 (Fig. 5a). The weight loss for PCL films, PBS films, and polyester-PUR foams after degradation by SiCut1 was 98.0% ± 2.2%, 12.1% ± 1.0%, and 22.2% ± 1.4%, respectively, while the weight loss for PBAT films, PET films, PLA films, and PUR films after degradation by SiCut1 was negligible (Fig. 5b; Fig. S8). In contrast, the PCL films and polyester-PUR foams exhibited substantial shrinkage following degradation by SiCut2 (Fig. 5a). However, the PBS films, PBAT films, PET films, PLA films, and PUR films demonstrated no observable change in size after degradation by SiCut2 (Fig. 5a). The weight loss for PCL films and polyester-PUR foams after degradation by SiCut2 was 21.4% ± 3.3% and 4.3% ± 1.1%, respectively, while the weight loss for PBS films, PBAT films, PET films, PLA films, and PUR films after degradation by SiCut2 was negligible (Fig. 5b; Fig. S8).

The degradation products released from the enzymatic degradation process were detected using high-performance liquid chromatography-mass spectrometry (HPLC-MS). The degradation of the PCL films by SiCut1 and SiCut2 resulted in the appearance of two distinct chromatographic peaks at 6.43 min and 5.27 min, respectively (Fig. 5c). The mass spectra corresponding to the chromatographic peak at 6.43 min exhibited two peaks at m/z of 245.1 and 259.1, representing the PCL dimer (adipic acid 6,6′-dihydroxyhexanoate) and PCL subterminal dimer (methyl adipate 6,6′-dihydroxyhexanoate), respectively (Fig. S9a). The mass spectra corresponding to the chromatographic peak at 5.27 min exhibited two peaks at m/z of 131.0 and 145.0, which represented the PCL monomer 6-hydroxyhexanoic acid and the PCL terminal monomer 6-hydroxyhexanoate methyl ester, respectively (Fig. S9b). These degradation products are indicative of enzymatic degradation of PCL film (Fig. S9c) (59).

Two chromatographic peaks were identified in the degradation products of PBS films by SiCut1 with the retention times of 15.06 and 11.87 min, respectively (Fig. 5d). The mass spectra corresponding to the chromatographic peak at 15.06 min showed one peak at m/z of 289.2, representing the PBS trimer 1,4-butanediol-bis(succinate) (SBS) (Fig. S10a) (60). The mass spectra corresponding to the chromatographic peak at 11.87 min exhibited one peak at m/z of 189.18, representing the PBS dimer 1,4-butanediol succinate (SB or BS) (Fig. S10b). These findings suggest that the ester bonds within the PBS structure were cleaved by SiCut1, leading to the release of dimers (SB) or trimers (SBS) instead of monomers (S or B) (Fig. S10c).

Seven major product peaks were identified from the degradation products of polyester-PUR foam by SiCut1 and SiCut2 (Fig. 5e). According to the mass spectra (Fig. S11), the product peaks at 23.02, 20.77, 20.02, 16.77, 14.86, and 12.84 min could be assigned to tri(adipic acid) ethylene glycol-1,4-butanediol ester, di(adipic acid) ethylene glycol-1,4-butanediol diester, di(ethylene glycol) adipate, ethylene glycol-adipic acid-1,4-butanediol ester, diethylene glycol adipate (or adipic acid oxybisethylene ester), and adipic acid, respectively (Fig. 5e; Fig. S11). The presence of these degradation products indicates that one of the polyol segments, poly(ethylene adipate-butylene adipate) diol, within polyester-PUR foam has been degraded by SiCut1 and SiCut2 (Fig. S11). In addition, the product peaks at 17.29 and 14.86 min could be assigned to ethylene glycol-adipic acid-oxybisethylene ester and adipic acid oxybisethylene ester, respectively (Fig. S11). The presence of these degradation products indicates that other polyol segments, poly(ethylene adipate-diethylene adipate) diol, within polyester-PUR foam were degraded by SiCut1 and SiCut2 (Fig. S11).

For the degradation of PBAT film, PET film, PLA film, and polyester-PUR film by SiCut1 and SiCut2, no degradation products were detected using HPLC-MS (Fig. 5f through i), in accordance with the unobservable change in weight loss (Fig. 5b).

### Molecular mechanism underlying the catalytic ability and thermostability

**Fig 6:**
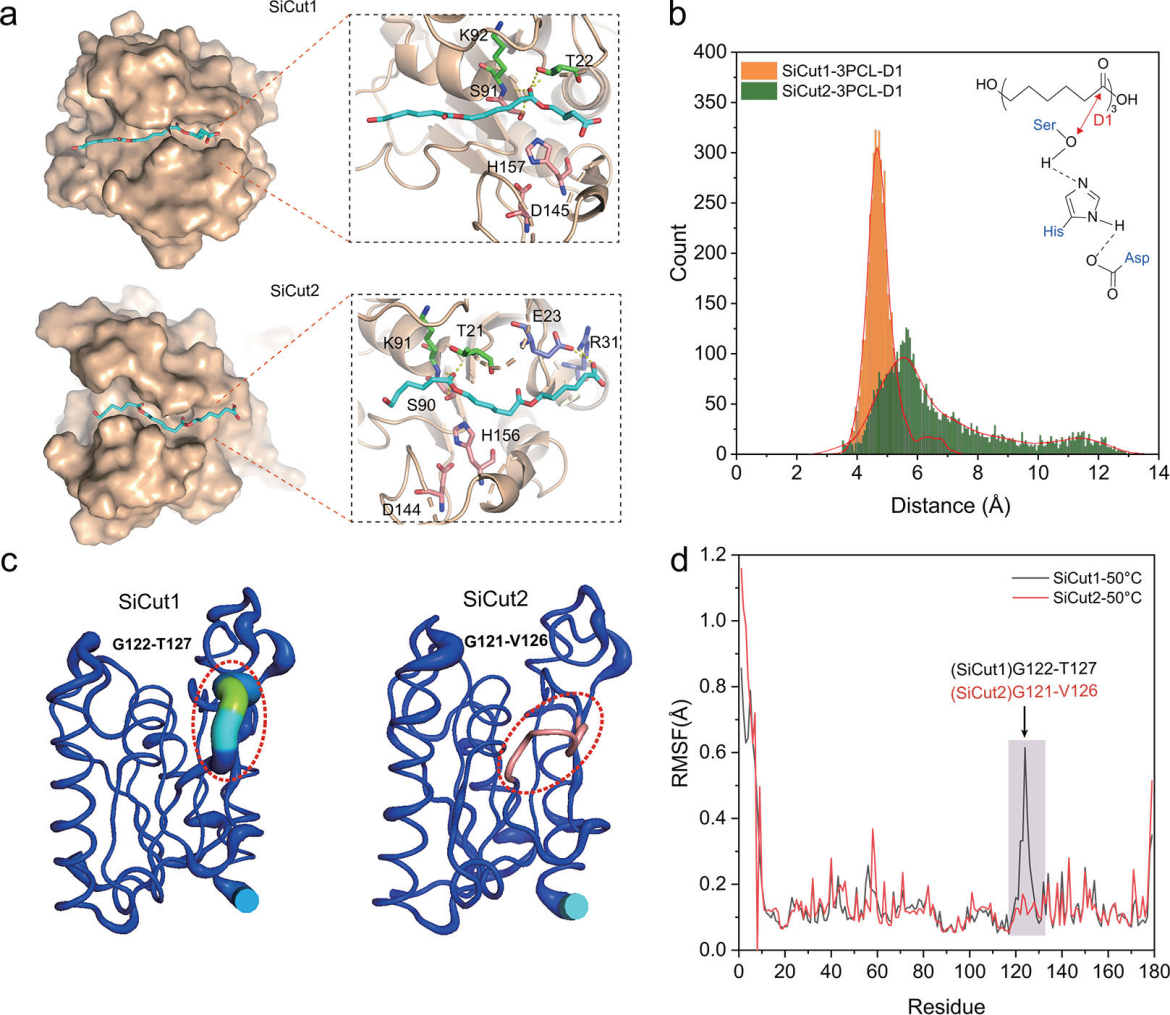
The dynamics simulation of Sicut1 and Sicut2 with substrates of PCL trimer (3PCL). (**a**) Binding model of SiCut1 and SiCut2 with 3PCL. The catalytic triad (**S91-D145-H157, S90-D144-H156**) and oxyanion hole (**T22-K92, T21-K91**) are shown as pink and green stick representations, respectively. The hydrogen-bonding interactions are shown as yellow dashed lines. (**b**) Catalytic interatomic distance between the hydroxyl oxygen atom of Ser and the carbon atom of 3PCL. (**c**) B-factor and (**d**) root-mean-square fluctuations (RMSF) of amino acid residues of SiCut1 and SiCut2 (black and red lines) were observed during the 100 ns dynamics simulation under the temperature of 323 K (50°C).

SiCut1 and SiCut2 showed different degradation abilities for various polyester plastics, but both of them exhibited the highest catalytic capability for degradation of PCL film (Fig. 5). In order to investigate the interaction of SiCut1 and SiCut2 with polyester plastic substrates, the binding mode of the substrate was obtained through molecular docking of SiCut1 and SiCut2 with PCL trimer (3PCL) (Fig. 6a). It was postulated that the catalytic triad residues of SiCut1 (S91-D145-H157) and SiCut2 (S90-D144-H156) would facilitate a nucleophilic attack on the carbonyl carbon atom of 3PCL. The backbone NH groups of T22 (T21) and K92 (K91), which constituted an oxyanion hole, formed hydrogen bonds with the negatively charged oxygen of 3PCL.

Despite the high degree of sequence identity (74.2%) exhibited by SiCut1 and SiCut2, the former displayed a higher level of activity against PCL (Fig. 5). To elucidate the underlying mechanism responsible for the observed variations in catalytic activity, a molecular dynamics simulation of the PCL trimer (3PCL)-protein complex was conducted. Because the interatomic distance (D1) between the substrate cleavage site and the catalytic serine (S91) hydroxyl oxygen has been identified as a determining factor in the occurrence of catalytic events, the distance was monitored over the course of molecular dynamics simulations (5). As illustrated in Fig. 6b, SiCut1 predominantly sampled conformations with an interatomic distance (D1) of approximately 4.8 Å, whereas SiCut2 exhibited a predominant conformational population with a longer interatomic distance (D1) of approximately 5.7 Å. The relative binding free energy of the 3PCL substrate with SiCut1 was −24.622 kcal/mol, which is 6.57 kJ/mol lower than that of SiCut2 (−18.052 kcal/mol), indicating that 3PCL can tightly bind to both SiCut1 and SiCut2 cutinase. In summary, these findings suggest that the formation of the covalent intermediate between SiCut1 and the 3PCL substrate during catalysis would be more feasible than that between SiCut2 and the 3PCL substrate. This may provide a potential explanation for the observed high level of activity of SiCut1 against PCL in comparison with SiCut2 (Fig. 5).

Furthermore, SiCut2 demonstrated superior thermal stability compared to SiCut1 at temperatures above 30°C (Fig. 3). Given that the thermal stability of enzymes is thought to be contingent upon the degree of rigidity of the protein structure, the protein B-factor and root-mean-square fluctuations (RMSF) were employed to analyze the variances in rigidity and internal motion between SiCut2 and SiCut1. It has been demonstrated that residues with high B-factors exhibit greater flexibility than those with low B-factors (61). To facilitate comparison between the two enzymes, SiCut2 and SiCut1, a relative B-factor value was selected in lieu of an absolute B-factor value, thereby indicating the relative rigidity of peptide segments within the entire protein. For example, the residue with the highest B factor was designated as 100%, and the relative B factors for the remaining residues were calculated accordingly. In SiCut2, significantly lower relative B factors were observed in the loop regions G121-V126 in comparison with the corresponding region (G122-T127) in SiCut1, indicating enhanced rigidity in these segments (Fig. 6c). RMSF values often reflect the fluctuation of individual residues during the MD simulation process. As illustrated in Fig. 6d, the loop region (G121-V126) in SiCut2 exhibits a notable decrease in fluctuation compared to the corresponding loop region (G122-T127) in SiCut1 at 50°C. This observation aligns with the relative B factor profiles depicted in Fig. 6c. Therefore, the presence of a loop region with considerably greater structural rigidity, which is attributed to the higher thermal stability of SiCut2 in comparison with SiCut1 at temperatures above 30°C, can be concluded.

## DISCUSSION

Although mealworm (*Tenebrio molitor* larvae) has been documented to consume a range of polyester plastics, and the associated gut microbiota has been demonstrated to play a role in this degradation process, the specific polyester-degrading enzymes produced by the gut microbes of mealworms have yet to be identified (39–42). In this study, a polyester-degrading yeast strain, designated as BIT-D3, was isolated from the guts of plastic-eating mealworms. Phylogenetic analysis revealed that this yeast strain represents a new species within the genus *Sakaguchia* (Fig. 1). Through a combination of genomic and transcriptomic analyses, two potential cutinases designated as SiCut1 and SiCut2 were identified as the secreted enzymes involved in the cleavage of ester bonds in polyester (Fig. 2). The amino acid sequences of SiCut1 and SiCut2 shared less than 25% identity with all previously reported cutinases (Table S4). These findings indicate that the gut microbes of plastic-eating mealworms may serve as a valuable source for the discovery of novel cutinases with the potential to facilitate the degradation of polyesters.

Intriguingly, the yeast strain BIT-D3 may degrade polyester plastic when given citrate, pyruvate, or succinate as additional carbon sources. However, it cannot degrade the polyester when glucose or lactose are utilized as additional carbon sources (Fig. S2). Citrate, pyruvate, or succinate, being an intermediate of the tricarboxylic acid cycle, can directly enter this cycle, thereby enhancing cellular energy metabolism and activating certain pathways related to the expression of enzymes that degrade polyesters (62). In contrast, glucose, the most common and preferential carbon source, may inhibit the activation of other metabolic pathways and the expression of genes involved in secondary metabolism (62, 63). Lactose can undergo further hydrolysis to yield glucose, which also elicits regulatory responses that impede the metabolism of alternative carbon sources (64, 65). It can be reasonably inferred that both glucose and lactose are likely to induce carbon catabolite repression (CCR), which may result in the inhibition of essential enzyme systems or metabolic pathways required for polyester degradation (64, 65). It has been demonstrated that the transcription factor Mig1 is the key regulator for implementing CCR in yeast (66). In the presence of elevated glucose levels, the protein phosphatase Glc7 dephosphorylates Mig1-PO_4_ into Mig1, thereby enabling the nuclear translocation of Mig1. There, it recruits the co-repressor proteins Tup1 and Ssn6 to form the co-repressor complex, which binds to a consensus DNA sequence (5′-SYGGRG-3′) in the promoters of targeted genes, thereby repressing their transcription (66). The consensus DNA sequence 5′-SYGGRG-3′ was identified in the promoter region of the SiCut1- and SiCut2-encoding genes (*A02567*, *A02569*) (Fig. S12). This suggests that the expression of SiCut1 and SiCut2 may also be subject to CCR regulation. This phenomenon provides insight into the necessity of adding suitable carbon sources to mitigate CCR and stimulate the expression of plastic-degrading enzymes during the isolation of plastic-degrading microorganisms from the environment.

Both SiCut1 and SiCut2 demonstrated robust activity toward PCL film, PBS film, and polyester-PUR foam, yet exhibited limited activity towards other polyester plastics (Fig. 5). This degradation profile for polyester differed from that of the known yeast-derived cutinases, which can also exhibit strong activity toward PBAT film and PLA film (28, 67). Previously reported yeast-derived cutinases have been observed to possess a highly conserved S-D-H catalytic triad with a GYSQG motif (Fig. S4). In contrast, both SiCut1 and SiCut2 contain a catalytic triad with a GYSKG motif, where glutamine (Q) is substituted with lysine (K) (Fig. S4). Glutamine (Q) is a polar, uncharged amino acid with a side chain amide group capable of forming stable hydrogen bonds within the catalytic center, which helps maintain the proper conformation of the active site or assists in substrate positioning (68). However, lysine (K) has a longer side chain and carries a positive charge. The replacement of glutamine (Q) with lysine (K) may result in alterations to the active site conformation, potentially disrupting the precise positioning of serine and reducing catalytic efficiency (69). It can thus be posited that the GYSKG motif represents a distinctive feature of the SiCut1 and SiCut2 cutinases, which enables their capacity to degrade polyester polymers to diverge from that of other yeast-derived cutinases. Further mutation experiments will be conducted to provide evidence for this hypothesis.

Our findings indicate that SiCut1 exhibits superior catalytic activity for the hydrolysis of polyester plastics than SiCut2 (Fig. 5), whereas SiCut2 displays enhanced thermal stability compared to SiCut1 (Fig. 3). The enzyme engineering strategy of domain swapping, which allows the construction of chimeric proteins using homologs with disparate characteristics, has the potential to enhance the overall performance of the chimeric enzyme (70–72). In further work, the sequences of SiCut1 and SiCut2 can be used to create chimeric proteins through the above strategy. The creation of these chimeric proteins could result in the development of a novel, highly active, and thermally stable engineered enzyme for plastic degradation. The engineered enzymes may find greater application in the enzymatic recycling of plastic waste, thereby promoting the development of a plastic circular economy.

### Conclusions

Enzymatic degradation and recycling can be an effective strategy for mitigating the environmental impact of plastic waste. The discovery of novel plastic-degrading enzymes is an important step in the development of enzymatic degradation and recycling strategies for plastic waste. In the present study, two new cutinases, named SiCut1 and SiCut2, were identified from a yeast strain *Sakaguchia* sp. BIT-D3, which was isolated from the gut of plastic-eating mealworm. Their amino acid sequences have less than 25% identity to all previously reported cutinases and reveal a conserved S-D-H catalytic triad with an unique GYSKG motif. Both enzymes showed strong activity against apple cutin and short-chain fatty acid esters of *p*-nitrophenol and glycerol, thereby supporting their classification as bona fide cutinases. SiCut1 and SiCut2 have temperature ranges of 10°C to 50°C and 10°C to 70°C, with 35°C and 45°C being the optimal temperatures, respectively. SiCut1 and SiCut2 demonstrated efficient degradation toward PCL film, PBS film, and polyester-PUR foam. The present study introduces two new and distinct biocatalysts for depolymerization and recycling of plastic waste. In the future, domain swapping, an enzyme engineering method, could be used to create chimeric enzymes based on the SiCut1 and SiCut2 sequences. The production of these chimeric proteins could lead to the generation of a new, highly active, and thermally stable enzyme for plastic degradation and recycling.

## MATERIALS AND METHODS

### Testing materials

The polyester plastics used in the experiments were as follows: PCL pellets were purchased from Pensieve Technology (Wuhan, China); poly(butylene succinate) film (BioPBS) was provided by Mitsubishi Chemical Corporation (Tokyo, Japan); PLA pellets were purchased from Hisun Biomaterials Co., Ltd. (Taizhou, China); PBAT pellets were purchased from BASF (Ludwigshafen, Germany); amorphous PET film was purchased from Goodfellow (Cambridge, UK); polyester-PUR film was provided by Wanhua Chemical Group (Yantai, China); and polyester-PUR foam was provided by Daye Tengfei Foam Factory (Changzhou, China). The characteristic parameters of all polyester plastics used for biodegradation are listed in Table S1. The structural formulas of these plastic films and polyester-PUR foam used in this study are exhibited in Fig. S1.

For the preparation of the plastic emulsion, the plastic pellets (1.8 g) were first dissolved in 40 mL of dichloromethane and heated in a 60°C water bath for 1 h. After the complete dissolution of all pellets in the solvent, 50 mL of distilled water was added to the plastic-dissolved solvent, resulting in a layered mixture with the aqueous phase on the upper side and the organic phase on the lower side. Subsequently, 2 mL of 2% emulsifying agent (plysurf A210G, Dai-ichi Kogyo Seiyaku Co., Ltd., Japan) was added as a surfactant. This layered solution underwent 10 min of sonication in an oscillator (JY96-IIN, Ningbo Scientz Biotechnology Co., Ltd.) at 30% amplitude with pulses every 15 s to transfer the plastic components dissolved in the organic phase to the water layer. Following sonication, the solvent was completely evaporated using a 60°C magnetic stirrer in a fume hood for 3 h. After evaporation of the solvent, a 1.8% (g/vol) plastic emulsion was formed by adding 50 mL of distilled water.

The films of PCL, PLA, and PBAT were prepared using the conventional solvent-cast method. The plastic pellets (1.8 g) were dissolved in 50 mL of chloroform and heated in a water bath at 60°C until all the pellets were dissolved in the solvent. The solvent-dissolved plastic pellets were then poured into a polytetrafluoroethylene dish, followed by drying at room temperature in a fume hood until the solvent completely evaporated.

### Isolation of yeasts from the gut of plastic-eating mealworms

Mealworms (growth age at approximately 3–4 instars) were purchased from Daxing insect-breeding plants in Beijing, China. About 200 mealworms were immersed in 75% ethanol for 5 min to eliminate surface microorganisms and then rinsed two times with sterile saline water (NaCl, 0.85%, wt/vol) prior to gut excision. Their guts were sterilely drawn out and pooled into a 50 mL centrifuge tube containing 30 mL of sterile saline water. This mixture was vortexed for 5 min and then centrifuged at 800 rpm for 5 min to remove gut tissues. The remaining suspension, used as a microbial inoculum, was transferred into 150 mL Yeast-Malt (YM) medium (0.3 g/L yeast extract, 0.3 g/L malt extract, 0.5 g/L peptone, 1 g/L glucose, pH adjusted to 3.5 with HCl). The culture was incubated at 250 rpm and 30°C for 3 days. Thereafter, 1 mL of the above culture is withdrawn and added into sterile saline water for serial dilution by a factor of 10. From this, 1 mL of the 10^−4^, 10^−5^, and 10^−6^ dilutions is spread onto a YM agar plate (0.3 g/L yeast extract, 0.3 g/L malt extract, 0.5 g/L peptone, 1 g/L glucose, 1.5 g/L agar, and 100 mg/L chloramphenicol), followed by incubation at 25°C for 3 days. Individual colonies on the plates were streaked for a subculture to obtain a pure culture. The isolated yeast strains were stored in a YPD medium (10 g/L yeast extract, 20 g/L peptone, 20 g/L glucose) containing 30% (vol/vol) sterile glycerol at −80°C.

For taxonomic identification of the obtained pure cultures, genomic DNA from the pure cultures was extracted using the TIANamp Yeast DNA Kit (Catalog no. DP307, TIANGEN). The extracted genomic DNA served as the template for PCR amplification to obtain the ITS rDNA, 26S D1/D2 rDNA, and 18S rDNA sequences with the primers shown in Table 3. DNA sequencing of the PCR products was performed by Genewiz Biotechnology Co., Ltd. (Beijing, China). The full-length sequences were uploaded to the NCBI website for homology sequence alignment to identify typical strains with high sequence similarity. Phylogenetic trees were constructed using the neighbor-joining method in MEGA 11 (73), and the stability of the phylogenetic trees was assessed using the Bootstrap method. The cells’ morphology was observed using a scanning electron microscope (SEM, SU8010, Hitachi) after 48 h incubation on YPD agar at 30°C.

**TABLE 3:** Primers and SiCut1 and SiCut2 sequences used in this study

Name	Sequence^*a*^	Description
ITS-F	GTCGTAACAAGGTTTCCGTAGGTG	Amplification of the ITS
ITS-R	TCCTCCGCTAATTGATATGC	
26S-F	GCATATCGGTAAGCGGAGGAAAAG	Amplification of the 26S
26S-R	GGTCCGTGTTTCAAGACGG	
18S-F	TCCTCTAAATGACCAAGTTTG	Amplification of the 18S
18S-R	GGAAGGGRTGTATTTATTAG	
SiCut1-F	AGAGAGGCTGAAGCTTACGTAGAATTCGCTGCCATCGCAGAACGACAGTCTT	Amplification of SiCut1
SiCut1-R	CATCGCCGGCTGCTACAGGGCCTAAGCGGCCGCGAATTAATTCGCCTTAGAC	
SiCut2-F	AGAGAGGCTGAAGCTTACGTAGAATTCACAGCCATCGGAGAGAGACAGCTTG	Amplification of the gene sequence of SiCut2 from BIT-D3
SiCut2-R	CATCGCCGAATGCTTCAACGCTTGAGCGGCCGCGAATTAATTCGCCTTAGAC	
5′AOX1	GACTGGTTCCAATTGACAAGC	For *Pichia* vectors with AOX1 promoter, forward primer
3′AOX1	GCAAATGGCATTCTGACATCC	For *Pichia* vectors with AOX1 terminator, reverse primer
SiCut1	AAIAERQSSACAPLELYHAAGTSESGLGTVGRAFQTALPREVQGATVSPLNYNTNAEY SSTVTAGAQTFQQLLTSRAAACPNTKFVLSGYSKGALVVHKVTLPSAVQSKVVAITVFGD PQAGQFGATTVPINNKACFFSFCNRGDTFCDRGGNFAAHLAYATDGSVTTASRNIAGCYRA	Amino acid sequence of SiCut1
SiCut2	TAIGERQLGCAPLELFHAAGTSEVGQGIIGRAYQSALPRAVSGATVTPVIYSTIAEYFTTVRAG AQTMQRQLTSRAAACPNTKFVLSGYSKGALVVHEVKLPAAIQSKVVAVTLFGDPKNGQFGSSVV PINNKACFFSFCNRGDVFCDRGISLAAHLAYATDGSVTTAARNIAECFNA	Amino acid sequence of SiCut2

^*a*^ Restriction enzyme sites are underlined.

### Evaluation of polyester-degrading ability of yeast isolates

A single colony was inoculated into YPD medium and cultured at 250 rpm and 30°C for 24 h. The cells were harvested and re-suspended in sterile saline water to an OD_600_ of approximately 1.0. Subsequently, 2.5 µL of the cell suspension was carefully slotted into the center of the solid assay plates, which had been prepared with the following components: 0.2% (vol/vol) PCL emulsion, 99.8% (vol/vol) yeast-extract salts (YES) medium (0.04 mg/L yeast extract, 0.4 g/L NH_4_Cl, 0.5 g/L KH_2_PO_4_, 1.0 g/L K_2_HPO_4_, 0.5 g/L MgSO_4_, 2.0 mg/L MnCl_2_·4H_2_O, 0.04 mg/L CaCl_2_·6H_2_O, 0.04 mg/L FeCl_3_·6H_2_O, 0.04 mg/L ZnCl_2_, 0.04 mg/L Na_2_MoO_4_·2H_2_O, 0.04 mg/L CuCl_2_·2H_2_O), 15 g/L agar, and 20 g/L carbon source (each of pyruvate, citrate, succinate, lactose, or glucose). The inoculated plates were then incubated in an inverted position at 30°C for 14 days to observe the formation of clear zones.

Validation of polyester degradation by the isolates in liquid medium was conducted as follows: strain BIT-D3 was cultivated in 40 mL of YES liquid medium with 20 g/L citrate at 30°C for 48 h. The culture was centrifuged at 10,000 × *g* for 10 min to separate the cellular debris. The resulting supernatant, which contained crude extracellular enzymes secreted by strain BIT-D3, was utilized directly for the evaluation of PCL-degrading activity. A volume of 5 mL supernatant was combined with 55 µL PCL emulsion (1.8%, vol/vol), and the resulting mixtures were incubated at 30°C. Negative controls were subjected to thermal inactivation at 100°C for a period of 2 h before incubation. The change in optical density was quantified at 600 nm using a universal microplate spectrophotometer (Multiskan Ascent, Thermo Fisher Scientific). Finally, all post-degradation samples were lyophilized and analyzed using FTIR spectroscopy (Nicolet 6700, Thermo Fisher Scientific) to confirm changes in the chemical structure.

### Genomic and transcriptomic sequencing of strain BIT-D3

For genomic sequencing, the strain BIT-D3 was inoculated into an Erlenmeyer flask containing 50 mL of YPD medium and then incubated in a shaking incubator at 250 rpm and 30°C for 12 h. Genomic DNA was extracted from the harvested cells using the TIANamp Yeast DNA Kit (Catalog no. DP307, TIANGEN). The genome of BIT-D3 was sequenced at Novogene Bioinformatics Institute (Beijing, China), using the PacBio Sequel and Illumina NovaSeq PE150 system for massive parallel sequencing. The genome assembly was performed with SMRT Link v5.0.1. The coding genes were predicted with the Augustus 2.7 program.

For transcriptomic sequencing, the BIT-D3 strain was inoculated into an Erlenmeyer flask containing 50 mL of liquid assay medium that had been prepared with the following components: 0.2% (vol/vol) PCL emulsion, 99.8% (vol/vol) YES medium, and 20 g/L citrate. In the control group, 20 g/L citrate was replaced with 20 g/L glucose. All groups had three biological replicates and were incubated in a shaking incubator at 250 rpm and 30°C for 48 h. Total RNA was extracted from the harvested cells using the Thermo Scientific GeneJET RNA Purification Kit (K0731) and subsequently sent to Novogene Bioinformatics Institute (Beijing, China) for library construction and sequencing. High-throughput sequencing was performed on the Illumina NovaSeq 6000 platform using a paired-end 150 bp (PE150) sequencing strategy.

### Recombinant expression proteins in *Pichia pastoris*

The cDNA was generated by reverse transcriptase PCR from RNA of yeast strain BIT-D3 using a GoScript Reverse Transcription kit (Promega, A5002). The cDNA was then used as the template to amplify the target genes (Table 3). The PCR products were recovered using the TIANgel Purification Kit (TIANGEN, #DP219). Next, the PCR products of the target gene and the linearized plasmid pPIC9k were ligated by Gibson Assembly Cloning Kit (NEB, #E5510S). The ligated products were introduced into *Escherichia coli* DH5α cells. Positive recombinant plasmids were then introduced into *P. pastoris* GS115 by electroporation (1.5 kV, 5 ms) after being linearized with *Sal*I. Colonies of recombinant *P. pastoris* GS115 were grown on a histidine-deficient minimal dextrose (MD) agar plate (13.4 g/L yeast nitrogen base, 20 g/L glucose, 0.4 mg/L biotin, 15 g/L agar). Then, colonies of recombinant *P. pastoris* GS115 were randomly selected and screened on YPD plates containing geneticin G418 at increasing concentrations of 1.0, 2.0, 3.0, and 4.0 mg/mL. Transformants can resist higher concentrations of G418 that might have multi-copies of integration into the *P. pastoris* genome, which could potentially lead to higher levels of protein expression. Then, the positive single colonies were picked from YPD plates containing 4 mg/mL G418 and were spotted onto both histidine-deficient minimal methanol (MM) agar plate (13.4 g/L yeast nitrogen base, 0.5% (vol/vol) methanol, 0.4 mg/L biotin, 15 g/L agar) and MD agar plate. Mut^+^ (methanol utilization plus) transformants, which exhibited similar growth rates on both MM and MD plates, can be cultured with methanol as the carbon source and were selected for further experiments. After plate screening, single colonies were verified by colony PCR using the universal primers 5′AOX1 and 3′AOX1 (Table 3).

Recombinant *P. pastoris* GS115 cultures were separately inoculated into 10 mL YPD for activation and grown for 24 h at 30°C and 250 rpm. Next, 1 mL cultures were transferred to 50 mL buffered glycerol-complex medium (BMGY) (10 g/L yeast extract, 20 g/L peptone, 10 g/L glycerol, 13.4 g/L yeast nitrogen base, 5 g/L (NH_4_)_2_SO_4_, 0.4 mg/L biotin, 11.8 g/L K_2_HPO_4_, 7 g/L KH_2_PO_4_) and inoculated for another 24 h under the same conditions. Cells were then harvested by centrifugation at room temperature for 5 min at 3,000 *× g*. BMGY culture media was replaced with 100 mL buffered methanol-complex medium (10 g/L yeast extract, 20 g/L peptone, 1% (vol/vol) methanol, 13.4 g/L yeast nitrogen base, 5 g/L (NH_4_)_2_SO_4_, 0.4 mg/L biotin, 11.8 g/L K_2_HPO_4_, 7 g/L KH_2_PO_4_) and the culture was grown for another 7 days at 30°C with constant shaking; 100% methanol was added every 24 h in order to maintain a concentration of 2% methanol. Proteins secreted into the supernatant were obtained by centrifuging the fermentation broth (8,000 × *g*, 5 min, 4°C). The supernatant was filtered through a 0.22 µm Millipore filter to remove residual cells and diafiltered approximately fivefold by using a filtration membrane (Millipore) with a molecular weight cut-off of 3 kDa. The protein concentration was determined by the bicinchoninic acid (BCA) protein assay kit (Solarbio, China).

### Biochemical characterization of recombinant enzymes

To determine the effect of pH on enzyme activity, the standard enzyme assay was conducted using various buffers exhibiting a pH value range from 3.0 to 12.0, including 50 mM sodium citrate buffer (pH: 3.0 to 5.0), 50 mM sodium phosphate (pH: 6.0 to 8.0), 50 mM Tris-HCl (pH: 7.0 to 9.0), and 50 mM glycine-NaOH buffer (pH: 9.0 to 12.0). The reaction mixture (100 µL) contained 40 µL of each buffer, 55 µL of PCL emulsion, and 5 µL of enzyme solution (10 µM). The reaction was performed at ambient temperature, and the reaction rates were determined at 600 nm using a universal microplate spectrophotometer (Multiskan Ascent, Thermo Fisher Scientific). For the pH stability test, the reaction mixture (45 µL) contained 40 µL of each buffer, and 5 µL of enzyme solution (10 µM) was incubated at ambient temperature for 8 h. After that, the residual activity was measured using the above assay method.

The effect of temperature on enzymatic activity was tested at the temperature ranging from 0°C to 90°C. The reaction mixture (100 µL) contained 40 µL of the optimal buffer, 55 µL of PCL emulsion, and 5 µL of enzyme solution (10 µM). The reaction rates were determined at 600 nm using a universal microplate spectrophotometer (Multiskan Ascent, Thermo Fisher Scientific). For the thermal stability test, the reaction mixture (45 µL) contained 40 µL of the optimal buffer and 5 µL of enzyme solution (10 µM) was incubated at temperatures of 20°C, 30°C, 40°C, 50°C, 60°C and 70°C for 8 h. After that, the residual activity was measured using the above assay method at ambient temperature.

Half-life assays were used to further confirm the thermal stability of the enzyme. The reaction mixture (45 µL) containing 40 µL of the optimal buffer and 5 µL of enzyme solution (10 µM) was incubated at temperatures of 20°C, 30°C, 40°C, 50°C, 60°C, and 70°C. The enzyme samples were withdrawn every 12 h for analyzing the residual enzyme activity. The enzyme deactivation was described considering a first-order decay. The half-life of enzymes was calculated by the following formulas:


$$\begin{array}{cc} ln\left(\frac{E_{t}}{E_{0}}\right)=-k_{d}t, & \\ t_{1/2}=ln\left(2\right)/k_{d}, \end{array}$$

where *E*_*t*_ is the residual activity after time *t*, *E*_0_ is the initial activity of cutinase, *k*_d_ is the deactivation rate constant, and *t*_1/2_ is the half-life.

### Enzymatic assay for *p*-nitrophenol esters, triglycerides, and cutin

The measurement of enzyme activity toward different *p*-nitrophenyl (*p*NP) esters was performed using the substrate of *p*NP-acetate (C2), *p*NP-butyrate (C4), *p*NP-hexanoate (C6), *p*NP-octanoate (C8), *p*NP-decanoate (C10), *p*NP-laurate (C12), *p*NP-myristate (C14), and *p*NP-palmitate (C16), respectively. The reaction mixture (100 µL) contained 90 µL of enzyme solution (1 µM in 50 mM Tris-HCl buffer at pH 7.5) and 10 µL of *p*NP ester solution (10 mM). The formation of *p*-nitrophenol over time at the optimal temperature of enzymes was monitored with a spectrophotometer at 420 nm. A reaction mixture with buffer instead of enzyme was used as a negative control. The activity of the control was subtracted from the activity of the sample to obtain the overall value of the enzymatic activity. The kinetic curves were used to calculate the reaction constants of *K_m_* and *K_cat_* for each substrate using GraphPad Prism software. All enzyme assays were performed in triplicate, and the activities shown are averages of the values from three independent experiments.

Activity for triglycerides (triacetin, tributyrin, tricaproin, tricaprylin, tricaprin, trilaurin) was measured using the titration method described by Kleeberg et al. (31). Enzyme activity was calculated from the amount of sodium hydroxide used to maintain pH at 5.5. All enzyme assays were performed in triplicate, and the activities shown are averages of the values from three independent experiments.

Red Fuji apple cutin was prepared as described by Walton and Kolattukudy (74). Peels from fresh apples were collected from fruit supermarkets (approximately 0.5 kg). The cuticles were separated from the peels by boiling the peels under 100°C with an aqueous solution of oxalic acid (4 g/L) and ammonium oxalate (16 g/L) for about 4 h until the pulp was removed. Then, the cuticles were extracted with 500 mL CHCl_3_-CH_3_OH (2:1, vol/vol) for 24 h to remove lipids, waxes, and other impurities. The purified cuticles were thoroughly washed with distilled water and dried completely at 35°C. Then the dried cuticles were treated with a solution of 5 g/L cellulase (#C1184, Sigma Chemical Co.) and 1 g/L pectinase (#P4716, Sigma Chemical Co.) in acetate buffer (50 mM, pH 4.0) at 30°C for 14 h to remove the cellulose and pectin components. The residual cuticles after enzymatic treatment were collected by filtration and were thoroughly washed. The steps of solvent extraction and enzyme treatment were repeated twice. Finally, 20 g of residual cuticles were obtained as crude cutin, which was used to evaluate cutinase activity.

To measure cutinase activity, 100 mg of cutin was suspended in 2 mL of Tris-HCl buffer (50 mM, pH 7.5), and treated with 100 µL of 100 µM enzyme solution of SiCut1 or SiCut2. The mixture was incubated for 24 h at the optimal temperature of each enzyme. In addition, the positive control group was treated with TfCut at similar concentrations in potassium phosphate buffer (25 mM, pH 8.0) (32). In particular, we used the 16-hydroxyhexadecanoic acid (#H862331, Shanghai Macklin Biochemical Co., Shanghai, China) as a standard to analyze the product information in the samples. The buffer-treated sample was used as a negative control. After the reaction, the remaining cutin was removed by centrifugation (220 rpm, 10 min). The reaction was terminated by adding 25% (vol/vol) acetic acid to the resulting supernatant. Degradation products of cutin were extracted by the addition of chloroform (1:1, vol/vol). The extract was dried by evaporation with a nitrogen stream and then exposed to 500 mL of pyridine (Macklin) and 0.2 mL of *N*-methyl-*N*-(trimethylsilyl) trifluoroacetamide (MSTFA, #N814323, Macklin, Shanghai, China) for 1 h at 70°C. The excess silylating agent was removed via evaporation using a stream of nitrogen gas. The sample was then dissolved in 0.5 mL of hexane and analyzed via GC-MS as previously published (29). A Trace 1310 Gas-Chromatography System with ISQ 7000 Single Quadrupole Mass Spectroscopy detector was used to analyze the hydrolysis products. Samples (10 µL) were injected directly onto a 30-m Rtx5-MS capillary column (Restek) using a splitless injection and a He carrier gas. The injector temperature was 250°C. The separations were performed using a temperature profile starting at 80°C (holding for 2 min), followed by a 10°C/min temperature gradient to 150°C and holding for 5 min. Then, the temperature was increased to 300°C at a rate of 5°C/min and held for an additional 2 min. The hydrolysis products were identified by electron impact ionization (70 eV, source temperature 200°C). The obtained mass spectra of each compound were compared to the mass spectra of the NIST MS database within the program.

### Enzymatic assay for polyester plastics

A total of seven types of plastics, including PCL films, PBS films, polyester-PUR foams, PBAT films, PET films, PLA films, and polyester-PUR films (Table S1), were cut into dimensions of 1.2 cm × 1.2 cm. Two pieces of each plastic, with an initial weight of approximately 25, 15, 74, 29, 76, 27, and 56 mg, respectively, were utilized in the degradation test. A total of 900 µL of 50 mM Tris-HCl buffer (pH 7.5), 100 µL of 100 µM enzyme solution, and films were added to each reaction mixture. The mixture was incubated for 48 h at the optimal temperature of each enzyme. After the reaction, the plastic films were washed with water and dried at 40°C, and then the weight was measured by a laboratory balance with an accuracy of 0.01 mg (QUINTIX35-1CN, Sartorius, Germany). The weight loss before and after the reaction was recorded to calculate the degradation rate of various plastic films by the enzymes.

After the reaction, the liquid samples were analyzed using a high-performance liquid chromatograph (1290 series, Agilent Technologies) with a C18-MS-II column (COSMOSIL, 4.6ID × 250 mm, Tokyo) and a Q-TOF mass spectrometer (6545, Agilent Technologies) (HPLC-MS). A 10 µL sample was injected at 40°C. For the detection of the degraded products of PCL film and PBS film, the elution solvents were A (water + 0.1% formic acid) and B (acetonitrile + 0.1% formic acid), with the following gradient: 5%–44% B over 12 min, 44%–70% B over 3 min, and 70% B for 3 min. For the detection of the degraded products of PLA film and polyester-PUR film/foam, the elution solvents were A (water + 0.1% formic acid) and B (acetonitrile + 0.1% formic acid), with the following gradient: 5%–40% B over 15 min, 40%–80% B over 10 min, and 100% B for 5 min. For the detection of the degraded products of PBAT film and PET film, the elution solvents were A (water + 0.1% formic acid) and B (methanol + 0.1% formic acid), with the following gradient: 10%–50% B over 8 min, 50%–90% B over 2 min. The compounds were detected using negative mode mass spectrometry with a mass range of 100–1,000 m/z. The settings of the electrospray ionization tandem mass spectrometry were as follows: gas temperature 300°C, drying gas 8 L/min, nebulizer 18 psig, sheath gas 350°C, 12 L/min, capillary voltage 3 kV, and fragmentor 180 V.

### Modeling, molecular docking, and molecular dynamics simulation

The 3D structures of SiCut1 and SiCut2 were obtained by the predictions of AlphaFold v2.0 (75). The resulting structures were visualized and analyzed in more detail using PyMOL v2.6.0. The 2D-dimensional chemical structures of the ligands were first drawn in ChemDraw v15.0 (PerkinElmer) and converted into 3D-dimensional structures with Open Babel v3.1.0 on the online platform Wecompute (https://wemol.wecomput.com/). The initial complex structures were obtained using molecular docking with AutoDock v4.2.6 using the docking pocket that was produced by Proteins.plus (https://proteins.plus/). Subsequently, the best binding compound pose was determined based on the scoring functions and conformational analysis.

Molecular dynamics simulations and analyses of the complex structures with the best binding compound pose were performed using GROMACS on the online platform Wecompute (https://wemol.wecomput.com/). The Amber force field and the tip3p water model were used with a 10 Å distance between the box edges and solute surface. The ligand and receptor were hydrogenated, and the pKa of the receptor protein residues was predicted and protonated according to the specific pH value. Bond charge corrections were applied to the ligand parameterization, and Na^+^ and Cl^−^ ions were added to neutralize the system (76). Atomic electrostatic interactions are calculated using the particle-mesh Ewald method. The topologically rigid structure was not constrained, and the system was equilibrated for 100 ps under NPT conditions at ~298 K. The simulation step was 2 fs, and the total simulation time was 100 ns at 298 and 323 K. Finally, GROMACS tools were used to extract and analyze the 0–100 ns simulation trajectories, including root-mean-square deviation and RMSF.

Here, the molecular mechanics Poisson-Boltzmann surface area methods were used to estimate the binding free energies as they can quickly and efficiently calculate free energies from molecular dynamics (77). B-factors were calculated based on the value of RMSF using the function proposed by Kuzmanic and Zagrovic (61). The amino acids were visualized according to their B-factor values using PyMOL. The unresolved atoms of the amino acids close to the N-terminus and C-terminus were excluded.

## Data Availability

The ITS rDNA, 26S D1/D2 rDNA, and 18S rDNA sequences of strain BIT-D3 have been deposited in the NCBI database with the accession number PQ164392. The amino acid sequences of SiCut1 and SiCut2 have been deposited in the NCBI database with the accession numbers PQ164790 and PQ164791, respectively.

## SUPPLEMENTAL MATERIAL

Supplemental material aem.02562-24-s0001.docx Figures S1 to S12; Tables S1 to S4.

ASM does not own the copyrights to Supplemental Material that may be linked to, or accessed through, an article. The authors have granted ASM a non-exclusive, world-wide license to publish the Supplemental Material files. Please contact the corresponding author directly for reuse.
