# Supplementary material for: Discovery of two novel cutinases from a gut yeast of plastic-eating mealworm for polyester depolymerization
Source: Appl Environ Microbiol. 2025 Apr 2;91(4):e02562-24. doi: 10.1128/aem.02562-24 (PMC12042792; doi:10.1128/aem.02562-24)
Supplement: Supplemental material — Figures S1 to S12; Tables S1 to S4. [file aem.02562-24-s0001.docx]

*Supplementary materials for*

**Discovery of two novel cutinases from a gut yeast of plastic-eating mealworm for polyester depolymerization**

Tong Huang^1, #^, Jingya Zhang^1, #^, Xuena Dong^1^, Yu Yang^1, *^

^1^School of Life Science, Beijing Institute of Technology, Beijing 100081, P. R. China.

#These authors contributed equally to this work

*Corresponding author: Dr. Yu Yang

Phone & Fax: +86-10-68911329.

E-mail: [yooyoung@bit.edu.cn](mailto:yooyoung@bit.edu.cn)

ORCID: 0000-0002-4663-9753

Corresponding author address: Department of Biology, School of Life Science, Beijing Institute of Technology, 5 South Zhongguancun Street, Beijing 100081, P. R. China.

*12 Figures and 4 Tables*


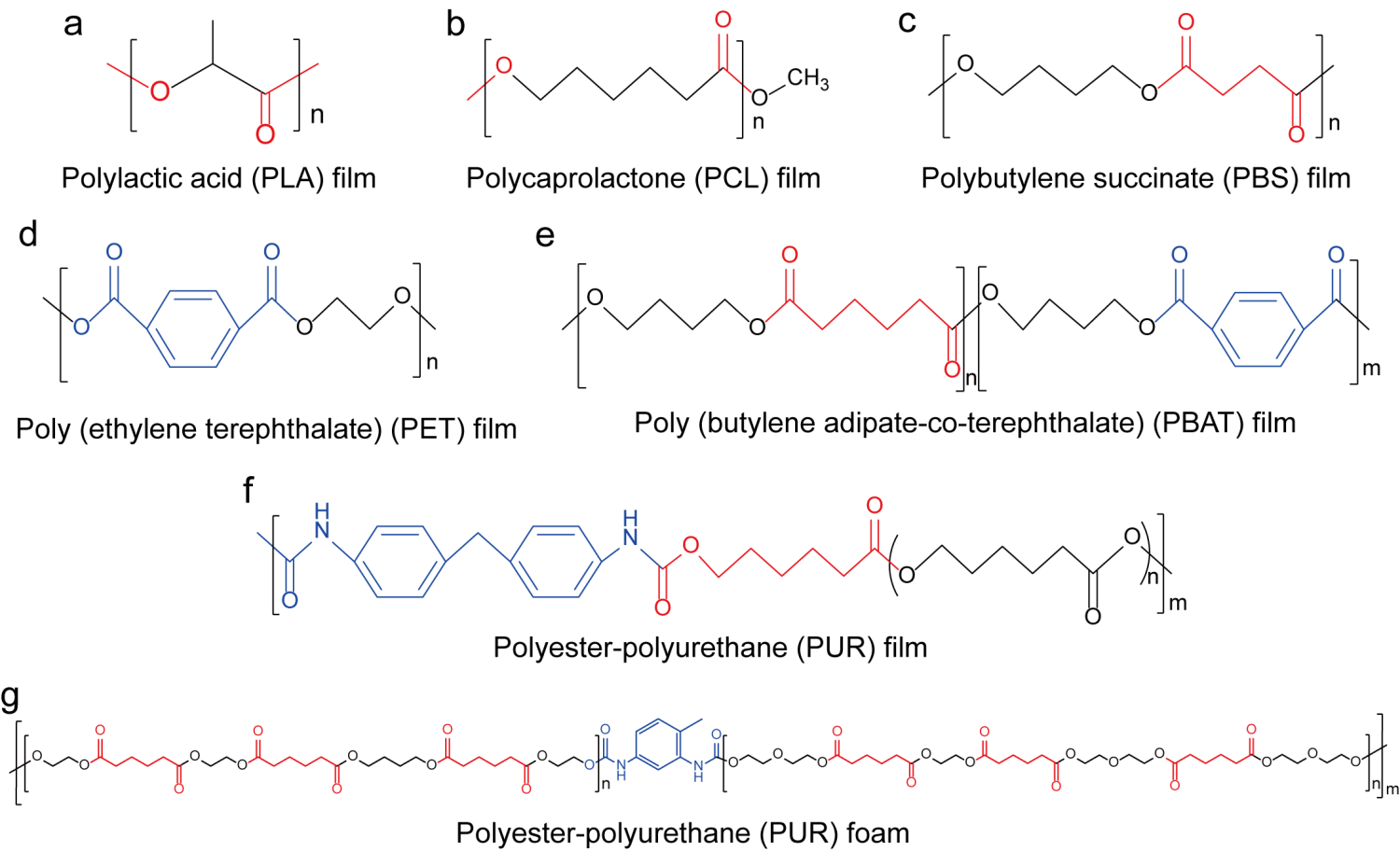


**Figure S1.** Structural formulas of plastic films and polyester-PUR foam used in this study.


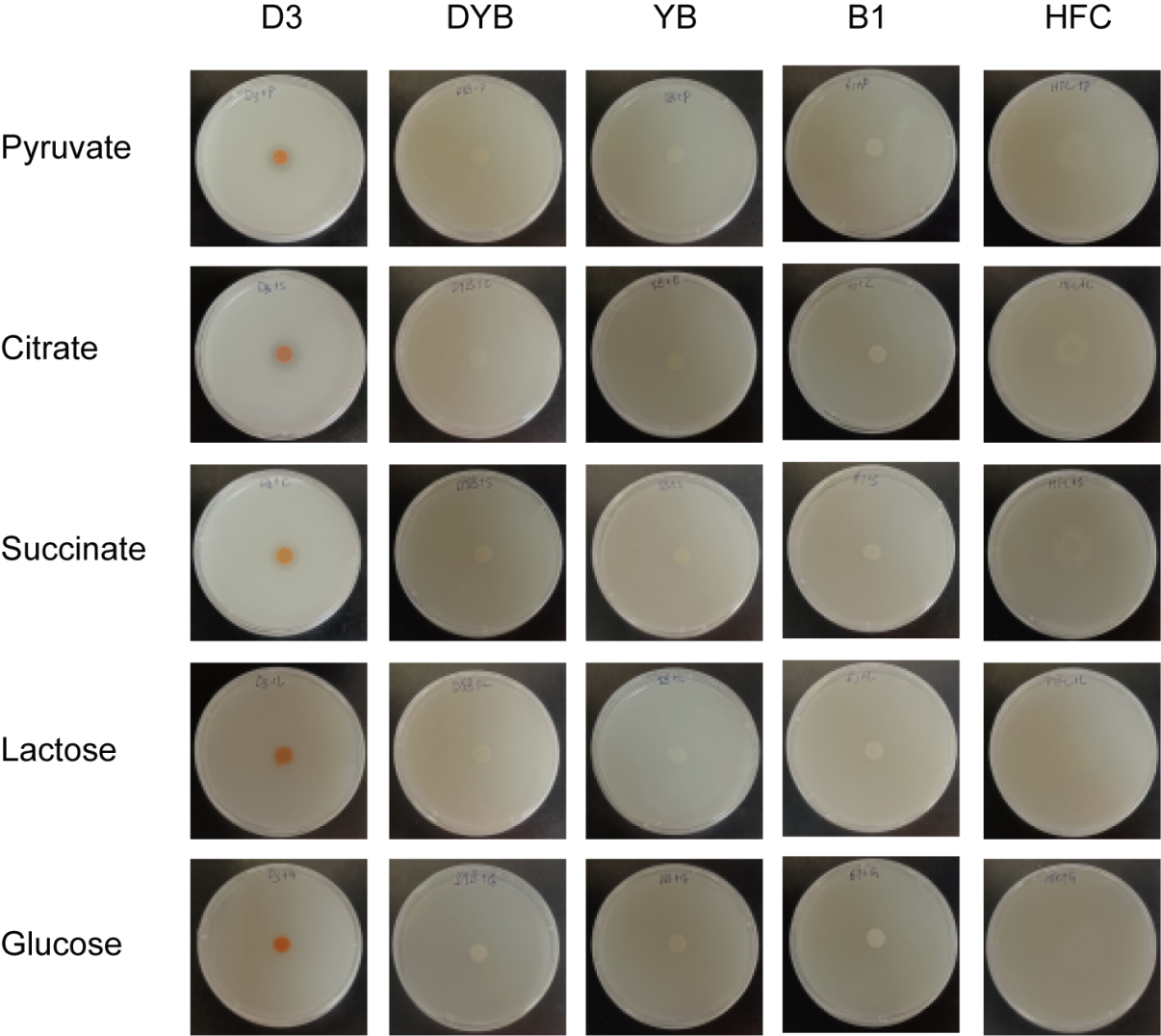


**Figure S2**. **Screening and identification of polyester-degrading yeast strains.** The strains were five yeast strains isolated from the gut of plastic-eating mealworm as shown in Table S1. Five different carbon sources were used for screening and optimization of yeast strains for degradation on PCL emulsion agar plates at 30 °C for 5–7 days.


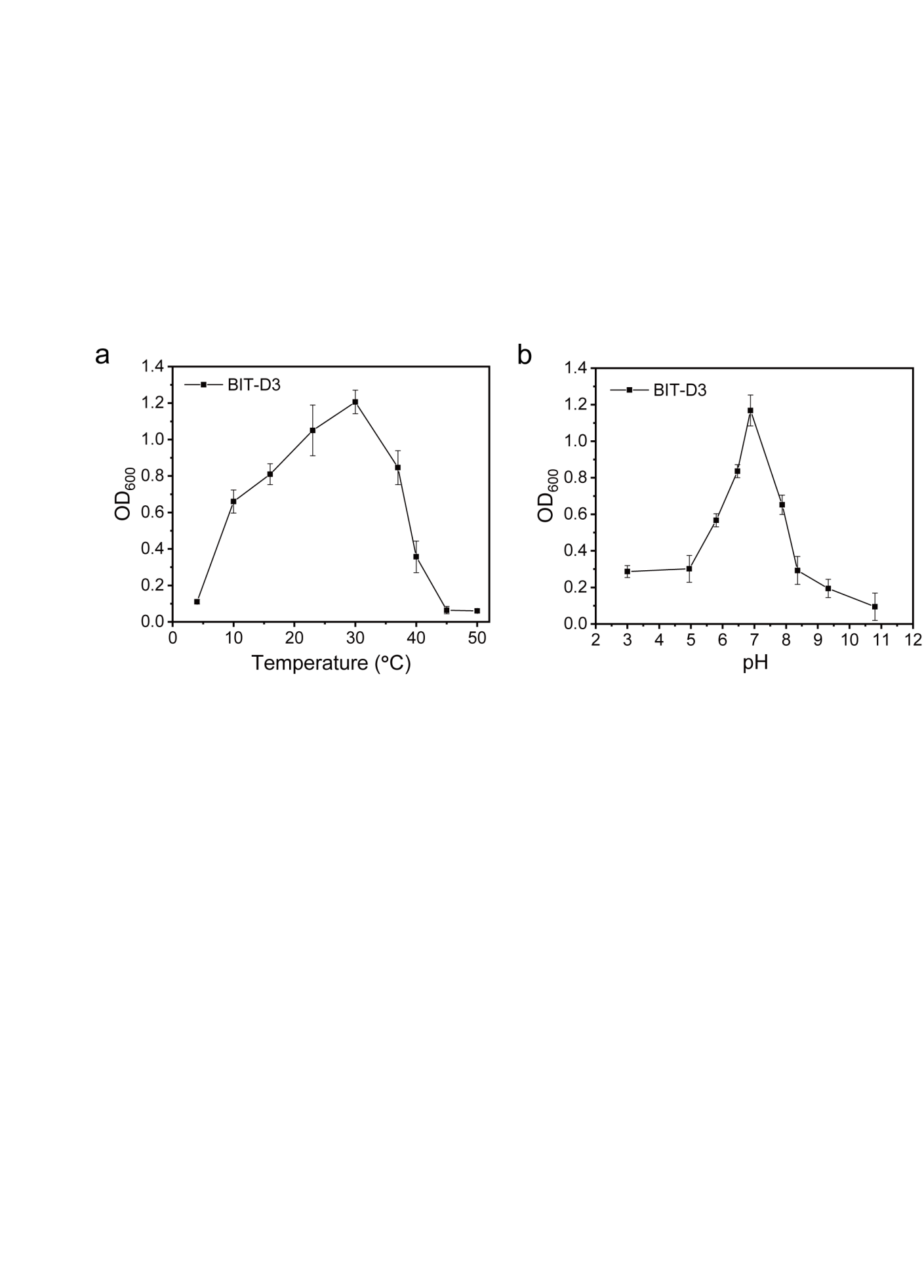


**Figure S3.** Optimum temperature (a) and pH (b) for the growth of the strain BIT-D3.


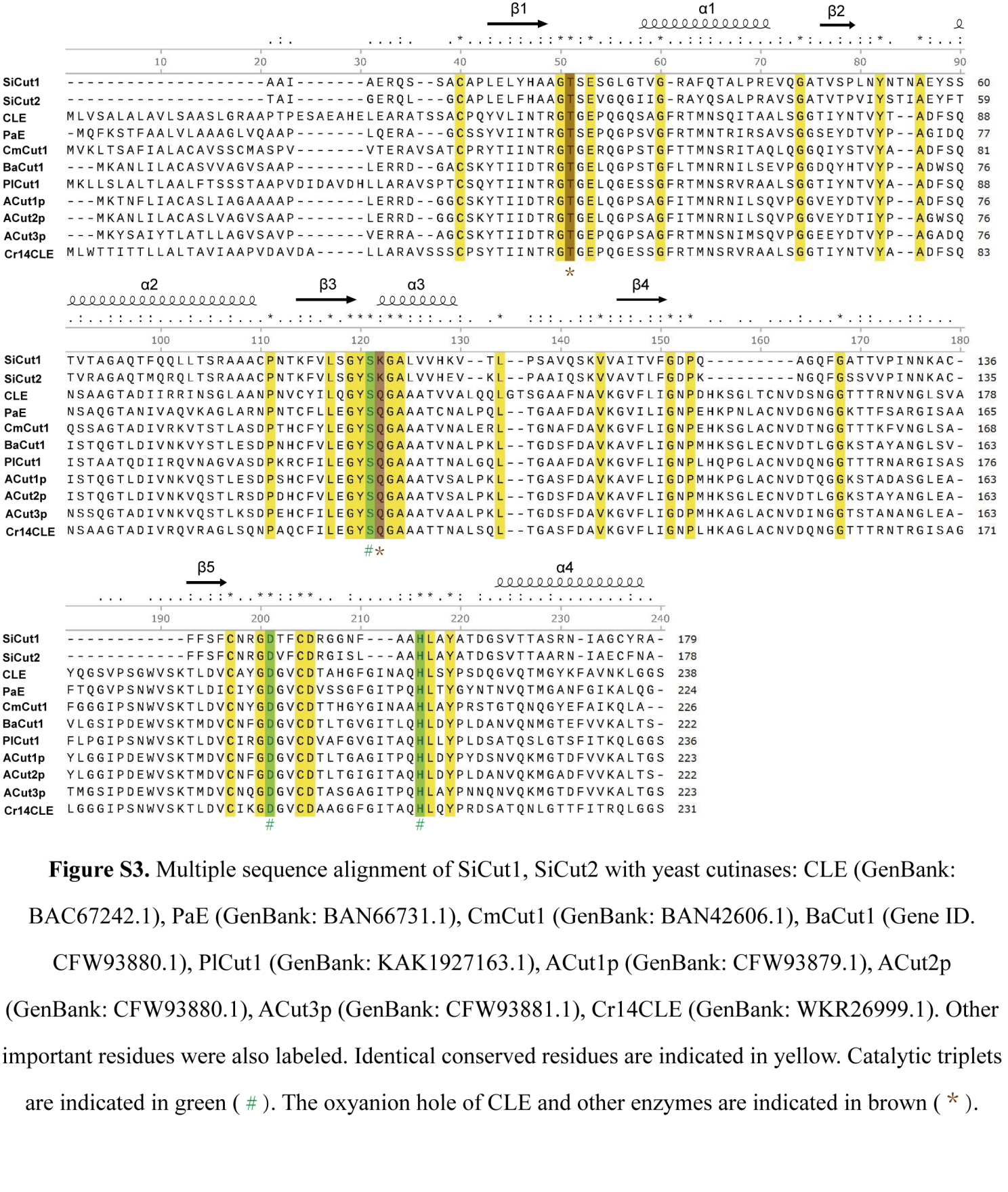


**Figure S4. Multiple sequence alignment of SiCut1, SiCut2 with yeast cutinases:** CLE (GenBank: BAC67242.1), PaE (GenBank: BAN66731.1), CmCut1 (GenBank: BAN42606.1), BaCut1 (Gene ID. CFW93880.1), PlCut1 (GenBank: KAK1927163.1), ACut1p (GenBank: CFW93879.1), ACut2p (GenBank: CFW93880.1), ACut3p (GenBank: CFW93881.1), Cr14CLE (GenBank: WKR26999.1). Other important residues were also labeled. Identical conserved residues are indicated in yellow. Catalytic triplets are indicated in green (#). The oxyanion hole of CLE and other enzymes are indicated in brown (*).


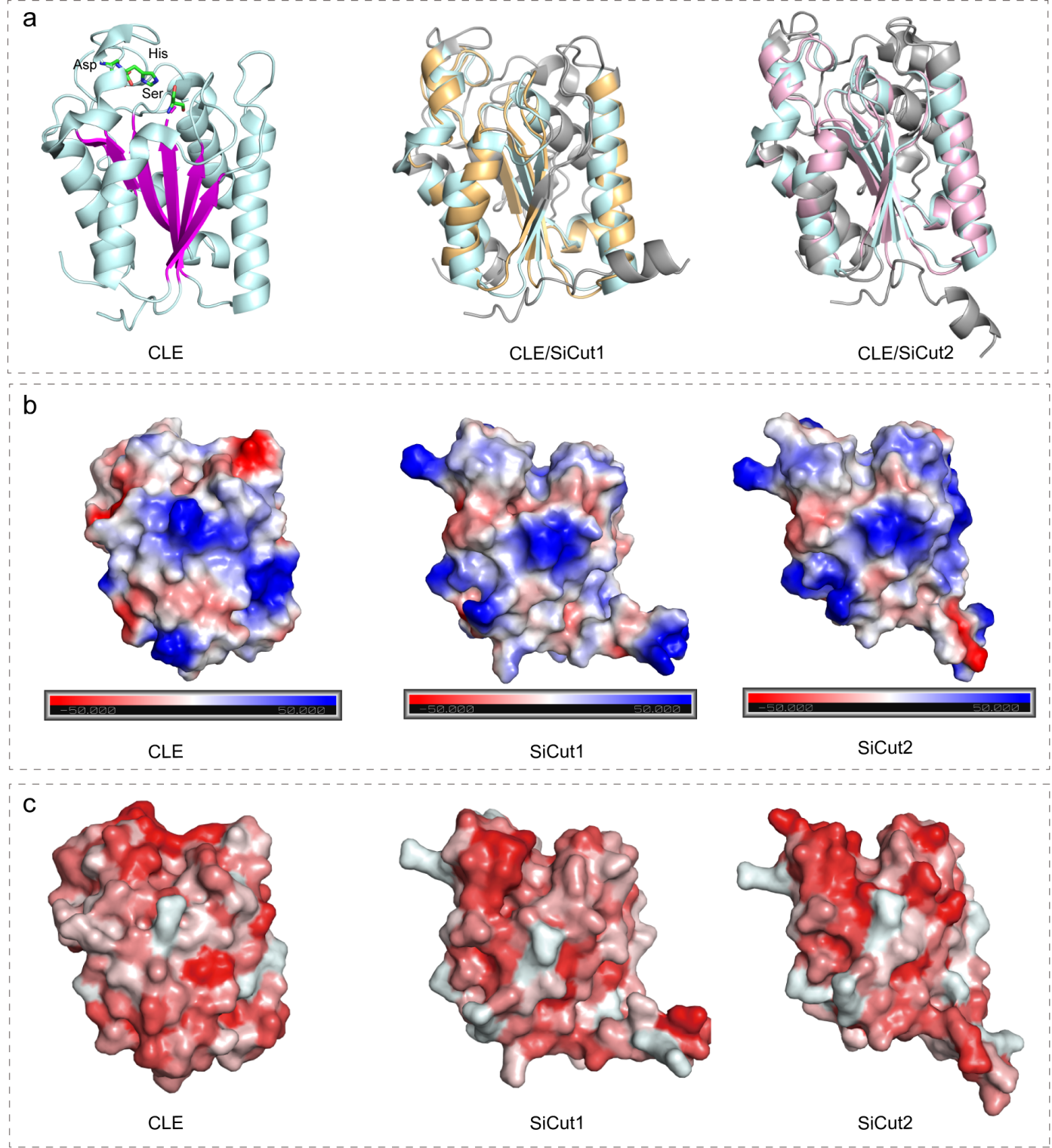


**Figure S5. Structural characterization of SiCut1 and SiCut2.** **(a)** Structure of cutinse-like enzyme CLE (PDB code:2CZQ) and the structural comparison of SiCut1 and SiCut2 with CLE. The catalytic triad (S85-D165-H180) is shown as green stick representations. Superposition of SiCut1(orange) and SiCut2(pink) with CLE (cyan). The overlapping regions are highlighted in gray color. **(b)** Electrostatic surface potential of CLE, SiCut1 and SiCut2 are colored in red and blue for the negative and positive charges respectively, and the white color represents the neutral residues. **(c)** Surface representation of CLE, SiCut1 and SiCut2 colored according to the hydrophobicity (hydrophobic, red; hydrophilic, white).


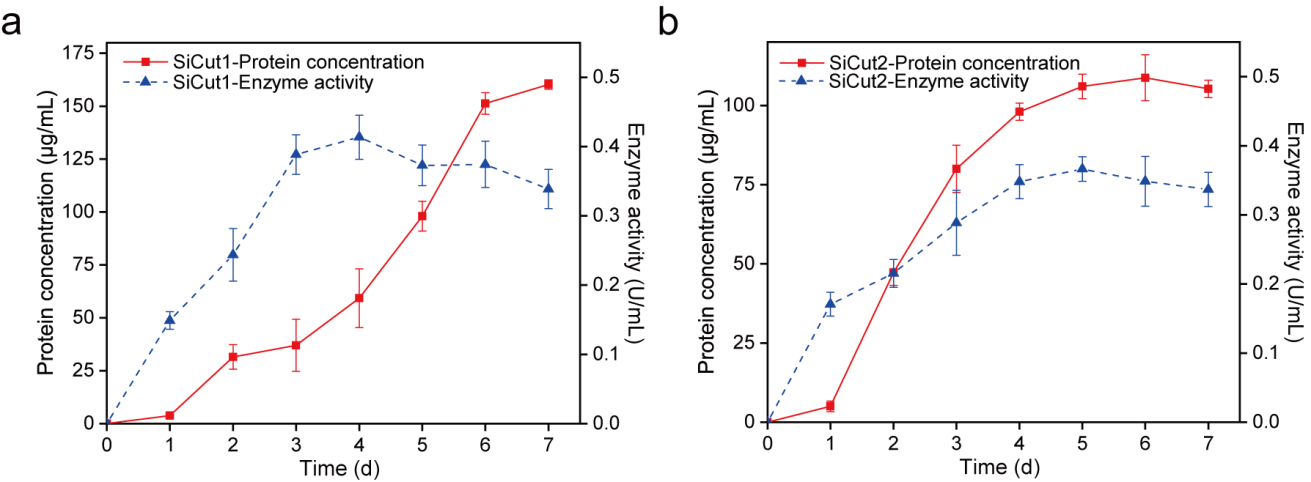


**Figure S6.** **Protein concentration and activity assays of (a) SiCut1 and (b) SiCut2 for each day of the 7 days fermentation.** The red line indicates protein concentration and the blue line indicates enzyme activity detected with *p*NP-butyrate (*p*NPB) as the substrate.


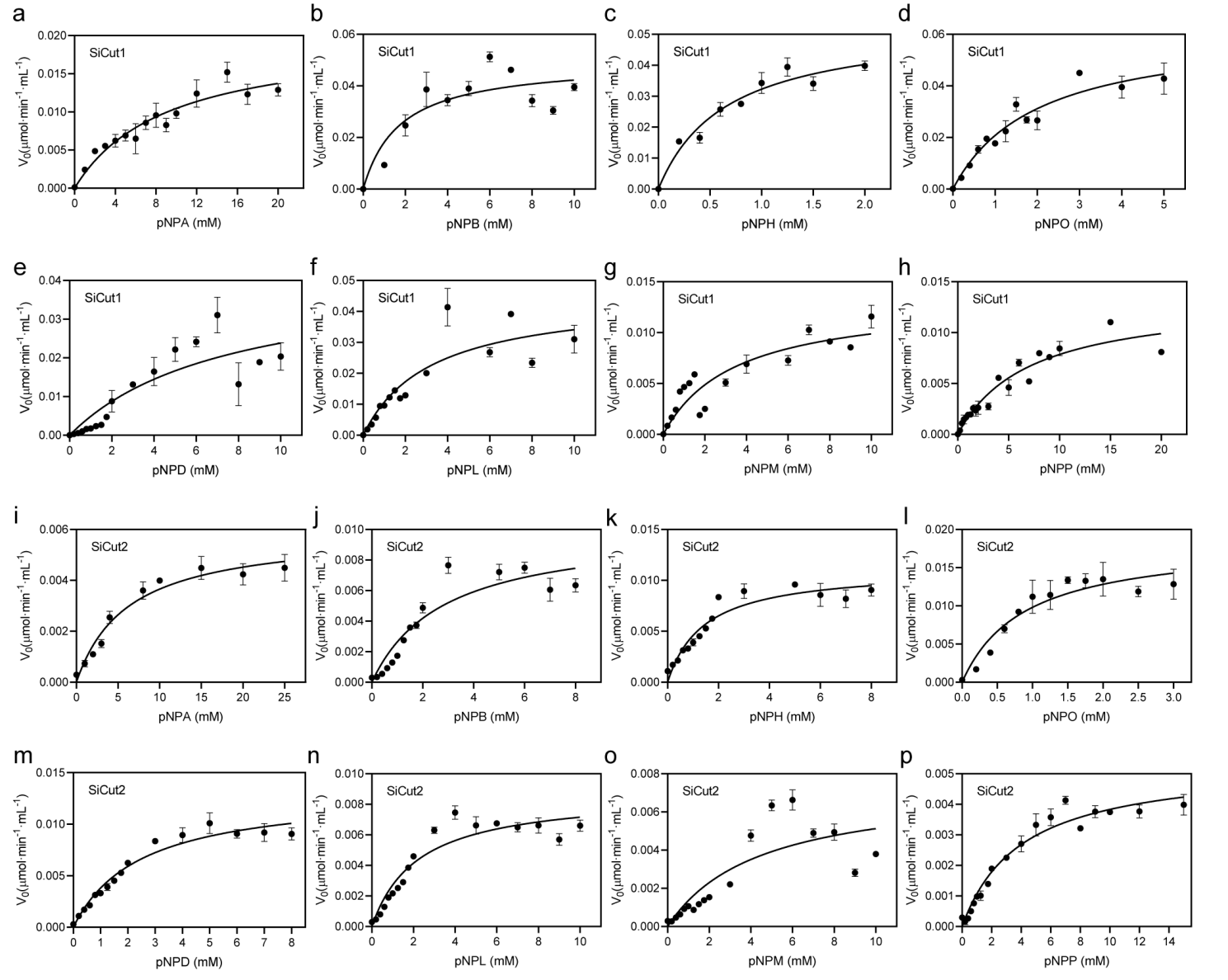


**Figure S7.** Michaelis-Menten (MM) fitting curves for the degradation of pNP esters by SiCut1 **(a-h)** and SiCut2 **(i-p)**.


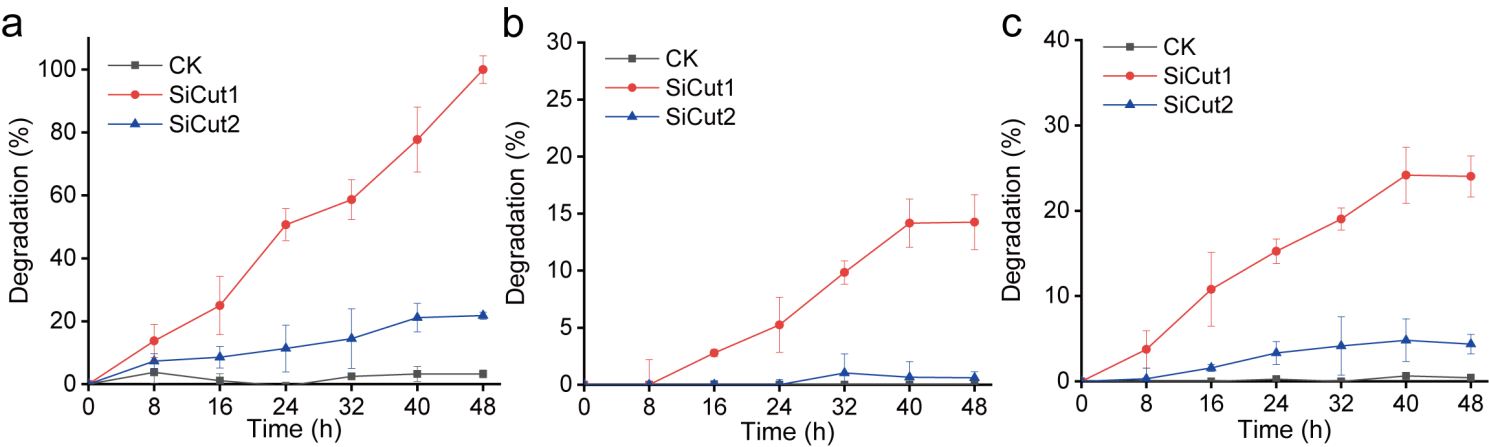


**Figure S8.** Degradation efficiency of degradation of PCL film **(a)**, PBS film **(b)** and polyester-PUR foam **(c)** by SiCut1 and SiCut2 over the course of 48 h.


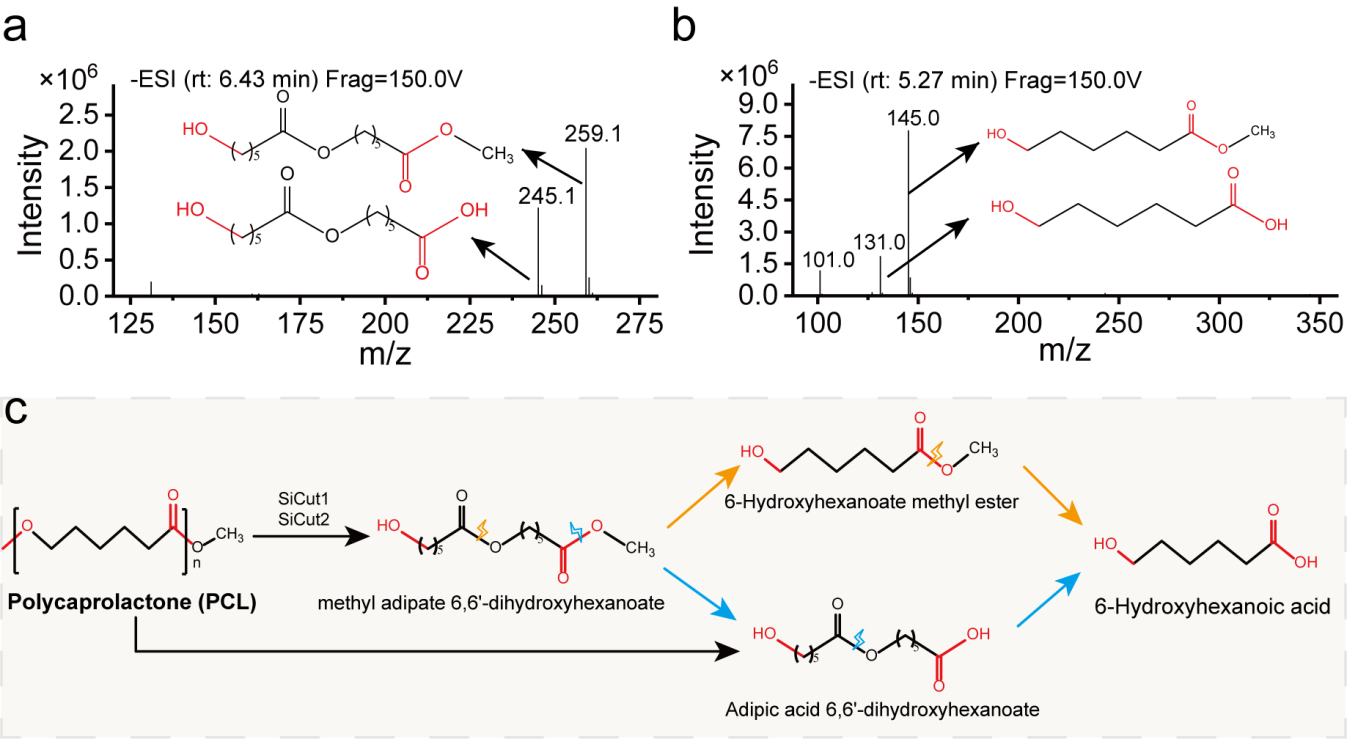


**Figure S9.** **Mass spectrometry analyses and proposed mechanism of the degradation of PCL film by SiCut1 and SiCut2.** **(a)** and **(b)** represent the mass spectra of PCL film degradation products corresponding to Fig. 5c. The peaks of the spectrum are marked with their m/z ratio. **(c)** The ester bond of polycaprolactone (PCL) is broken by SiCut1 and SiCut2 to form the PCL dimer (adipic acid 6,6'-dihydroxyhexanoate) and PCL subterminal dimer (methyl adipate 6,6'-dihydroxyhexanoate). And the ester bonds of these dimer are further broken to form the PCL monomer 6-hydroxyhexanoic acid or the PCL terminal monomer 6-hydroxyhexanoate methyl ester.


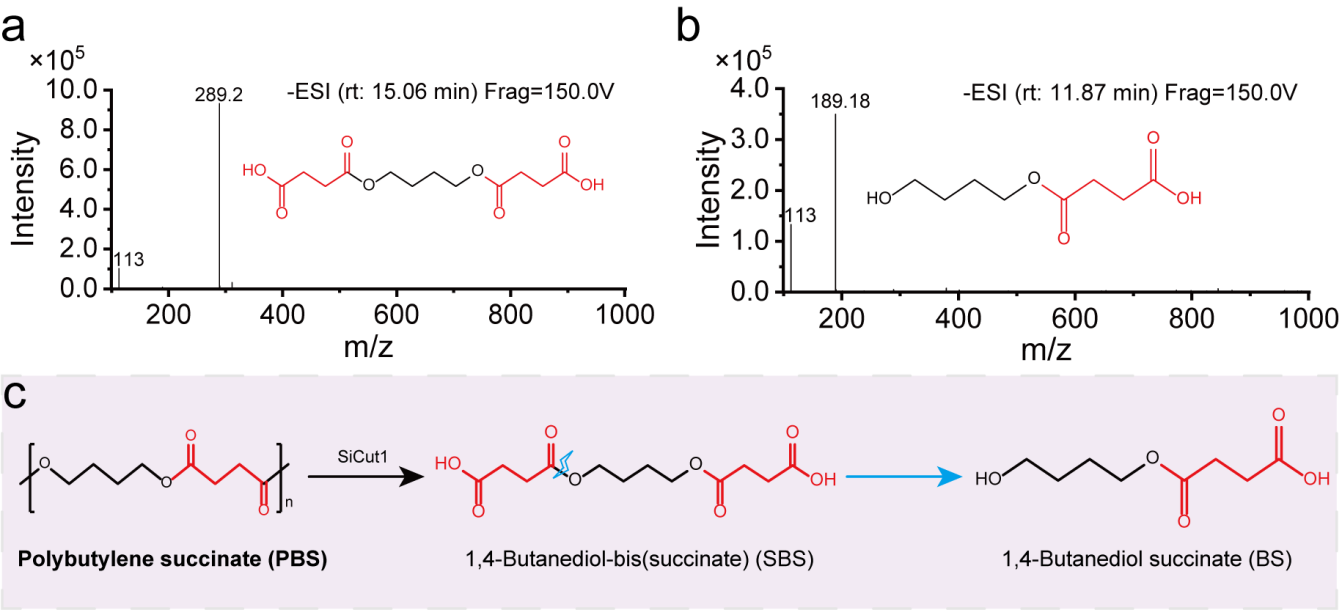


**Figure S10. Mass spectrometry analyses and proposed mechanism of the degradation of PBS film by SiCut1.** **(a)** and **(b)** represent mass spectra of PBS film degradation products corresponding to Fig 5d. The peaks of the spectrum are marked with their m/z ratio. **(c)** The ester bond of polybutylene succinate (PBS) is broken by SiCut1 to form the PBS trimer 1,4-butanediol-bis(succinate) (SBS), which is further degraded to the PBS dimer 1,4-butanediol succinate (BS).


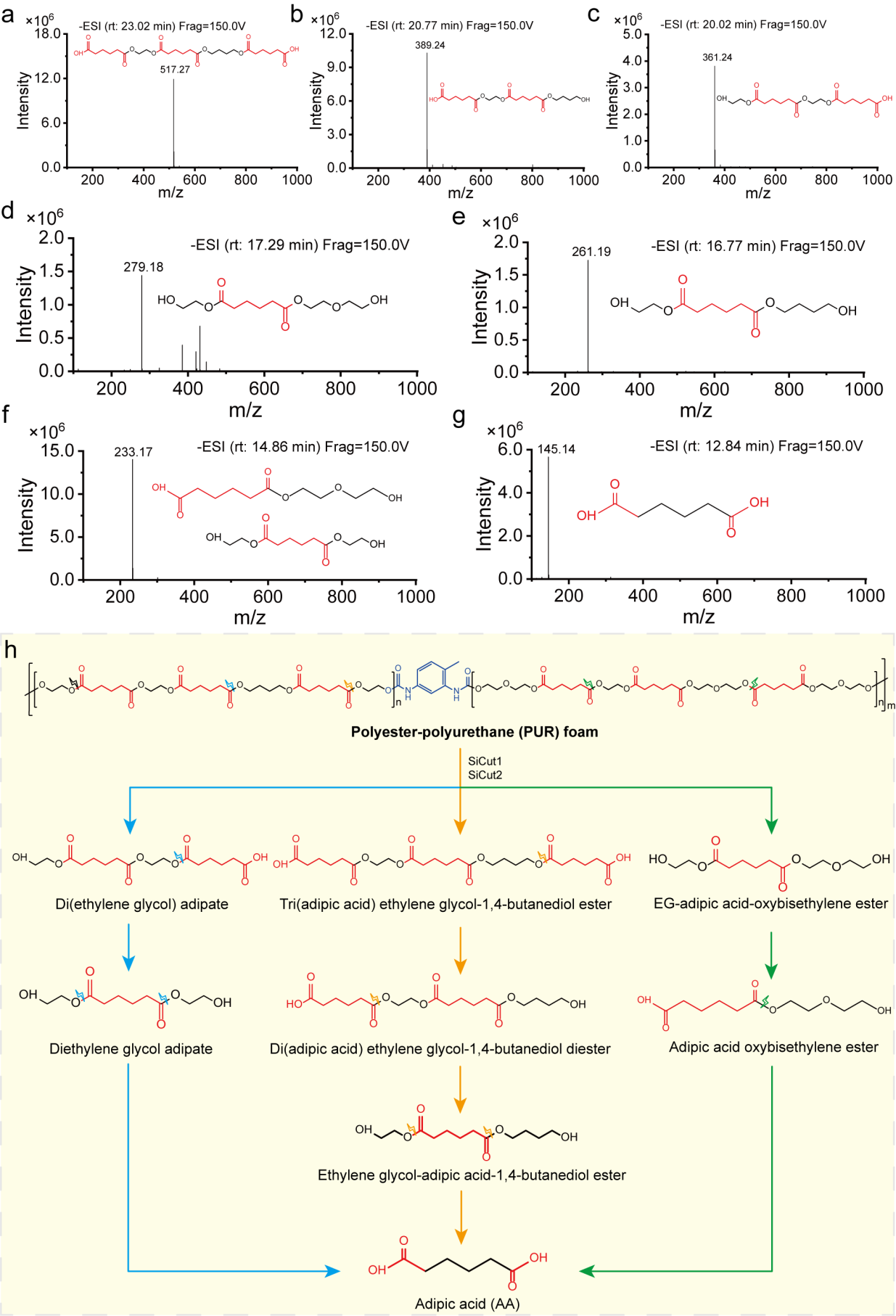


**Figure S11. Mass spectrometry analyses and proposed mechanism of the degradation of polyester-PUR foam by SiCut1 and SiCut2. (a)-(g)** represent mass spectra of polyester-PUR foam degradation products corresponding to Fig. 5g. The peaks of the spectrum are marked with their m/z ratio. **(h)** The ester bond of polyester-polyurethane (PUR) foam is broken by SiCut1 and SiCut2 to form the oligomer di(ethylene glycol) adipate, tri(adipic acid) ethylene glycol-1,4-butanediol ester, and ethylene glycol-adipic acid-oxybisethylene ester. Di(ethylene glycol) adipate can be further degraded to diethylene glycol adipate. Tri(adipic acid) ethylene glycol-1,4-butanediol ester can be gradually degraded to di(adipic acid) ethylene glycol-1,4-butanediol diester and ethylene glycol-adipic acid-1,4-butanediol ester with the gradual breaking of the ester bond. Ethylene glycol-adipic acid-oxybisethylene ester was further degraded into adipic acid oxybisethylene ester. Adipic acid (AA) can be formed by the degradation of diethylene glycol adipate, ethylene glycol-adipic acid-1,4-butanediol ester, and adipic acid oxybisethylene ester.


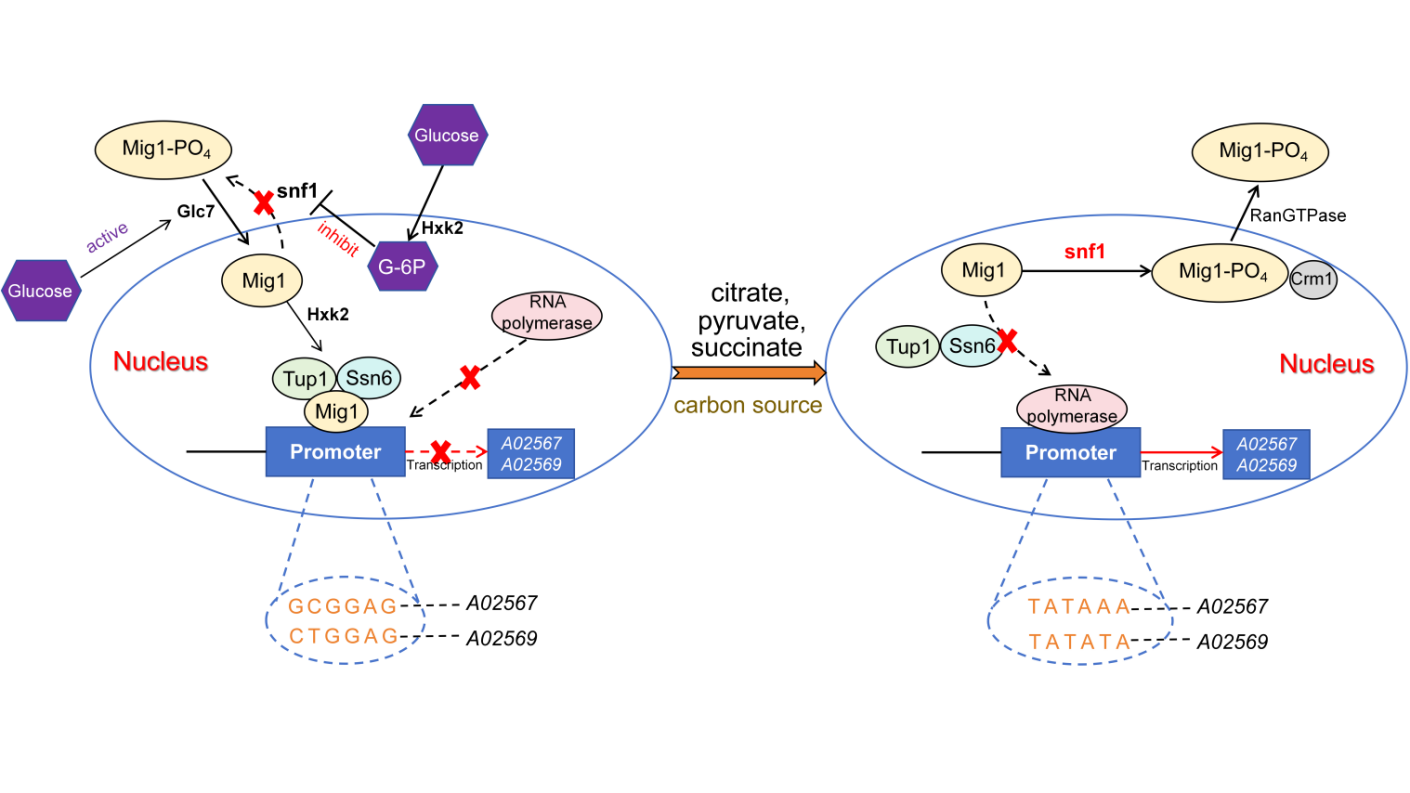


**Figure S12. Carbon catabolite repression (CCR) regulation mechanism in yeast.** The genes *A02567* and *A02569* are responsible for encoding the expression of cutinases SiCut1 and SiCut2, respectively. Mig1 (sequence motif: SYGGRG) is the primary regulator of CCR implementation in yeast. Hexokinase (Hxk2) has been demonstrated to facilitate the translocation of Mig1 to the nucleus, thereby influencing the regulation of Mig1 localization. The Snf1 protein kinase is capable of phosphorylating Mig1 in the presence of low glucose levels, which allows it to exit the nucleus and, as a result, derepress Mig1 from target gene transcription. In the presence of elevated glucose levels, Mig1 undergoes dephosphorylation and nuclear translocation, where it recruits the co-repressor proteins Tup1 and Ssn6 to impede transcription of the *A02567* and *A02569* genes by binding directly to their promoters. The inability of RNA polymerases to bind to the promoters of the *A02567* and *A02569* genes results in the cessation of transcription. The use of alternative substrates (citrate, pyruvate, succinate) as carbon sources results in Snf1-mediated phosphorylation of Mig1, which in turn leads to the release of its nuclear export signal (NES). The nuclear export protein Crm1 recognizes the phosphorylated NES sequence of Mig1 and binds to it to form a complex, which is then removed from the nucleus in dependence on the RanGTPase system. This process resumes transcription by inhibiting the action of Mig1. The RNA polymerase binds to the TATA box (sequence motif: TATA(A/T)A(A/T)) of the promoter, thereby initiating the transcription of the *A02567* and *A02569* genes.

**Table S1**. The material parameters of different polyester films.

| Parameters | PCL | PLA | PBS | PUR | PBAT | PET |
| --- | --- | --- | --- | --- | --- | --- |
| $\overline{M_{n}}$ | 12,820 | 45,180 | 32,190 | 99,190 | 35,380 | 25,210 |
| $\overline{M_{w}}$ | 33,790 | 115,330 | 86,740 | 194,020 | 82,970 | 40,930 |
| Crystallinity (%) | 71.0 | 32.7 | 38.6 | 37.5 | 6.6 | 4.5 |
| Glass transition temperature (°C) | -61 | 52 | 35 | -51 | -36 | 72 |
| Melting temperature (℃) | 65 | 164 | 113 | 148 | 126 | 248 |

**Table S2**. Isolation and characterization of insect intestinal yeasts.

| Yeasts | Most similar strains | Accession numbers | ITS rRNA/% |
| --- | --- | --- | --- |
| BIT-D3 | *Sakaguchia dacryoidea* CBS: 6353^T^ | KY105305.1 | 92.11 |
| BIT-B1 | *Debaryomyces robertsiae* CBS: 2934 | NR_138162.1 | 99.13 |
| BIT-HFC | *Hyphopichia burtonii* CBS: 2352 | KY107882.1 | 99.24 |
| BIT-YB | *Rugopelliculosa*sp. CBS: 6377 | KY106729.1 | 92.08 |
| BIT-DYB | *Saturnispora silvae*  CBS: 5498 | KY109554.1 | 100 |

**Table S3.** Annotation of 908 significant up-regulated secreted protein genes expressed in BIT-D3 based on transcriptome sequencing. -Log10 (p value)＞1.301 (p＜0.05) and Log2 (Fold Change)＞1.

| **Gene ID** | **Gene length** | **Log_2_ (Fold Change)** | **-Log_10_ (*p* value)** | **Gene annotation** |
| --- | --- | --- | --- | --- |
| A00007 | 1365 | 2.367 | 5.925 | FK506-binding protein 4 |
| A00025 | 711 | 1.098 | 1.378 | Archaeal histone |
| A00055 | 1920 | 4.954 | 20.389 | Phenylalanine/tyrosine ammonia-lyase |
| A00056 | 3516 | 1.085 | 1.429 | Cysteine dioxygenase |
| A00071 | 339 | 2.993 | 7.912 | Nucleolar protein 56 |
| A00073 | 1068 | 1.323 | 2.008 | Major Facilitator Superfamily |
| A00100 | 228 | 1.794 | 3.485 | C2H2-type domain-containing protein |
| A00101 | 1236 | 1.804 | 3.576 | Proteophosphoglycan ppg4 |
| A00120 | 729 | 1.697 | 3.279 | Universal stress protein |
| A00122 | 930 | 1.166 | 1.443 | ABC transporter permease |
| A00123 | 1140 | 1.543 | 2.323 | Parafibromin-like protein |
| A00140 | 1254 | 5.427 | 24.242 | Aquaglycerol porin |
| A00146 | 1044 | 1.623 | 2.678 | Putative oxidoreductase bli-4 |
| A00152 | 948 | 1.892 | 3.822 | Ferritin-like domain |
| A00154 | 1497 | 1.076 | 1.431 | Cytochrome b2, mitochondrial |
| A00170 | 465 | 1.072 | 1.430 | Superoxide dismutase |
| A00188 | 1059 | 1.517 | 2.569 | Diphthine methyl ester synthase |
| A00194 | 1077 | 1.096 | 1.470 | PAP2 superfamily |
| A00199 | 642 | 2.685 | 4.893 | N-terminal domain-containing protein |
| A00230 | 504 | 2.598 | 5.175 | Protein of unknown function (DUF2439) |
| A00240 | 1560 | 1.724 | 3.157 | DUF4200 domain-containing protein |
| A00253 | 906 | 1.927 | 1.308 | Serine/threonine-protein kinase haspin homolog hrk1 |
| A00294 | 996 | 2.627 | 5.133 | F-box domain-containing protein |
| A00326 | 750 | 4.694 | 11.570 | SRR1 |
| A00327 | 822 | 4.717 | 11.694 | Psi-producing oxygenase A |
| A00340 | 786 | 2.039 | 4.478 | Ribosome biogenesis protein NSA2 |
| A00365 | 1137 | 1.195 | 1.642 | Peroxidase |
| A00385 | 2547 | 2.572 | 6.835 | Probable U3 small nucleolar RNA-associated protein 13 |
| A00405 | 1164 | 2.642 | 7.038 | Ribosome biogenesis protein BRX1 |
| A00411 | 1341 | 2.666 | 7.164 | Nop53 (60S ribosomal biogenesis) |
| A00428 | 477 | 1.127 | 1.376 | Short-chain dehydrogenase/reductase SAT3 |
| A00440 | 468 | 4.450 | 5.395 | Short-chain dehydrogenase |
| A00443 | 2226 | 2.504 | 5.899 | Cocaine esterase |
| A00443 | 2226 | 2.504 | 5.899 | Cocaine esterase |
| A00447 | 1221 | 1.450 | 1.834 | Serine/threonine protein kinase |
| A00452 | 1593 | 5.218 | 22.846 | Cytochrome P450 monooxygenase |
| A00453 | 408 | 2.006 | 3.985 | Heat shock protein 9/12 |
| A00470 | 270 | 4.843 | 13.455 | Chromosome segregation ATPase |
| A00490 | 1194 | 1.323 | 2.073 | BZIP domain-containing protein |
| A00505 | 921 | 2.583 | 6.849 | Pre-rRNA-processing protein PNO1 |
| A00521 | 357 | 2.496 | 3.037 | Glycosyltransferase 2-like domain-containing protein |
| A00544 | 945 | 1.975 | 2.259 | DUF6533 domain-containing protein |
| A00545 | 963 | 1.662 | 2.031 | BTB domain-containing protein |
| A00550 | 954 | 1.090 | 1.357 | Putative aryl-alcohol dehydrogenase C977 |
| A00555 | 972 | 1.178 | 1.693 | Succinate/fumarate mitochondrial transporter |
| A00600 | 378 | 2.608 | 2.926 | Heat shock protein |
| A00601 | 1572 | 3.019 | 9.250 | Cytochrome P450 monooxygenase |
| A00607 | 1875 | 2.841 | 6.575 | PIG Liver carboxylesterase |
| A00619 | 576 | 2.215 | 3.850 | Glucose and ribitol dehydrogenase |
| A00622 | 1533 | 2.604 | 3.514 | MYND-type domain-containing protein |
| A00623 | 1437 | 2.223 | 1.375 | Lipoprotein |
| A00635 | 1341 | 3.399 | 10.675 | Nop53 (60S ribosomal biogenesis) |
| A00640 | 1185 | 3.316 | 10.218 | Ribosome biogenesis protein BRX1 |
| A00659 | 2478 | 3.486 | 11.450 | Transducin beta-like protein 3 |
| A00661 | 1659 | 2.765 | 5.564 | SGNH hydrolase-type esterase domain-containing protein |
| A00675 | 255 | 1.344 | 2.023 | SGNH hydrolase-type esterase domain-containing protein |
| A00688 | 882 | 1.424 | 1.992 | Domain of unknown function (DUF2431) |
| A00697 | 726 | 2.554 | 6.741 | Ribosome biogenesis protein NSA2 |
| A00705 | 1701 | 4.246 | 12.864 | Efflux pump FUB11 |
| A00717 | 648 | 1.987 | 4.056 | Homeobox protein HD-10 |
| A00735 | 438 | 1.516 | 2.652 | 40S ribosomal protein S23 |
| A00742 | 381 | 2.377 | 1.306 | Ion transport domain-containing protein |
| A00762 | 1584 | 1.526 | 2.283 | Arrestin-related trafficking adapter 10 |
| A00774 | 279 | 2.175 | 3.572 | DASH complex subunit DAD1 |
| A00776 | 1560 | 2.335 | 4.857 | DUF4200 domain-containing protein |
| A00793 | 1395 | 2.140 | 4.941 | Amino acid transport system protein |
| A00831 | 1245 | 4.367 | 17.345 | Aquaglycerol porin AQY3 |
| A00841 | 948 | 4.644 | 16.419 | Ferritin-like domain |
| A00871 | 1059 | 1.355 | 2.123 | Diphthine methyl ester synthase |
| A00883 | 1008 | 1.234 | 1.741 | Serine/threonine-protein phosphatase 5 |
| A00884 | 780 | 1.288 | 1.873 | Serine/threonine-protein phosphatase T |
| A00900 | 501 | 1.718 | 2.903 | Peptidylprolyl isomerase |
| A00904 | 945 | 1.132 | 1.532 | Glutathione S-transferase LANCL1 |
| A00916 | 1194 | 2.881 | 3.694 | Adenosine 3'-phospho 5'-phosphosulfate transporter 1 |
| A00921 | 333 | 1.810 | 3.328 | Nucleolar protein 56 |
| A00934 | 642 | 1.862 | 3.620 | Cysteine dioxygenase |
| A00936 | 1896 | 3.805 | 13.700 | Phenylalanine/tyrosine ammonia-lyase |
| A00950 | 1818 | 1.068 | 1.351 | Neutrophil cytosol factor 2 |
| A00982 | 1362 | 2.932 | 8.671 | FK506-binding protein 4 |
| A01011 | 1680 | 1.758 | 3.414 | Probable DNA-directed RNA polymerase III subunit rpc6 |
| A01016 | 918 | 2.654 | 7.116 | Pre-rRNA-processing protein PNO1 |
| A01030 | 909 | 1.939 | 3.936 | Zn(2)-C6 fungal-type domain-containing protein |
| A01041 | 417 | 2.623 | 1.687 | HOOK N-terminal domain-containing protein |
| A01042 | 435 | 3.326 | 3.153 | Scaffolding protein |
| A01063 | 789 | 1.056 | 1.357 | Kinase-regulated stress-responsive transcription factor skn7 |
| A01086 | 774 | 1.271 | 1.943 | 40S ribosomal protein S1 |
| A01109 | 417 | 1.849 | 3.232 | Uncharacterized protein C3B9.05 |
| A01116 | 537 | 1.100 | 1.362 | Ribosomal protein L19, mitochondrial |
| A01127 | 1554 | 1.893 | 3.894 | Protein SOF1 |
| A01135 | 393 | 1.063 | 1.406 | 60S ribosomal protein L35-4 |
| A01142 | 327 | 1.324 | 2.087 | 40S ribosomal protein S25-A |
| A01154 | 1251 | 1.098 | 1.435 | Centromere protein Scm3 |
| A01159 | 603 | 1.192 | 1.697 | BTB domain-containing protein |
| A01174 | 555 | 3.669 | 8.181 | MYND-type domain-containing protein |
| A01177 | 534 | 1.341 | 1.873 | Lipoprotein |
| A01207 | 387 | 2.059 | 4.617 | 60S ribosomal protein L33-B |
| A01243 | 1290 | 2.336 | 4.528 | Uncharacterized transporter C460.05 |
| A01277 | 528 | 1.634 | 3.037 | 60S ribosomal protein L17 |
| A01280 | 1203 | 1.907 | 3.079 | SGNH/GDSL hydrolase family protein |
| A01286 | 1479 | 2.082 | 3.750 | SGNH hydrolase-type esterase domain-containing protein |
| A01292 | 1119 | 1.152 | 1.586 | Exostosin-like 2 |
| A01298 | 1428 | 2.648 | 5.725 | SGNH hydrolase-type esterase domain-containing protein |
| A01325 | 1479 | 2.034 | 4.020 | SGNH hydrolase-type esterase domain-containing protein |
| A01374 | 1176 | 3.060 | 8.779 | Probable RNA 3'-terminal phosphate cyclase-like protein |
| A01385 | 606 | 3.751 | 13.309 | Zinc knuckle |
| A01393 | 942 | 4.876 | 8.691 | Protein FIZZY-RELATED 3 |
| A01419 | 738 | 1.223 | 1.774 | Eukaryotic translation initiation factor 6 |
| A01425 | 1605 | 1.485 | 2.519 | Purine-cytosine permease fcyB |
| A01429 | 1590 | 1.580 | 2.768 | ATP-dependent rRNA helicase RRP3 |
| A01457 | 441 | 1.119 | 1.517 | Pre-mRNA-processing factor 19 |
| A01499 | 969 | 1.626 | 3.014 | rRNA 2'-O-methyltransferase fibrillarin |
| A01505 | 426 | 2.114 | 4.745 | Proteophosphoglycan ppg4 |
| A01511 | 1803 | 1.287 | 1.992 | Sugar transport protein 9 |
| A01520 | 609 | 1.772 | 3.523 | 60S ribosomal protein L6-2 |
| A01531 | 888 | 1.507 | 2.525 | ATP-dependent RNA helicase |
| A01539 | 1545 | 1.097 | 1.307 | Monopolin complex subunit Csm1/Pcs1 C-terminal domain-containing protein |
| A01582 | 1368 | 1.346 | 1.953 | SET domain |
| A01586 | 1581 | 2.555 | 5.426 | Guanine deaminase |
| A01647 | 1809 | 1.178 | 1.546 | G2/M phase checkpoint control protein Sum2 |
| A01662 | 804 | 1.369 | 1.959 | SGNH hydrolase-type esterase domain-containing protein |
| A01688 | 2955 | 1.554 | 2.668 | Para-nitrobenzyl esterase |
| A01701 | 1284 | 2.464 | 5.569 | Carbohydrate esterase family 16 protein |
| A01744 | 498 | 1.445 | 2.448 | 60S ribosomal protein L12-A |
| A01771 | 645 | 3.003 | 8.373 | Ribosome biogenesis regulatory protein homolog |
| A01804 | 1374 | 1.777 | 3.490 | C2H2-type domain-containing protein |
| A01816 | 1692 | 1.601 | 2.877 | L-tyrosine:2-oxoglutarate aminotransferase amt1 |
| A01817 | 351 | 1.757 | 3.452 | 60S ribosomal protein L34-A |
| A01828 | 2058 | 2.922 | 8.278 | Proteophosphoglycan ppg4 |
| A01838 | 1233 | 6.345 | 1.738 | Replication protein, putative |
| A01862 | 237 | 3.315 | 1.333 | Plasma membrane proteolipid 3 |
| A01863 | 2898 | 2.197 | 5.154 | Iron-containing redox enzyme |
| A01866 | 708 | 3.776 | 5.258 | Plasma membrane proteolipid 3 |
| A01882 | 1044 | 1.223 | 1.564 | Methyltransferase domain |
| A01891 | 537 | 1.527 | 2.322 | Centrin-3 |
| A01952 | 1362 | 2.313 | 5.744 | Delta(12) fatty acid desaturase |
| A01958 | 1431 | 1.044 | 1.356 | 5-hydroxyisourate hydrolase |
| A01971 | 1605 | 1.354 | 2.180 | Major Facilitator Superfamily |
| A01979 | 303 | 1.968 | 3.737 | Mitochondrial import inner membrane translocase subunit tim10 |
| A01996 | 318 | 1.156 | 1.637 | 60S ribosomal protein L36-A |
| A02038 | 1494 | 2.061 | 3.333 | Serine/threonine-protein kinase ark1 |
| A02051 | 597 | 1.623 | 2.627 | protein disulfide-isomerase |
| A02077 | 1149 | 3.209 | 7.831 | Kinetochore protein Sos7 coiled-coil domain-containing protein |
| A02085 | 852 | 1.974 | 3.338 | G2/M phase checkpoint control protein Sum2 |
| A02102 | 789 | 2.166 | 4.731 | Dehydrodolichyl diphosphate synthase complex subunit SPAC4D7.04c |
| A02103 | 318 | 1.410 | 1.994 | PET assembly of cytochrome c oxidase, mitochondrial |
| A02107 | 456 | 1.153 | 1.633 | 40S ribosomal protein S13-1 |
| A02110 | 1086 | 2.361 | 5.150 | Protein PGR |
| A02145 | 1260 | 1.136 | 1.357 | Proteophosphoglycan ppg4 |
| A02184 | 1323 | 1.780 | 2.469 | Probable kinetochore protein NUF2 |
| A02209 | 2016 | 1.270 | 1.843 | Carbohydrate esterase family 16 protein |
| A02214 | 1833 | 2.087 | 4.752 | Solute carrier family 28 member 3 |
| A02219 | 1767 | 1.361 | 2.087 | FAD-binding monooxygenase moxY |
| A02225 | 1482 | 1.174 | 1.381 | CN hydrolase domain-containing protein |
| A02231 | 891 | 2.340 | 3.511 | RecA family profile 1 domain-containing protein |
| A02232 | 639 | 1.508 | 1.922 | Uncharacterized oxidoreductase SSP0419 |
| A02299 | 1197 | 1.349 | 1.372 | REJ domain-containing protein |
| A02309 | 441 | 1.170 | 1.633 | 17.9 kDa class I heat shock protein |
| A02327 | 624 | 1.709 | 3.197 | LURP-one-related |
| A02354 | 408 | 4.617 | 16.634 | Histone H3.2 |
| A02362 | 1059 | 3.140 | 3.280 | Serine/threonine-protein kinase mph1 |
| A02375 | 618 | 2.331 | 5.514 | Glutathione-dependent formaldehyde-activating enzyme |
| A02385 | 1956 | 2.328 | 5.600 | Uncharacterized protein C1604.06c |
| A02388 | 693 | 1.101 | 1.318 | Stress response protein NST1 |
| A02392 | 408 | 4.139 | 12.111 | Histone H3.2 |
| A02395 | 678 | 2.803 | 6.500 | Carboxylesterase type B domain-containing protein |
| A02407 | 1377 | 2.366 | 5.953 | Peptidase M20 domain-containing protein 2 |
| A02414 | 546 | 1.711 | 3.304 | 60S ribosomal protein L28-A |
| A02417 | 435 | 1.171 | 1.575 | Mitochondrial ribosomal protein L27 |
| A02428 | 1119 | 3.014 | 3.397 | Exodeoxyribonuclease 1 |
| A02434 | 615 | 4.408 | 13.527 | DDE Tnp4 domain-containing protein |
| A02456 | 2157 | 1.632 | 2.476 | Superoxide-generating NADPH oxidase heavy chain subunit A |
| A02477 | 846 | 3.248 | 7.718 | Glycosyl hydrolases family 16 |
| A02533 | 1452 | 3.157 | 4.591 | Ubiquitin-conjugating enzyme E2 S |
| A02535 | 1581 | 1.179 | 1.696 | Cytochrome P450 61 |
| A02538 | 810 | 2.130 | 4.255 | DNA topoisomerase 6 subunit A3 |
| A02541 | 1161 | 3.305 | 10.605 | Proteophosphoglycan ppg4 |
| A02542 | 3480 | 2.664 | 7.276 | Phosphate-repressible phosphate permease pho-4 |
| **A02567** | 600 | 8.577 | 2.975 | **Cutinase SiCut1** |
| A02568 | 597 | 1.914 | 3.256 | esterase |
| **A02569** | 597 | 16.732 | 4.580 | **Cutinase SiCut2** |
| A02572 | 285 | 1.707 | 3.292 | 40S ribosomal protein S27 |
| A02577 | 585 | 2.819 | 7.928 | tRNA N6-adenosine threonylcarbamoyltransferase |
| A02598 | 1713 | 1.511 | 2.653 | Phosphate-repressible phosphate permease pho-4 |
| A02608 | 426 | 1.501 | 1.386 | Methyltransferase small domain-containing protein |
| A02619 | 660 | 1.430 | 2.134 | Thioredoxin domain-containing protein plp1 |
| A02627 | 588 | 2.340 | 3.585 | Microtubule-associated protein Jupiter |
| A02628 | 987 | 1.152 | 1.551 | 54S ribosomal protein L4, mitochondrial |
| A02638 | 1953 | 1.980 | 2.994 | non-specific serine/threonine protein kinase |
| A02639 | 537 | 1.687 | 2.923 | Mediator of RNA polymerase II transcription subunit 10 |
| A02649 | 2115 | 2.530 | 6.653 | ATP-dependent RNA helicase HAS1 |
| A02657 | 2145 | 4.327 | 16.795 | Probable nucleolar GTP-binding protein 1 |
| A02661 | 1785 | 2.109 | 4.798 | Ribosome assembly factor MRT4 |
| A02710 | 2010 | 3.510 | 11.638 | Sulfatase |
| A02736 | 1692 | 1.308 | 2.030 | Probable sugar phosphate/phosphate translocator At5g11230 |
| A02747 | 1023 | 1.115 | 1.479 | Putative monooxygenase Rv1533 |
| A02771 | 1137 | 2.155 | 4.388 | Alcohol dehydrogenase 3 |
| A02793 | 2229 | 3.126 | 8.668 | Beta-glucan synthesis-associated protein KRE6 |
| A02795 | 705 | 1.704 | 1.933 | Cell division control protein 14, SIN component |
| A02818 | 309 | 1.610 | 2.371 | Exoribonuclease family, domain 1 |
| A02823 | 666 | 1.795 | 3.422 | Tox-PL domain-containing protein |
| A02829 | 444 | 2.465 | 4.735 | UDP-N-acetylglucosamine transferase subunit alg13 |
| A02838 | 840 | 2.742 | 7.736 | Anaphase-promoting complex subunit 4-like WD40 domain-containing protein |
| A02839 | 639 | 1.663 | 2.997 | Yeast cell wall synthesis Kre9/Knh1-like N-terminal domain-containing protein |
| A02855 | 663 | 1.416 | 1.804 | Cyanamide hydratase |
| A02866 | 1113 | 1.321 | 1.986 | Telomere length and silencing protein 1 homolog |
| A02874 | 1665 | 1.161 | 1.651 | Uncharacterized amino-acid permease C15C4.04c |
| A02903 | 3513 | 2.169 | 4.894 | U3 small nucleolar RNA-associated protein 25 |
| A02918 | 558 | 1.858 | 3.338 | rRNA processing/ribosome biogenesis |
| A02921 | 504 | 1.080 | 1.446 | Translationally-controlled tumor protein homolog |
| A02926 | 825 | 5.757 | 26.402 | Protein alcS |
| A02927 | 1887 | 3.221 | 10.432 | Probable indole-3-pyruvate monooxygenase YUCCA11 |
| A02928 | 1323 | 2.153 | 4.705 | Monocarboxylate transporter 12 |
| A02934 | 1077 | 1.223 | 1.772 | C2H2-type domain-containing protein |
| A02955 | 759 | 1.154 | 1.527 | FK506-binding protein 5 |
| A02964 | 921 | 2.612 | 2.398 | Uncharacterized transporter YrhG |
| A02969 | 2025 | 1.676 | 3.046 | Cryptococcal mannosyltransferase 1 |
| A02978 | 648 | 2.258 | 4.456 | EKC/KEOPS complex subunit CGI121 |
| A02979 | 471 | 1.676 | 3.177 | 40S ribosomal protein S11-A |
| A03029 | 1800 | 2.876 | 7.286 | Scytalone dehydratase-like protein Arp1 |
| A03031 | 588 | 4.172 | 10.471 | Protein kinase domain |
| A03037 | 2124 | 1.600 | 2.877 | Ubiquitin carboxyl-terminal hydrolase 36 |
| A03053 | 648 | 2.820 | 5.426 | F-box domain-containing protein |
| A03061 | 585 | 1.446 | 2.452 | 40S ribosomal protein S5 |
| A03072 | 1017 | 1.867 | 3.534 | Threonylcarbamoyl-AMP synthase |
| A03073 | 1335 | 1.802 | 3.442 | Proteophosphoglycan ppg4 |
| A03074 | 1728 | 2.354 | 5.744 | U3 small nucleolar RNA-associated protein 15 |
| A03078 | 831 | 2.151 | 4.748 | U3 small nucleolar ribonucleoprotein protein IMP4 |
| A03090 | 2445 | 1.779 | 3.225 | DNA replication licensing factor mcm10 |
| A03093 | 1083 | 1.695 | 1.985 | GDP-mannose-dependent alpha-mannosyltransferase |
| A03133 | 1302 | 5.929 | 14.259 | Uncharacterized transporter C11D3.18C |
| A03160 | 3276 | 2.354 | 5.913 | Probable importin subunit beta-4 |
| A03164 | 795 | 2.697 | 1.810 | DUF6534 domain-containing protein |
| A03180 | 279 | 1.171 | 1.663 | 60S ribosomal protein L38-1 |
| A03181 | 636 | 1.521 | 2.677 | 40S ribosomal protein S24 |
| A03182 | 1620 | 1.506 | 2.436 | UPF0187 protein sll1024 |
| A03235 | 1326 | 1.358 | 2.169 | Uncharacterized solute carrier family 35 member C320.08 |
| A03268 | 1371 | 1.209 | 1.578 | F-box-like |
| A03279 | 3552 | 1.169 | 1.614 | Uncharacterized ABC transporter ATP-binding protein/permease YOL075C |
| A03281 | 633 | 1.078 | 1.388 | Methyltransferase domain |
| A03288 | 333 | 1.531 | 2.390 | Dynein light chain 1, cytoplasmic |
| A03298 | 564 | 2.610 | 4.838 | Uncharacterized protein C6G9.01c |
| A03299 | 852 | 1.580 | 2.875 | 40S ribosomal protein S4-C |
| A03342 | 1530 | 1.485 | 2.282 | Cell division control protein 25 |
| A03344 | 519 | 4.654 | 13.469 | Tyrosine-protein kinase ephrin type A/B receptor-like domain-containing protein |
| A03345 | 291 | 1.692 | 2.994 | Leydig cell tumor 10 kDa protein homolog |
| A03355 | 1104 | 1.070 | 1.341 | Fungal specific transcription factor domain |
| A03374 | 1860 | 2.716 | 4.205 | Caffeine resistance protein 5 |
| A03375 | 690 | 2.223 | 4.055 | Mind kinetochore complex component Nnf1 |
| A03386 | 456 | 1.247 | 1.403 | Probable ribonuclease P/MRP protein subunit POP5 |
| A03393 | 288 | 1.507 | 2.631 | Probable 60S ribosomal protein L37-A |
| A03398 | 927 | 2.373 | 5.198 | Chromo shadow domain |
| A03402 | 1539 | 1.227 | 1.829 | Acyl-CoA desaturase |
| A03412 | 801 | 2.056 | 3.192 | Protein alan shepard |
| A03416 | 477 | 1.227 | 1.809 | 60S ribosomal protein L24-A |
| A03428 | 1467 | 2.477 | 3.869 | Anaphase spindle elongation protein 1 |
| A03432 | 309 | 1.763 | 2.889 | Mitochondrial export protein Som1 |
| A03435 | 1521 | 1.117 | 1.531 | Probable alanine aminotransferase, mitochondrial |
| A03438 | 861 | 1.094 | 1.443 | Mitochondrial import inner membrane translocase subunit TIM23 |
| A03448 | 267 | 2.689 | 5.218 | cAMP-independent regulatory protein pac2 |
| A03480 | 1488 | 1.184 | 1.696 | Arrestin (or S-antigen), N-terminal domain |
| A03502 | 1230 | 1.192 | 1.501 | Ubiquitin-like domain-containing protein |
| A03518 | 492 | 3.315 | 1.333 | 6-phosphogluconolactonase |
| A03549 | 534 | 1.291 | 1.894 | Velvet complex subunit B |
| A03554 | 888 | 1.891 | 3.392 | Poly(ADP-ribose) polymerase and DNA-Ligase Zn-finger region |
| A03557 | 2157 | 1.332 | 2.035 | Beta-glucan synthesis-associated protein KRE6 |
| A03600 | 1503 | 1.895 | 2.577 | DUF6534 domain-containing protein |
| A03608 | 717 | 4.647 | 14.400 | Extracellular membrane protein CFEM domain-containing protein |
| A03619 | 531 | 1.443 | 1.483 | P-type phospholipid transporter |
| A03644 | 1527 | 1.495 | 1.742 | Cytochrome P450 monooxygenase aclL |
| A03669 | 1176 | 2.791 | 7.594 | Probable RNA 3'-terminal phosphate cyclase-like protein |
| A03680 | 624 | 3.850 | 13.886 | Zinc knuckle |
| A03683 | 213 | 3.427 | 5.572 | Sodium channel protein |
| A03706 | 738 | 1.274 | 1.906 | Eukaryotic translation initiation factor 6 |
| A03710 | 1632 | 1.974 | 4.213 | Purine-cytosine permease fcyB |
| A03713 | 1563 | 2.268 | 5.298 | ATP-dependent rRNA helicase RRP3 |
| A03726 | 1713 | 1.492 | 2.296 | Amylosucrase |
| A03736 | 426 | 1.173 | 1.647 | Pre-mRNA-processing factor 19 |
| A03750 | 1158 | 1.804 | 3.299 | Deoxyhypusine synthase |
| A03768 | 1605 | 1.405 | 2.270 | Tubulin gamma chain |
| A03771 | 426 | 1.060 | 1.339 | Proteophosphoglycan ppg4 |
| A03785 | 387 | 1.216 | 1.786 | Ubiquitin-60S ribosomal protein L40 |
| A03789 | 3300 | 1.335 | 1.729 | Sodium/potassium-transporting ATPase subunit alpha-A |
| A03810 | 1386 | 2.104 | 4.286 | Aspartic protease |
| A03820 | 519 | 1.160 | 1.652 | 60S ribosomal protein L20-A |
| A03826 | 255 | 1.340 | 2.052 | Non-histone chromosomal protein 6 |
| A03848 | 897 | 1.336 | 1.523 | Putative hydro-lyase C5H10.01 |
| A03859 | 1026 | 1.280 | 1.804 | Cytoplasmic tRNA 2-thiolation protein 1 |
| A03867 | 318 | 2.183 | 4.200 | Mediator of RNA polymerase II transcription subunit 9 |
| A03898 | 912 | 4.899 | 20.579 | DUF937 domain-containing protein |
| A03902 | 939 | 3.329 | 5.206 | Pericentrin/AKAP-450 centrosomal targeting domain-containing protein |
| A03947 | 1941 | 1.269 | 1.674 | Iron-sulfur clusters transporter ATM1, mitochondrial |
| A03969 | 1005 | 2.006 | 4.174 | U1-type domain-containing protein |
| A03981 | 549 | 1.432 | 1.663 | Psilocybin synthase |
| A03982 | 990 | 1.688 | 3.053 | Protein bcp1 |
| A03999 | 1746 | 1.723 | 2.515 | Arabinose-proton symporter |
| A04031 | 660 | 1.352 | 2.029 | Putative tRNA (cytidine(32)/guanosine(34)-2'-O)-methyltransferase |
| A04038 | 609 | 1.676 | 2.851 | Transcriptional activator HAP5 |
| A04043 | 492 | 3.476 | 8.013 | PAN-3 domain-containing protein |
| A04076 | 1875 | 1.508 | 2.652 | Siderophore iron transporter mirB |
| A04085 | 453 | 2.192 | 4.055 | EthD domain-containing protein |
| A04090 | 1518 | 2.786 | 7.737 | Protein SOF1 |
| A04102 | 579 | 1.646 | 3.085 | 40S ribosomal protein S7 |
| A04120 | 969 | 1.053 | 1.323 | Zinc finger Ran-binding domain-containing protein 2 |
| A04124 | 1743 | 2.040 | 4.433 | Ribosome production factor 1 |
| A04140 | 1023 | 2.252 | 4.579 | L-asparaginase 2-1 |
| A04144 | 981 | 1.338 | 1.368 | REJ domain-containing protein |
| A04149 | 243 | 3.148 | 5.402 | Zinc-binding loop region of homing endonuclease domain-containing protein |
| A04154 | 1680 | 1.097 | 1.494 | General amino acid permease AGP2 |
| A04156 | 390 | 1.303 | 2.022 | 60S ribosomal protein L35-4 |
| A04173 | 1926 | 2.939 | 4.625 | NAD-dependent histone deacetylase HST3 |
| A04206 | 1230 | 1.206 | 1.730 | 50S ribosomal protein L4 |
| A04207 | 279 | 1.198 | 1.743 | 60S ribosomal protein L37a |
| A04224 | 411 | 1.685 | 3.205 | 60S ribosomal protein L27-A |
| A04234 | 327 | 1.095 | 1.486 | 40S ribosomal protein S25-A |
| A04242 | 771 | 1.457 | 2.419 | GrpE protein homolog, mitochondrial |
| A04258 | 384 | 2.031 | 4.204 | Protein of unknown function (DUF3128) |
| A04259 | 462 | 2.934 | 7.339 | DNA-directed RNA polymerase I subunit RPA12 |
| A04260 | 1602 | 1.954 | 3.908 | Exostosin family |
| A04266 | 429 | 1.305 | 2.033 | 40S ribosomal protein S16-A |
| A04267 | 849 | 2.242 | 5.031 | Nucleolar protein 16 |
| A04336 | 660 | 2.400 | 5.606 | BTB domain-containing protein |
| A04338 | 696 | 2.632 | 5.305 | BTB domain-containing protein |
| A04348 | 1230 | 1.225 | 1.696 | Peroxisomal biogenesis factor 11 |
| A04376 | 321 | 2.271 | 5.490 | 60S ribosomal protein L33-B |
| A04378 | 1050 | 1.657 | 2.465 | Fe2OG dioxygenase domain-containing protein |
| A04404 | 234 | 2.026 | 2.386 | DASH complex subunit DAD4 |
| A04422 | 1023 | 1.057 | 1.330 | Probable acyl-CoA dehydrogenase IBR3 |
| A04427 | 522 | 3.315 | 1.333 | Decarboxylase NovR |
| A04447 | 936 | 4.021 | 8.943 | SGNH/GDSL hydrolase family protein |
| A04452 | 717 | 7.536 | 3.989 | SGNH/GDSL hydrolase family protein |
| A04453 | 1575 | 1.218 | 1.641 | SGNH hydrolase-type esterase domain-containing protein |
| A04455 | 1095 | 2.427 | 4.158 | SGNH hydrolase-type esterase domain-containing protein |
| A04461 | 1116 | 1.168 | 1.588 | Exostosin-like 2 |
| A04467 | 1428 | 3.315 | 7.523 | SGNH hydrolase-type esterase domain-containing protein |
| A04479 | 1116 | 1.169 | 1.522 | HMG (high mobility group) box |
| A04482 | 570 | 2.964 | 8.181 | 60S ribosome subunit biogenesis protein nip7 |
| A04490 | 1461 | 1.163 | 1.510 | SGNH hydrolase-type esterase domain-containing protein |
| A04514 | 948 | 4.725 | 18.723 | Aryl-alcohol dehydrogenase |
| A04526 | 852 | 2.545 | 6.457 | GPN-loop GTPase 3 |
| A04542 | 1695 | 1.858 | 3.558 | Carbohydrate-binding protein |
| A04584 | 621 | 2.159 | 4.640 | Adenylate kinase isoenzyme 6 homolog |
| A04629 | 420 | 1.329 | 2.094 | 60S ribosomal protein L14 |
| A04673 | 1377 | 2.949 | 7.416 | G1/S-specific cyclin CCN1 |
| A04693 | 1578 | 1.095 | 1.477 | Alpha-aminoadipic semialdehyde dehydrogenase |
| A04696 | 825 | 9.651 | 12.162 | NADP-dependent 3-hydroxy acid dehydrogenase |
| A04736 | 648 | 1.394 | 1.840 | Serine/arginine repetitive matrix protein 2 |
| A04753 | 495 | 1.394 | 1.311 | DUF6533 domain-containing protein |
| A04815 | 345 | 1.696 | 2.915 | Histone deacetylase domain-containing protein |
| A04816 | 1599 | 1.470 | 2.504 | Purine-cytosine permease fcyB |
| A04833 | 696 | 1.159 | 1.642 | (4-O-methyl)-D-glucuronate--lignin esterase |
| A04843 | 783 | 2.276 | 4.301 | Protein O-GlcNAc transferase |
| A04850 | 1050 | 2.746 | 6.643 | Formin like 3 |
| A04861 | 654 | 1.082 | 1.375 | 54S ribosomal protein L32, mitochondrial |
| A04871 | 603 | 1.672 | 3.174 | 60S ribosomal protein L16 |
| A04896 | 513 | 1.916 | 3.225 | Cytoplasmic protein |
| A04936 | 771 | 1.205 | 1.766 | 40S ribosomal protein S1 |
| A04938 | 378 | 1.680 | 3.185 | 60S ribosomal protein L31 |
| A04950 | 381 | 1.422 | 2.331 | Neuronal calcium sensor 1 |
| A04951 | 2145 | 2.320 | 4.838 | Lipase (class 3) |
| A04953 | 1125 | 1.248 | 1.656 | D-arabinose 1-dehydrogenase |
| A04962 | 327 | 2.833 | 6.199 | Transmembrane protein |
| A04973 | 603 | 1.649 | 2.618 | Apple domain-containing protein |
| A04996 | 1656 | 3.120 | 8.280 | Probable glucan 1,3-beta-glucosidase A |
| A05001 | 423 | 1.370 | 2.220 | 60S ribosomal protein L23-A |
| A05136 | 417 | 2.027 | 3.656 | Uncharacterized protein C3B9.05 |
| A05140 | 216 | 4.536 | 10.719 | Dehydrin |
| A05141 | 360 | 4.958 | 17.869 | Dehydrin |
| A05155 | 207 | 1.211 | 1.583 | HMG box domain-containing protein |
| A05163 | 600 | 4.042 | 5.305 | MBD domain-containing protein |
| A05171 | 1185 | 1.341 | 2.123 | Obg-like ATPase 1 |
| A05180 | 456 | 1.083 | 1.346 | Negative cofactor 2 complex subunit beta |
| A05181 | 381 | 1.383 | 2.255 | 60S ribosomal protein L32 |
| A05194 | 393 | 1.491 | 2.584 | 40S ribosomal protein S22-A |
| A05222 | 267 | 6.411 | 10.632 | FH2 domain-containing protein |
| A05231 | 966 | 3.753 | 9.013 | SRCR domain-containing protein |
| A05232 | 420 | 3.186 | 7.779 | Small ribosomal subunit protein bS18m |
| A05280 | 222 | 2.240 | 2.229 | Glutamine--tRNA ligase |
| A05284 | 1251 | 1.561 | 2.664 | rRNA biogenesis protein rrp36 |
| A05290 | 2004 | 1.551 | 2.428 | Dihydroxyacetone synthase |
| A05298 | 642 | 1.135 | 1.374 | F-box domain-containing protein |
| A05352 | 1032 | 5.359 | 23.609 | DUF937 domain-containing protein |
| A05406 | 525 | 7.016 | 2.834 | Extracellular membrane protein CFEM domain-containing protein |
| A05409 | 975 | 1.828 | 3.351 | Cytoplasmic tRNA 2-thiolation protein 1 |
| A05424 | 966 | 3.559 | 11.145 | Putative hydro-lyase C5H10.01 |
| A05425 | 1128 | 3.482 | 6.149 | MIA SH3 domain ER export factor 3 |
| A05439 | 1305 | 1.307 | 2.037 | Solute carrier RCH1 |
| A05443 | 1677 | 1.418 | 2.272 | L-gulonolactone oxidase |
| A05444 | 306 | 1.111 | 1.312 | 37S ribosomal protein S19, mitochondrial |
| A05456 | 519 | 1.698 | 3.257 | 60S ribosomal protein L20-A |
| A05459 | 1065 | 1.592 | 2.806 | Multiple RNA-binding domain-containing protein 1 |
| A05465 | 1665 | 1.173 | 1.689 | Major facilitator-type transporter ecdD |
| A05496 | 240 | 2.667 | 5.901 | SMP domain-containing protein |
| A05529 | 987 | 1.143 | 1.530 | Protein bcp1 |
| A05546 | 996 | 2.158 | 4.770 | U1-type domain-containing protein |
| A05630 | 486 | 1.243 | 1.513 | Prefoldin subunit |
| A05634 | 243 | 3.041 | 8.598 | Cytochrome |
| A05636 | 339 | 3.593 | 11.962 | GTPase activating protein |
| A05656 | 585 | 1.125 | 1.506 | rRNA-processing protein FCF1 homolog |
| A05675 | 1473 | 1.544 | 2.287 | box-containing protein C19G7.04 |
| A05689 | 1563 | 1.373 | 2.148 | L-serine dehydratase |
| A05707 | 531 | 1.380 | 2.255 | 60S ribosomal protein L11 |
| A05747 | 291 | 5.408 | 22.023 | Stress-induced protein |
| A05748 | 597 | 4.536 | 10.719 | Barwin-like endoglucanase |
| A05807 | 426 | 1.295 | 1.851 | Catalase |
| A05817 | 1308 | 1.797 | 3.406 | Mitochondrial carrier protein RIM2 |
| A05860 | 498 | 1.362 | 1.997 | NADH-quinone oxidoreductase |
| A05868 | 408 | 1.344 | 2.134 | SnoaL-like domain-containing protein |
| A05878 | 369 | 1.247 | 1.628 | Transmembrane protein |
| A05904 | 234 | 1.867 | 3.031 | Methyl-accepting transducer domain-containing protein |
| A05917 | 1839 | 1.900 | 3.791 | DUF4048 domain-containing protein |
| A05977 | 717 | 1.809 | 2.748 | N-acetyltransferase domain-containing protein |
| A05982 | 3840 | 1.380 | 2.235 | ABC multidrug transporter MDR1 |
| A06008 | 1068 | 1.615 | 2.812 | Hydroxyquinol 1,2-dioxygenase |
| A06040 | 1224 | 1.623 | 2.677 | G-protein coupled receptors family 1 profile domain-containing protein |
| A06051 | 1758 | 1.193 | 1.706 | High-affinity methionine permease |
| A06056 | 1176 | 3.555 | 11.848 | Lovastatin esterase |
| A06061 | 891 | 2.689 | 7.419 | alpha/beta hydrolase fold |
| A06065 | 648 | 1.215 | 1.643 | Ribosome assembly protein 3 |
| A06090 | 918 | 1.238 | 1.711 | Serine/threonine-protein phosphatase 4 catalytic subunit |
| A06097 | 609 | 1.176 | 1.468 | Exosome complex component csl4 |
| A06104 | 243 | 2.281 | 4.412 | Swi5-domain-containing protein |
| A06107 | 828 | 1.371 | 2.096 | KOW motif |
| A06110 | 1323 | 1.401 | 1.594 | Mitochondrial distribution and morphology protein 12 |
| A06121 | 744 | 2.013 | 3.850 | Ribosome biogenesis protein SLX9 |
| A06126 | 489 | 1.569 | 2.559 | MYND-type domain-containing protein |
| A06134 | 495 | 1.316 | 1.884 | 54S ribosomal protein L23, mitochondrial |
| A06137 | 2727 | 1.615 | 2.975 | Fungal specific transcription factor domain |
| A06141 | 615 | 1.519 | 2.680 | 60S ribosomal protein L1-B |
| A06158 | 780 | 1.444 | 2.093 | Mannan endo-1,4-beta-mannosidase 1 |
| A06160 | 1308 | 2.390 | 6.003 | FAD-dependent urate hydroxylase |
| A06165 | 351 | 1.431 | 2.099 | Pre-rRNA-processing protein |
| A06178 | 1500 | 3.626 | 8.384 | SGNH hydrolase-type esterase domain-containing protein |
| A06184 | 1350 | 1.118 | 1.532 | Glutaryl-CoA dehydrogenase, mitochondrial |
| A06195 | 375 | 2.025 | 3.396 | DUF4050 domain-containing protein |
| A06197 | 1104 | 1.900 | 3.738 | Pre-rRNA-processing protein ESF2 |
| A06199 | 1773 | 1.137 | 1.399 | Cell cycle serine/threonine-protein kinase hsk1 |
| A06203 | 1086 | 1.719 | 2.641 | Histone chaperone domain-containing protein |
| A06216 | 1557 | 2.708 | 4.987 | Glycosyltransferase family 43 |
| A06225 | 822 | 2.497 | 6.425 | Zinc knuckle |
| A06227 | 483 | 1.257 | 1.897 | 40S ribosomal protein S19-B |
| A06266 | 243 | 2.391 | 2.394 | Cyclin-dependent kinases regulatory subunit |
| A06277 | 2820 | 2.157 | 4.855 | Proteophosphoglycan ppg4 |
| A06290 | 1581 | 1.988 | 4.106 | Mini-chromosome maintenance complex-binding protein |
| A06296 | 321 | 1.543 | 2.360 | Pet100 |
| A06312 | 1245 | 1.396 | 2.223 | Peroxisomal biogenesis factor 11 |
| A06329 | 1008 | 4.837 | 19.821 | Aryl-alcohol dehydrogenase |
| A06330 | 852 | 1.146 | 1.572 | GPN-loop GTPase 3 |
| A06351 | 648 | 1.540 | 2.186 | Serine/arginine repetitive matrix protein 2 |
| A06382 | 891 | 2.053 | 3.647 | Uncharacterized transporter C22E12.01 |
| A06388 | 1443 | 1.977 | 3.999 | SGNH/GDSL hydrolase family protein |
| A06389 | 936 | 1.807 | 2.589 | SGNH hydrolase-type esterase domain-containing protein |
| A06418 | 1716 | 1.268 | 1.896 | RNase III domain-containing protein |
| A06436 | 1761 | 2.438 | 5.090 | Beta-1,3-galactosyl-O-glycosyl-glycoprotein beta-1,6-N-acetylglucosaminyltransferase 4 |
| A06440 | 1134 | 2.253 | 4.596 | SGNH hydrolase-type esterase domain-containing protein |
| A06444 | 1287 | 1.312 | 1.753 | Uncharacterized protein MJ1055 |
| A06445 | 957 | 2.523 | 4.742 | Uncharacterized transporter C22E12.01 |
| A06450 | 864 | 3.507 | 10.061 | SGNH/GDSL hydrolase family protein |
| A06451 | 801 | 2.030 | 3.791 | SGNH hydrolase-type esterase domain-containing protein |
| A06459 | 423 | 2.795 | 7.696 | VWFA domain-containing protein |
| A06462 | 1623 | 2.348 | 4.708 | Chitin synthase export chaperone |
| A06463 | 321 | 1.515 | 1.521 | 3-ketoacyl-CoA thiolase, peroxisomal |
| A06468 | 1860 | 1.223 | 1.799 | FAD-binding monooxygenase moxY |
| A06476 | 606 | 1.081 | 1.399 | DRBM domain-containing protein |
| A06480 | 528 | 1.663 | 2.799 | RNA polymerase II subunit A C-terminal domain phosphatase SSU72 |
| A06493 | 411 | 2.268 | 3.970 | CID domain-containing protein |
| A06499 | 810 | 1.603 | 2.257 | Ribonuclease P 40kDa (Rpp40) subunit |
| A06503 | 729 | 1.583 | 2.713 | RING-type domain-containing protein |
| A06514 | 414 | 2.606 | 3.146 | PHD-type domain-containing protein |
| A06523 | 1248 | 1.936 | 3.432 | REJ domain-containing protein |
| A06542 | 2637 | 1.074 | 1.428 | Something about silencing protein 10 |
| A06552 | 852 | 1.307 | 2.043 | 40S ribosomal protein S4-C |
| A06553 | 729 | 3.051 | 6.709 | Uncharacterized protein C6G9.01c |
| A06564 | 288 | 1.180 | 1.543 | Dynein light chain 1, cytoplasmic |
| A06573 | 3762 | 1.041 | 1.348 | Uncharacterized ABC transporter ATP-binding protein/permease YOL075C |
| A06587 | 630 | 1.311 | 1.999 | Serine/arginine-rich splicing factor 4 |
| A06616 | 270 | 3.637 | 7.464 | Protein adenylyltransferase SelO |
| A06638 | 444 | 1.053 | 1.383 | 40S ribosomal protein S15 |
| A06640 | 768 | 1.830 | 3.739 | 60S ribosomal protein L8 |
| A06680 | 279 | 1.224 | 1.800 | 60S ribosomal protein L38-1 |
| A06700 | 3276 | 2.397 | 6.123 | Probable importin subunit beta-4 |
| A06722 | 1455 | 2.730 | 5.600 | Srp40 C-terminal domain-containing protein |
| A06761 | 309 | 1.399 | 1.959 | Mitochondrial export protein Som1 |
| A06765 | 348 | 1.784 | 3.018 | Replication factor A protein 3 |
| A06779 | 450 | 1.117 | 1.537 | 60S ribosomal protein L24-A |
| A06806 | 288 | 1.945 | 4.163 | Probable 60S ribosomal protein L37-A |
| A06814 | 4110 | 3.730 | 13.413 | Calcium-transporting ATPase 2 |
| A06816 | 684 | 1.264 | 1.810 | Ribosomal prokaryotic L21 protein |
| A06817 | 801 | 1.881 | 3.015 | Nnf1-domain-containing protein |
| A06827 | 231 | 1.954 | 4.150 | Histone chaperone domain-containing protein |
| A06842 | 780 | 2.623 | 1.687 | DUF6534 domain-containing protein |
| A06846 | 1770 | 3.639 | 12.769 | Catalase |
| A06847 | 516 | 4.397 | 9.947 | SH3 domain-containing protein |
| A06879 | 948 | 1.089 | 1.444 | Holocytochrome-c synthase |
| A06886 | 1758 | 2.353 | 3.771 | Putative serine esterase MT1883 |
| A06900 | 408 | 4.415 | 15.621 | Histone H3.2 |
| A06929 | 258 | 1.671 | 1.961 | Putative ABC transport system ATP-binding protein |
| A06962 | 555 | 2.193 | 2.821 | C6 transcription factor |
| A06965 | 990 | 1.737 | 3.157 | -ABC transmembrane type-1 domain-containing protein |
| A07025 | 1668 | 1.432 | 2.416 | Probable metabolite transporter C2H8.02 |
| A07041 | 951 | 1.964 | 3.108 | ATPase domain-containing protein |
| A07087 | 1359 | 1.476 | 2.227 | Probable kinetochore protein NUF2 |
| A07110 | 1356 | 1.057 | 1.328 | NADH-dependent flavin oxidoreductase iliE |
| A07136 | 1395 | 1.687 | 2.283 | Glycosyl Hydrolase Family 88 |
| A07137 | 1440 | 1.233 | 1.633 | Glycosyl Hydrolase Family 88 |
| A07168 | 1086 | 1.194 | 1.652 | Integral membrane protein DUF92 |
| A07170 | 456 | 1.099 | 1.498 | 40S ribosomal protein S13-1 |
| A07202 | 1164 | 2.075 | 3.620 | Kinetochore protein Sos7 coiled-coil domain-containing protein |
| A07210 | 729 | 3.692 | 4.165 | CLASP N terminal |
| A07221 | 747 | 1.175 | 1.491 | Histone acetyltransferase subunit NuA4 |
| A07240 | 294 | 4.202 | 5.881 | Heat shock-like 85 kDa protein |
| A07244 | 672 | 5.305 | 16.125 | non-specific serine/threonine protein kinase |
| A07249 | 888 | 3.779 | 7.006 | Exonuclease 1 |
| A07264 | 492 | 1.137 | 1.545 | DNA-directed RNA polymerase II subunit RPB11 |
| A07279 | 474 | 1.210 | 1.621 | 50S ribosomal protein L28 |
| A07287 | 408 | 4.109 | 12.937 | Histone H3.2 |
| A07292 | 585 | 1.885 | 2.338 | Ribosome assembly protein 3 |
| A07295 | 1935 | 1.856 | 3.721 | Uncharacterized protein C1604.06c |
| A07306 | 660 | 5.002 | 18.493 | Glutathione-dependent formaldehyde-activating enzyme |
| A07326 | 777 | 1.646 | 2.702 | PPE family domain-containing protein |
| A07329 | 1767 | 1.300 | 1.970 | FAD-binding monooxygenase moxY |
| A07355 | 1326 | 1.161 | 1.395 | Aspyridones efflux protein apdF |
| A07358 | 669 | 1.315 | 1.711 | Auxin efflux carrier component |
| A07367 | 555 | 1.444 | 2.122 | RNA polymerase II-binding domain. |
| A07371 | 1632 | 2.749 | 6.227 | UDP-galactose translocator |
| A07374 | 681 | 1.334 | 1.787 | Ribonuclease P protein subunit p40 |
| A07380 | 1023 | 1.213 | 1.717 | Nucleoporin Nup159/Nup146 N-terminal domain-containing protein |
| A07395 | 297 | 1.696 | 2.244 | SMP domain-containing protein |
| A07404 | 1062 | 2.582 | 5.200 | Chitinase |
| A07470 | 558 | 1.680 | 2.076 | Antifreeze glycopeptide AFGP poly protein |
| A07475 | 1521 | 1.146 | 1.590 | Mitochondrial import inner membrane translocase subunit TIM44 |
| A07509 | 1041 | 1.286 | 1.937 | Short-chain dehydrogenase/reductase VdtF |
| A07528 | 1755 | 2.510 | 6.643 | Amino-acid permease inda1 |
| A07530 | 1725 | 2.871 | 8.120 | Amino-acid permease inda1 |
| A07540 | 1086 | 1.138 | 1.569 | Proliferating cell nuclear antigen |
| A07572 | 1155 | 1.637 | 2.894 | SH3 domain-containing protein |
| A07575 | 1116 | 2.714 | 7.315 | DUF6533 domain-containing protein |
| A07577 | 1020 | 5.381 | 19.374 | FlgD/Vpr Ig-like domain-containing protein |
| A07584 | 1572 | 1.245 | 1.875 | NADP-dependent malic enzyme |
| A07591 | 1203 | 1.233 | 1.715 | Adenylyltransferase and sulfurtransferase UBA4 |
| A07592 | 282 | 2.050 | 4.616 | Conidiation-specific protein 10 |
| A07593 | 1422 | 1.811 | 3.540 | C2H2-type domain-containing protein |
| A07595 | 3180 | 2.078 | 4.591 | Checkpoint serine/threonine-protein kinase BUB1 |
| A07596 | 564 | 2.954 | 7.274 | Spindle assembly checkpoint component MAD3 |
| A07602 | 966 | 8.098 | 5.599 | Proteophosphoglycan ppg4 |
| A07614 | 495 | 1.076 | 1.415 | Lactoylglutathione lyase |
| A07623 | 1245 | 1.888 | 3.704 | Pre-rRNA-processing protein las1 |
| A07626 | 405 | 2.198 | 3.211 | ATP-binding cassette domain-containing protein |
| A07643 | 666 | 1.160 | 1.413 | DNA replication complex GINS protein PSF1 |
| A07644 | 729 | 1.529 | 2.360 | Dolichol-phosphate mannosyltransferase |
| A07645 | 1668 | 2.649 | 6.892 | ATP-dependent RNA helicase ROK1 |
| A07650 | 561 | 1.398 | 1.826 | Guanine nucleotide exchange factor MSS4 |
| A07704 | 408 | 3.931 | 12.810 | Histone H3.2 |
| A07764 | 612 | 1.327 | 1.916 | SH3b domain-containing protein |
| A07768 | 291 | 1.165 | 1.539 | Leydig cell tumor 10 kDa protein homolog |
| A07769 | 513 | 3.955 | 8.242 | Tyrosine-protein kinase ephrin type A/B receptor-like domain-containing protein |
| A07776 | 666 | 2.196 | 4.459 | Serine/threonine-protein phosphatase |
| A07783 | 513 | 1.576 | 2.849 | 40S ribosomal protein S14 |
| A07792 | 1413 | 2.386 | 3.278 | Borealin N-terminal domain-containing protein |
| A07836 | 2223 | 3.644 | 7.774 | Inhibitor of Apoptosis domain |
| A07854 | 438 | 2.641 | 3.015 | Extracellular membrane protein CFEM domain-containing protein |
| A07880 | 720 | 1.229 | 1.703 | Nuclear transcription factor Y subunit B-10 |
| A07891 | 765 | 1.079 | 1.368 | Dynamin-binding protein |
| A07902 | 1713 | 1.113 | 1.351 | UDP-glycosyltransferase 84B1 |
| A07997 | 420 | 1.591 | 2.901 | 60S ribosomal protein L14 |
| A08024 | 450 | 1.366 | 1.850 | SEA domain-containing protein |
| A08032 | 2160 | 1.127 | 1.562 | REJ domain-containing protein |
| A08066 | 867 | 4.297 | 9.428 | NADP-dependent 3-hydroxy acid dehydrogenase |
| A08076 | 3120 | 1.282 | 1.951 | Importin-11 |
| A08109 | 1002 | 2.044 | 2.644 | Glycosyl transferase CAP10 domain-containing protein |
| A08160 | 1962 | 2.673 | 4.565 | Kinesin-like protein 6 |
| A08176 | 1011 | 2.590 | 5.230 | Disease resistance N-terminal domain-containing protein |
| A08179 | 1599 | 1.981 | 4.284 | Purine-cytosine permease fcyB |
| A08181 | 1515 | 1.148 | 1.402 | Uncharacterized transporter C757.13 |
| A08192 | 621 | 1.428 | 2.000 | Efflux RND transporter periplasmic adaptor subunit |
| A08195 | 258 | 4.499 | 9.092 | Peroxisomal membrane protein PEX13 |
| A08196 | 501 | 2.650 | 6.940 | (4-O-methyl)-D-glucuronate--lignin esterase |
| A08209 | 954 | 6.349 | 10.324 | EH domain-containing protein |
| A08226 | 750 | 1.756 | 3.463 | 60S ribosomal protein L16 |
| A08227 | 381 | 2.398 | 3.855 | LIM domain |
| A08231 | 1692 | 1.676 | 2.235 | MFS-type transporter ptmT |
| A08240 | 1455 | 1.526 | 2.473 | Ammonium transporter 1 |
| A08252 | 852 | 2.717 | 5.699 | Ammonium transporter MEP1 |
| A08282 | 1167 | 1.362 | 2.157 | Fe-regulated protein 8 |
| A08283 | 1650 | 1.067 | 1.364 | Efflux pump atB |
| A08289 | 378 | 1.365 | 2.203 | 60S ribosomal protein L31 |
| A08310 | 327 | 1.708 | 2.658 | Transmembrane protein |
| A08321 | 309 | 4.974 | 4.813 | Mid2 domain-containing protein |
| A08337 | 1353 | 2.692 | 6.699 | Probable glucan 1,3-beta-glucosidase A |
| A08342 | 423 | 1.389 | 2.278 | 60S ribosomal protein L23-A |
| A08353 | 582 | 1.750 | 3.196 | DNA-directed RNA polymerase III subunit Rpc31 |
| A08364 | 2379 | 1.066 | 1.399 | Homocitrate dehydratase, mitochondrial |
| A08376 | 753 | 1.326 | 1.927 | Cation transport protein |
| A08377 | 312 | 1.851 | 3.043 | Potassium transport protein 1 |
| A08434 | 1968 | 1.062 | 1.335 | [histone H3]-lysine(27) N-trimethyltransferase |
| A08459 | 216 | 1.517 | 2.581 | Dehydrin |
| A08460 | 360 | 4.270 | 15.920 | Dehydrin |
| A08467 | 3051 | 1.248 | 1.850 | Sensor histidine kinase AruS |
| A08472 | 447 | 1.764 | 3.229 | Mitochondrial import inner membrane translocase subunit tim16 |
| A08483 | 1209 | 1.555 | 2.757 | Obg-like ATPase 1 |
| A08493 | 381 | 1.726 | 3.356 | 60S ribosomal protein L32 |
| A08506 | 393 | 1.460 | 2.483 | 40S ribosomal protein S22-A |
| A08538 | 1029 | 1.514 | 2.480 | Granulins domain-containing protein |
| A08554 | 783 | 1.723 | 2.787 | Probable kinetochore protein SPC25 |
| A08581 | 2310 | 2.813 | 7.203 | Cell wall protein |
| A08584 | 1089 | 1.348 | 2.000 | Chitobiosyldiphosphodolichol beta-mannosyltransferase |
| A08590 | 639 | 1.126 | 1.469 | Mitochondrial inner membrane protease subunit 2 |
| A08598 | 1509 | 1.282 | 1.883 | Fungal specific transcription factor domain |
| A08614 | 1878 | 2.774 | 7.986 | Siderophore iron transporter mirB |
| A08620 | 447 | 5.154 | 8.158 | MYND-type domain-containing protein |
| A08624 | 2022 | 1.452 | 2.371 | Beta-glucan synthesis-associated protein KRE6 |
| A08634 | 579 | 1.893 | 3.976 | 40S ribosomal protein S7 |
| A08654 | 1716 | 1.431 | 2.341 | Ribosome production factor 1 |
| A08666 | 1548 | 2.761 | 6.783 | 5'-nucleotidase |
| A08672 | 963 | 2.703 | 5.051 | L-asparaginase 2-1 |
| A08673 | 963 | 2.972 | 4.522 | L-asparaginase 2-1 |
| A08678 | 828 | 3.030 | 4.957 | BTB domain-containing protein |
| A08683 | 432 | 7.406 | 3.667 | Zinc-ribbon 15 domain-containing protein |
| A08697 | 612 | 2.323 | 3.378 | Expansin-like EG45 domain-containing protein |
| A08703 | 1833 | 7.185 | 3.177 | NAD-dependent histone deacetylase HST3 |
| A08716 | 402 | 1.870 | 1.585 | DNA mismatch repair protein MutL |
| A08733 | 882 | 1.518 | 2.533 | Sin-like protein conserved region |
| A08734 | 462 | 1.756 | 3.304 | C2H2-type domain-containing protein |
| A08736 | 279 | 1.421 | 2.372 | 60S ribosomal protein L37a |
| A08763 | 315 | 1.066 | 1.333 | Lysosomal amino acid transporter 1 homolog |
| A08773 | 741 | 1.433 | 2.354 | Orotate phosphoribosyltransferase |
| A08791 | 1281 | 1.303 | 1.985 | Ubiquitin-like modifier-activating enzyme ATG7 |
| A08814 | 2703 | 1.395 | 2.223 | Glutathione S-transferase, N-terminal domain |
| A08817 | 1386 | 1.116 | 1.523 | Ribosome-associated complex head domain |
| A08827 | 7083 | 1.154 | 1.633 | small nucleolar RNA-associated protein 10 |
| A08846 | 633 | 1.801 | 1.356 | AP2/ERF domain-containing protein |
| A08853 | 312 | 1.822 | 3.444 | DUF202 domain-containing protein |
| A08861 | 3900 | 1.338 | 2.141 | Fatty acid synthase subunit beta |
| A08877 | 1026 | 2.180 | 4.468 | 25S rRNA (uridine(2843)-N(3))-methyltransferase |
| A08899 | 1512 | 2.700 | 7.226 | DNA-directed RNA polymerase I subunit rpa49 |
| A08906 | 387 | 1.669 | 3.155 | 60S ribosomal protein L22 |
| A08913 | 444 | 7.262 | 3.342 | Barwin domain-containing protein |
| A08916 | 360 | 1.470 | 1.913 | DASH complex subunit Dad2 |
| A08917 | 399 | 1.157 | 1.345 | Protein kinase C terminal domain |
| A08919 | 813 | 2.437 | 4.368 | Serine/threonine-protein kinase cbk1 |
| A08974 | 1539 | 1.054 | 1.306 | ATP-grasp domain-containing protein |
| A08995 | 720 | 1.303 | 1.634 | Mitotic spindle checkpoint component mad2 |
| A09030 | 546 | 1.757 | 1.869 | IQ domain-containing protein IQM6 |
| A09050 | 675 | 2.036 | 4.266 | Ribonuclease E |
| A09078 | 657 | 1.895 | 3.279 | F-box domain-containing protein |
| A09083 | 387 | 1.241 | 1.559 | Glutathione-dependent formaldehyde-activating enzyme |
| A09109 | 609 | 2.607 | 6.478 | Translation machinery-associated protein 16 |
| A09128 | 264 | 1.223 | 1.802 | 40S ribosomal protein S21 |
| A09156 | 372 | 1.877 | 2.835 | Uncharacterized conserved protein (DUF2340) |
| A09157 | 2112 | 1.090 | 1.313 | Sfi1 spindle body protein |
| A09168 | 1854 | 1.159 | 1.643 | Manganese transporter pdt1 |
| A09180 | 2862 | 1.050 | 1.367 | Copper-transporting ATPase HMA5 |
| A09197 | 426 | 1.188 | 1.617 | Variant SH3 domain |
| A09244 | 654 | 3.134 | 4.516 | Uncharacterized protein YpgQ |
| A09247 | 486 | 1.248 | 1.639 | Prefoldin subunit |
| A09250 | 906 | 1.082 | 1.461 | 60S ribosomal protein L5-B |
| A09252 | 252 | 3.029 | 8.597 | Cytochrome |
| A09275 | 585 | 2.207 | 5.063 | rRNA-processing protein FCF1 homolog |
| A09296 | 1614 | 1.567 | 2.436 | Eukaryotic aspartyl protease |
| A09297 | 1662 | 2.383 | 4.624 | High mobility group box-containing protein C19G7.04 |
| A09313 | 1350 | 2.304 | 5.347 | L-serine dehydratase |
| A09315 | 1833 | 1.764 | 3.483 | Amidophosphoribosyltransferase |
| A09321 | 510 | 1.637 | 2.434 | DASH complex subunit Duo1 |
| A09333 | 531 | 1.429 | 2.398 | 60S ribosomal protein L11 |
| A09347 | 417 | 1.205 | 1.523 | Transport protein particle subunit trs23 |
| A09351 | 585 | 1.257 | 1.756 | Cytosolic iron-sulfur assembly component 2B |
| A09377 | 291 | 1.774 | 3.527 | Stress-induced protein |
| A09387 | 972 | 1.362 | 2.103 | Integrin beta-1-binding protein 2 |
| A09410 | 915 | 1.251 | 1.313 | Probable hydrolase nit2 |
| A09412 | 471 | 1.300 | 1.572 | Nuclear segregation protein |
| A09448 | 1062 | 1.294 | 1.905 | Uncharacterized mitochondrial carrier C688.09 |
| A09449 | 819 | 3.256 | 3.587 | GTP-binding protein rho4 |
| A09460 | 411 | 1.564 | 2.798 | 60S ribosomal protein L27-A |
| A09493 | 369 | 2.880 | 7.845 | Protein of unknown function (DUF3128) |
| A09494 | 387 | 2.633 | 6.134 | DNA-directed RNA polymerase I subunit RPA12 |
| A09497 | 2037 | 3.784 | 13.583 | Phenol hydroxylase |
| A09502 | 429 | 1.211 | 1.779 | 40S ribosomal protein S16-A |
| A09503 | 1467 | 1.158 | 1.631 | Alpha-glucosidase |
| A09510 | 855 | 1.288 | 1.959 | Srp40 C-terminal domain-containing protein |
| A09573 | 1467 | 1.576 | 2.748 | Pre-rRNA-processing protein IPI3 |
| A09578 | 1392 | 2.764 | 7.449 | Ribosome biogenesis protein YTM1 |
| A09590 | 714 | 1.706 | 3.297 | 60S ribosomal protein L2 |
| A09600 | 363 | 1.231 | 1.465 | PH domain-containing protein |
| A09610 | 1446 | 1.463 | 2.473 | N amino acid transport system protein |
| A09614 | 396 | 1.257 | 1.811 | Mitochondrial import inner membrane translocase subunit TIM14 |
| A09617 | 582 | 3.361 | 8.342 | Major facilitator superfamily (MFS) profile domain-containing protein |
| A09621 | 501 | 1.651 | 1.914 | alpha/beta hydrolase fold |
| A09655 | 1656 | 2.602 | 6.906 | Ribosome assembly protein rrb1 |
| A09656 | 693 | 1.748 | 2.403 | Trehalose utilisation |
| A09661 | 1176 | 3.053 | 9.325 | Probable diacetyl reductase [(R)-acetoin forming] 2 |
| A09667 | 969 | 1.437 | 2.360 | Aquaglycerol porin AQY3 |
| A09672 | 2388 | 1.458 | 2.473 | Bifunctional purine biosynthetic protein ADE1 |
| A09678 | 972 | 2.521 | 3.561 | EGF-like domain-containing protein |
| A09687 | 1866 | 1.564 | 2.810 | Right handed beta helix region |
| A09688 | 2415 | 1.381 | 2.271 | Siderophore iron transporter 1 |
| A09690 | 249 | 3.180 | 4.992 | Histidine kinase |
| A09717 | 1221 | 1.903 | 3.633 | Efflux pump azaK |
| A09722 | 1143 | 1.517 | 2.632 | S-(hydroxymethyl)glutathione dehydrogenase |
| A09730 | 435 | 4.005 | 10.916 | SMP domain-containing protein |
| A09740 | 744 | 1.726 | 2.387 | TBPIP/Hop2 winged helix domain |
| A09823 | 816 | 1.492 | 1.715 | Oxysterol-binding protein |
| A09842 | 600 | 2.209 | 1.801 | Esterase |
| A09847 | 285 | 1.467 | 2.511 | 40S ribosomal protein S27 |
| A09852 | 582 | 2.072 | 4.443 | tRNA N6-adenosine threonylcarbamoyltransferase |
| A09857 | 612 | 1.754 | 2.987 | Uncharacterized protein C553.12c |
| A09861 | 1368 | 1.451 | 1.998 | Transmembrane protein 184 homolog |
| A09872 | 2727 | 1.696 | 3.196 | TPR repeat-containing protein C19B12.01 |
| A09875 | 3642 | 1.466 | 2.495 | Phosphate-repressible phosphate permease pho-4 |
| A09890 | 1998 | 1.241 | 1.716 | Alpha-actinin-like protein 1 |
| A09900 | 600 | 4.179 | 10.177 | Microtubule-associated protein Jupiter |
| A09908 | 921 | 1.578 | 2.480 | Homing endonuclease LAGLIDADG domain-containing protein |
| A09913 | 1629 | 1.561 | 1.544 | DEK-C domain-containing protein |
| A09924 | 2118 | 1.874 | 3.858 | ATP-dependent RNA helicase HAS1 |
| A09925 | 1446 | 3.494 | 6.527 | Uncharacterized transporter C1683.12 |
| A09931 | 921 | 3.271 | 8.255 | Phospholipase/Carboxylesterase |
| A09945 | 465 | 1.479 | 2.531 | 60S ribosomal protein L26-2 |
| A09979 | 588 | 2.708 | 4.578 | Ricin B lectin domain-containing protein |
| A10014 | 822 | 2.466 | 2.082 | Beta-lactamase |
| A10027 | 399 | 1.149 | 1.525 | RNase III domain-containing protein |
| A10057 | 954 | 2.926 | 2.741 | THO complex subunit 2 |
| A10085 | 1665 | 2.198 | 3.648 | AAA family ATPase |
| A10093 | 573 | 1.856 | 2.600 | Probable serine/threonine-protein kinase |
| A10105 | 852 | 1.202 | 1.651 | Uncharacterized vacuolar membrane protein YML018C |
| A10117 | 1053 | 1.238 | 1.781 | Guanine nucleotide-binding protein alpha-1 subunit |
| A10131 | 216 | 1.522 | 1.344 | ATP-binding protein Uup |
| A10143 | 444 | 1.484 | 2.549 | 40S ribosomal protein S15 |
| A10158 | 579 | 1.115 | 1.455 | Ribosomal prokaryotic L21 protein |
| A10192 | 915 | 1.707 | 2.694 | F-box domain-containing protein |
| A10193 | 843 | 1.854 | 3.250 | Receptor protein kinase |
| A10210 | 444 | 3.656 | 4.055 | NACHT domain-containing protein |
| A10248 | 792 | 1.640 | 2.962 | Transcription factor Sp9 isoform X2 |
| A10320 | 1230 | 1.659 | 2.521 | L domain-like protein |
| A10326 | 318 | 1.739 | 2.953 | Pet100 |
| A10344 | 1497 | 3.067 | 7.215 | Serine/threonine-protein kinase ark1 |
| A10366 | 537 | 2.026 | 2.386 | Coiled-coil domain-containing protein SCD2 |
| A10388 | 333 | 1.247 | 1.869 | 60S ribosomal protein L36-A |
| A10395 | 333 | 2.700 | 5.423 | Small, acid-soluble spore protein I |
| A10402 | 300 | 1.859 | 3.348 | Mitochondrial import inner membrane translocase subunit tim10 |
| A10405 | 1416 | 3.374 | 9.444 | Uncharacterized transporter C460.05 |
| A10429 | 1359 | 1.785 | 3.585 | Fatty acid desaturase |
| A10431 | 1848 | 1.557 | 2.689 | Repeat-containing protein ARB_01230 |
| A10435 | 870 | 2.605 | 3.340 | Exodeoxyribonuclease 7 large subunit |
| A10450 | 528 | 1.534 | 2.711 | 60S ribosomal protein L17 |
| A10459 | 324 | 1.268 | 1.394 | PPP4R2-domain-containing protein |
| A10481 | 369 | 1.092 | 1.479 | 40S ribosomal protein S20 |
| A10484 | 750 | 1.532 | 2.360 | MYND finger |
| A10493 | 459 | 1.987 | 3.519 | Cell division control protein 31 |
| A10507 | 1620 | 1.948 | 4.196 | Thiamine transporter thi9 |
| A10529 | 711 | 3.758 | 1.975 | Sulfotransferase domain |
| A10536 | 1329 | 2.033 | 4.333 | GDP-mannose 4,6 dehydratase |
| A10537 | 294 | 1.700 | 1.514 | DRBM domain-containing protein |
| A10569 | 351 | 1.470 | 2.508 | 60S ribosomal protein L34-A |
| A10584 | 1263 | 1.440 | 2.379 | C2H2 finger domain transcription factor dvrA |
| A10618 | 645 | 3.075 | 8.824 | Ribosome biogenesis regulatory protein homolog |
| A10639 | 510 | 1.898 | 3.801 | Nuclear transport factor 2 (NTF2) domain |
| A10647 | 798 | 1.470 | 2.160 | Coiled-coil domain-containing protein 25 |
| A10648 | 498 | 1.540 | 2.737 | 60S ribosomal protein L12-A |
| A10657 | 1524 | 1.463 | 2.390 | MFS siderochrome iron transporter 1 |
| A10672 | 969 | 1.411 | 2.342 | rRNA 2'-O-methyltransferase fibrillarin |
| A10699 | 1728 | 2.041 | 3.912 | Para-nitrobenzyl esterase |
| A10794 | 600 | 1.632 | 3.038 | 60S ribosomal protein L6-2 |
| A10798 | 435 | 2.530 | 2.489 | ATP-dependent DNA helicase MER3 |
| A10800 | 1407 | 1.989 | 2.842 | SET domain |
| A10807 | 1488 | 2.324 | 3.606 | Cytosol non-specific dipeptidase |
| A10827 | 621 | 2.792 | 3.146 | DUF6533 domain-containing protein |
| A10828 | 840 | 3.570 | 8.455 | Acyltransferase |
| A10834 | 660 | 2.433 | 3.627 | Flavoprotein domain-containing protein |
| A10871 | 1728 | 2.037 | 4.468 | U3 small nucleolar RNA-associated protein 15 |
| A10875 | 831 | 2.176 | 4.798 | U3 small nucleolar ribonucleoprotein protein IMP4 |
| A10906 | 636 | 1.538 | 2.349 | Proteophosphoglycan ppg4 |
| A10934 | 576 | 1.070 | 1.359 | Protein ras-2 |
| A10935 | 372 | 2.085 | 4.577 | Proteophosphoglycan ppg4 |
| A10936 | 804 | 2.317 | 4.661 | Hemerythrin HHE cation binding domain |
| A10946 | 2217 | 1.054 | 1.329 | Beta-glucan synthesis-associated protein KRE6 |
| A10948 | 681 | 1.461 | 2.066 | Cell division control protein 14, SIN component |
| A10980 | 492 | 1.325 | 1.809 | UDP-N-acetylglucosamine transferase subunit |
| A10989 | 888 | 1.236 | 1.843 | Nucleophosmin |
| A11018 | 798 | 1.998 | 3.366 | F-box domain-containing protein |
| A11028 | 483 | 2.695 | 3.799 | F-box domain-containing protein |
| A11056 | 828 | 1.398 | 2.234 | KOW motif |
| A11070 | 612 | 1.220 | 1.639 | Ribosome biogenesis protein SLX9 |
| A11071 | 567 | 2.190 | 4.385 | Chitin synthase export chaperone |
| A11087 | 2715 | 1.699 | 3.264 | Fusarisetin A cluster transcription factor fsa6 |
| A11092 | 615 | 1.499 | 2.616 | 60S ribosomal protein L1-B |
| A11111 | 279 | 1.801 | 1.356 | DNA recombination and repair protein Rad51-like C-terminal domain-containing protein |
| A11121 | 1878 | 1.424 | 2.305 | Ribosomal RNA-processing protein 7 (RRP7) C-terminal domain |
| A11127 | 1113 | 1.276 | 1.813 | Pre-rRNA-processing protein ESF2 |
| A11129 | 375 | 1.896 | 3.094 | Large ribosomal subunit protein uL23 |
| A11141 | 1350 | 1.557 | 2.747 | Glutaryl-CoA dehydrogenase, mitochondrial |
| A11171 | 1263 | 2.366 | 3.879 | Putative mannan endo-1,4-beta-mannosidase 5 |
| A11172 | 1671 | 2.856 | 8.398 | Sulfite oxidase, mitochondrial |
| A11175 | 216 | 1.276 | 1.460 | DUF4050 domain-containing protein |
| A11189 | 822 | 1.709 | 3.251 | Zinc knuckle |
| A11251 | 1113 | 4.163 | 15.686 | Probable diacetyl reductase |
| A11257 | 1653 | 2.235 | 5.291 | Ribosome assembly protein rrb1 |
| A11259 | 630 | 4.983 | 9.198 | Carboxypeptidase |
| A11267 | 822 | 3.870 | 13.803 | Short-chain dehydrogenase/reductase |
| A11298 | 624 | 2.460 | 5.091 | Putative effector protein |
| A11303 | 396 | 1.131 | 1.512 | Mitochondrial import inner membrane translocase |
| A11330 | 720 | 1.486 | 2.577 | 60S ribosomal protein L2 |
| A11343 | 1410 | 2.530 | 4.716 | Cupin type-2 domain-containing protein |
| A11373 | 1161 | 1.240 | 1.510 | F-box-like |
| A11377 | 1167 | 1.338 | 2.119 | F-box domain-containing protein |
| A11385 | 783 | 1.397 | 2.215 | Lipoyl synthase, mitochondrial |
| A11387 | 888 | 5.463 | 15.955 | F-box-like |
| A11391 | 2301 | 1.801 | 1.356 | Microtubule/TRAF3 and DISC1 binding protein |
| A11409 | 3540 | 2.508 | 6.341 | U3 small nucleolar RNA-associated protein 25 |
| A11428 | 504 | 1.159 | 1.642 | Translationally-controlled tumor protein homolog |
| A11432 | 831 | 3.618 | 12.640 | Protein alcS |
| A11433 | 1905 | 2.745 | 7.845 | Probable indole-3-pyruvate monooxygenase |
| A11434 | 1056 | 1.531 | 2.531 | Monocarboxylate transporter 12 |
| A11440 | 1641 | 1.425 | 2.334 | C2H2-type domain-containing protein |
| A11481 | 471 | 1.211 | 1.766 | 40S ribosomal protein S11-A |
| A11502 | 666 | 1.204 | 1.727 | PABS domain-containing protein |
| A11532 | 1860 | 1.872 | 3.482 | Scytalone dehydratase-like protein Arp1 |
| A11534 | 1068 | 2.389 | 5.385 | CaM kinase-like vesicle-associated protein |
| A11554 | 2586 | 2.599 | 6.842 | 20S-pre-rRNA D-site endonuclease nob1 |
| A11564 | 585 | 1.231 | 1.831 | 40S ribosomal protein S5 |
| A11567 | 495 | 1.450 | 2.397 | Lactoylglutathione lyase |
| A11569 | 2073 | 1.303 | 2.014 | Peroxisomal acyl-coenzyme A oxidase 1 |
| A11584 | 582 | 4.601 | 3.748 | Spindle assembly checkpoint component |
| A11585 | 1983 | 3.453 | 6.640 | Checkpoint serine/threonine-protein kinase |
| A11590 | 1416 | 1.481 | 2.423 | Manganese catalase family protein |
| A11597 | 1626 | 1.672 | 3.182 | NADP-dependent malic enzyme |
| A11599 | 723 | 1.125 | 1.366 | HIT-type domain-containing protein |
| A11605 | 753 | 1.356 | 2.026 | Pentacotripeptide-repeat region of PRORP domain-containing protein |
| A11626 | 966 | 2.445 | 2.297 | Glycosyl hydrolases family 16 |
| A11637 | 1989 | 1.333 | 1.973 | Streptomycin-6-phosphate phosphatase |
| A11648 | 1044 | 4.985 | 13.196 | Probable glycosidase C21B10.07 |
| A11657 | 1773 | 1.858 | 3.772 | Amino-acid permease |
| A11659 | 1554 | 2.452 | 6.072 | Amino-acid permease inda1 |
| A11660 | 291 | 2.551 | 5.117 | Amino acid permease/ SLC12A domain-containing protein |
| A11662 | 1740 | 1.138 | 1.498 | Cytochrome P450 monooxygenase 124 |
| A11676 | 867 | 1.205 | 1.694 | Peroxisomal biogenesis factor 11 (PEX11) |
| A11695 | 1524 | 1.110 | 1.494 | Mitochondrial import inner membrane translocase subunit TIM44 |
| A11705 | 1011 | 2.502 | 6.482 | Pyridoxal kinase |
| A11729 | 2373 | 2.551 | 5.992 | Probable beta-glucosidase G |
| A11749 | 1470 | 5.951 | 26.215 | MFS siderochrome iron transporter 1 |
| A11750 | 3348 | 1.183 | 1.710 | Sodium transport ATPase 2 |
| A11751 | 1716 | 4.439 | 17.088 | Vanillyl-alcohol oxidase |
| A11752 | 891 | 3.323 | 10.508 | alpha/beta hydrolase fold |
| A11757 | 1227 | 1.651 | 3.060 | Lovastatin esterase |
| A11762 | 1758 | 1.208 | 1.728 | High-affinity methionine permease |
| A11769 | 885 | 1.439 | 2.252 | Checkpoint protein HUS1 |
| A11774 | 675 | 1.449 | 1.520 | G-protein coupled receptors family 1 profile domain-containing protein |
| A11791 | 1779 | 2.785 | 6.040 | Serine/threonine-protein kinase ksp1 |
| A11794 | 639 | 1.782 | 2.519 | peptidylprolyl isomerase |
| A11811 | 927 | 1.162 | 1.554 | Peptidyl-prolyl cis-trans isomerase E |
| A11814 | 1683 | 1.994 | 3.529 | Periplasmic copper-binding protein NosD beta helix domain-containing protein |
| A11828 | 1407 | 3.063 | 4.274 | Uncharacterized MFS-type transporter |
| A11829 | 1779 | 3.330 | 6.369 | Fungal trichothecene efflux pump |
| A11831 | 1845 | 1.179 | 1.631 | Acetoacetyl-CoA synthetase |
| A11837 | 654 | 4.097 | 6.461 | N-acetyltransferase domain-containing protein |
| A11839 | 507 | 1.062 | 1.353 | Zinc finger protein 593 |
| A11849 | 762 | 1.188 | 1.489 | Major facilitator superfamily (MFS) profile domain-containing protein |
| A11852 | 804 | 2.369 | 3.755 | 2,5-dichloro-2,5-cyclohexadiene-1,4-diol dehydrogenase |
| A11853 | 1782 | 1.852 | 3.530 | Fungal trichothecene efflux pump (TRI12) |
| A11854 | 1758 | 1.974 | 4.159 | Arylsulfatase |
| A11855 | 1713 | 1.470 | 1.779 | Uncharacterized transporter YIL166C |
| A11865 | 1551 | 2.494 | 6.025 | Uncharacterized transporter YIL166C |
| A11877 | 1440 | 2.344 | 3.043 | Glutamate receptor ionotropic, NMDA 3A-like isoform X2 |
| A11926 | 540 | 1.204 | 1.454 | Pentatricopeptide repeat domain |
| A11934 | 450 | 8.736 | 7.925 | RING-type domain-containing protein |
| A11937 | 783 | 2.041 | 3.704 | Histone H1 |
| A11957 | 432 | 7.016 | 2.834 | Glucose dehydrogenase |
| A11993 | 1092 | 3.532 | 9.794 | Glycosyl hydrolase family 26 |
| A12007 | 2595 | 2.032 | 3.879 | F-box domain-containing protein |
| A12010 | 567 | 1.425 | 2.378 | Protein CPL1-like domain-containing protein |
| A12017 | 456 | 1.402 | 1.842 | Septum-promoting GTP-binding protein 1 |
| A12021 | 1671 | 2.178 | 4.882 | ATP-dependent RNA helicase ROK1 |
| A12034 | 408 | 4.304 | 14.414 | Histone H3.2 |
| A12037 | 750 | 2.013 | 3.758 | Ubiquitin-like domain-containing protein |
| A12043 | 1008 | 2.438 | 3.829 | F-box domain-containing protein |
| A12107 | 495 | 1.589 | 2.542 | Apple domain-containing protein |
| A12120 | 711 | 3.265 | 5.599 | Mitotic spindle checkpoint component mad2 |
| A12121 | 549 | 2.137 | 1.839 | zinc-RING finger domain |
| A12150 | 717 | 2.546 | 5.668 | Phosphatidylinositol-specific phospholipase |
| A12181 | 1209 | 1.777 | 3.334 | RNA polymerase II transcription factor SIII |
| A12186 | 1131 | 3.218 | 3.487 | GPI-anchored protein |
| A12216 | 1386 | 2.462 | 6.186 | Ribosome biogenesis protein YTM1 |
| A12237 | 2097 | 1.335 | 2.051 | Ubiquitin-like modifier-activating enzyme |
| A12238 | 1068 | 2.364 | 5.972 | GPI anchored dioxygenase |
| A12259 | 2031 | 3.468 | 9.190 | Nucleolar and coiled-body phosphoprotein 1 isoform X9 |
| A12268 | 1842 | 2.339 | 5.447 | UDP-glucuronic acid decarboxylase 1 |
| A12284 | 1788 | 1.288 | 1.884 | L-arabinokinase |
| A12287 | 612 | 3.655 | 12.012 | Cysteine-rich secretory protein family |
| A12288 | 684 | 1.452 | 2.474 | Cysteine-rich secretory protein family |
| A12300 | 687 | 2.934 | 1.657 | N-acetyltransferase domain-containing protein |
| A12325 | 987 | 2.202 | 4.804 | 25S rRNA (uridine(2843)-N(3))-methyltransferase |
| A12346 | 1509 | 1.590 | 2.805 | DNA-directed RNA polymerase I subunit |
| A12352 | 399 | 1.282 | 1.643 | Thioesterase domain-containing protein |
| A12353 | 1668 | 2.122 | 4.613 | Glycosyl hydrolases family 18 |
| A12354 | 387 | 1.430 | 2.393 | 60S ribosomal protein L22 |
| A12358 | 579 | 1.486 | 1.755 | DUF6533 domain-containing protein |
| A12383 | 1170 | 1.329 | 1.517 | Rad51 |
| A12393 | 999 | 1.597 | 2.767 | Dimethyladenosine transferase |
| A12400 | 1311 | 1.776 | 3.133 | Probable glucan endo-1,3-beta-glucosidase |
| A12438 | 438 | 3.782 | 13.554 | Lytic transglycolase |
| A12449 | 639 | 1.884 | 1.711 | Receptor-like serine/threonine-protein kinase |
| A12483 | 486 | 1.305 | 2.032 | 40S ribosomal protein S10-B |
| A12485 | 1092 | 2.100 | 4.466 | Galactosyltransferase |
| A12489 | 957 | 1.133 | 1.381 | Acyl-coenzyme A thioesterase 8 |
| A12496 | 1680 | 1.687 | 2.283 | MFS-type transporter oryN |
| A12500 | 1131 | 2.057 | 4.190 | U3-containing 90S pre-ribosomal complex |
| A12506 | 1953 | 1.157 | 1.642 | Acyltransferase family |
| A12508 | 1437 | 1.452 | 2.472 | Ferric reductase NAD binding domain |
| A12521 | 345 | 1.615 | 2.468 | Core histone H2A/H2B/H3/H4 |
| A12544 | 609 | 2.128 | 4.492 | Translation machinery-associated protein 16 |
| A12586 | 378 | 3.893 | 7.514 | Uncharacterized conserved protein (DUF2340) |

**Table S4**. The protein sequence identity score of SiCut1, SiCut2 and other cutinases. Identity scores was calculated using Multiple Sequence Alignment by CLUSTALW (https://www.genome.jp/tools-bin/clustalw). The accession numbers of these enzymes have been indicated in Figure 2.

| **Scores** | **SiCut1** | **SiCut2** | **CmCut1** | **PlCut1** | **CLE** | **PaE** | **BaCut1** | **Acut1p** | **Acut2p** | **Acut3p** | **Cr14CLE** | **McCut** | **AoCut** | **FsCut1** | **FoCut** | **CgCut** | **HiCut** | **PCLE** | **RgCut** | **AdCut** | **PsCut** | **MtCut** | **SvCut** | **TcCut** | **TaCut** | **LCC** | **PETase** | **TfCut** | Ref. |
| --- | --- | --- | --- | --- | --- | --- | --- | --- | --- | --- | --- | --- | --- | --- | --- | --- | --- | --- | --- | --- | --- | --- | --- | --- | --- | --- | --- | --- | --- |
| **SiCut1** | 100 | 74.20 | 17.30 | 16.20 | 20.70 | 17.90 | 18.40 | 18.99 | 17.32 | 14.53 | 16.76 | 17.30 | 15.60 | 17.90 | 19.60 | 16.80 | 19.60 | 17.30 | 15.10 | 14.50 | 13.40 | 13.40 | 14.00 | 12.30 | 13.40 | 13.41 | 15.08 | 13.41 | This |
| **SiCut2** | 74.20 | 100 | 19.70 | 16.90 | 19.10 | 19.10 | 19.10 | 19.10 | 17.98 | 16.85 | 16.85 | 18.00 | 16.90 | 18.50 | 18.50 | 18.50 | 19.70 | 20.20 | 13.50 | 15.70 | 12.40 | 13.50 | 14.00 | 11.80 | 12.40 | 13.48 | 13.48 | 13.48 | This |
| **CmCut1** | 17.30 | 19.70 | 100 | 62.80 | 59.70 | 54.90 | 60.80 | 57.59 | 59.01 | 59.29 | 62.83 | 19.50 | 16.00 | 15.90 | 16.80 | 17.40 | 17.50 | 15.50 | 13.30 | 12.40 | 13.30 | 13.30 | 14.60 | 12.40 | 13.70 | 15.49 | 12.39 | 12.39 | [1] |
| **PlCut1** | 16.20 | 16.90 | 62.80 | 100 | 59.90 | 59.40 | 53.60 | 55.36 | 52.25 | 50.63 | 77.06 | 19.10 | 17.40 | 15.20 | 16.50 | 15.60 | 18.00 | 18.10 | 12.20 | 12.70 | 14.80 | 14.80 | 13.50 | 11.40 | 14.30 | 14.77 | 12.24 | 13.92 | [2] |
| **CLE** | 20.70 | 19.10 | 59.70 | 59.90 | 100 | 57.10 | 51.80 | 50.45 | 50.45 | 50.21 | 62.34 | 15.30 | 15.50 | 17.00 | 15.20 | 17.90 | 18.60 | 16.30 | 13.40 | 13.00 | 14.20 | 13.40 | 14.60 | 11.30 | 13.40 | 14.23 | 14.64 | 12.97 | [3] |
| **PaE** | 17.90 | 19.10 | 54.90 | 59.40 | 57.10 | 100 | 54.10 | 55.80 | 55.41 | 58.48 | 60.27 | 18.10 | 15.00 | 15.20 | 15.20 | 17.00 | 19.10 | 15.20 | 13.80 | 12.90 | 15.20 | 12.90 | 13.80 | 12.10 | 13.40 | 14.73 | 11.16 | 12.05 | [4] |
| **BaCut1** | 18.40 | 19.10 | 60.80 | 53.60 | 51.80 | 54.10 | 100 | 83.33 | 89.64 | 73.87 | 53.60 | 16.70 | 15.50 | 14.90 | 16.20 | 16.70 | 19.60 | 12.60 | 12.20 | 13.50 | 12.60 | 14.40 | 12.20 | 12.20 | 12.60 | 13.96 | 11.71 | 13.51 | [5] |
| **Acut1p** | 18.99 | 19.10 | 57.59 | 55.36 | 50.45 | 55.80 | 83.33 | 100 | 88.74 | 78.13 | 54.02 | 20.00 | 15.96 | 12.50 | 16.07 | 18.30 | 21.65 | 12.50 | 12.50 | 12.05 | 11.61 | 13.39 | 11.16 | 12.05 | 12.05 | 14.29 | 11.61 | 12.50 | [6] |
| **Acut2p** | 17.32 | 17.98 | 59.01 | 52.25 | 50.45 | 55.41 | 89.64 | 88.74 | 100 | 77.93 | 52.25 | 18.60 | 16.43 | 12.61 | 17.57 | 17.12 | 20.62 | 12.61 | 11.71 | 13.06 | 12.16 | 14.86 | 13.06 | 12.61 | 13.06 | 13.06 | 11.71 | 13.51 | [6] |
| **Acut3p** | 14.53 | 16.85 | 59.29 | 50.63 | 50.21 | 58.48 | 73.87 | 78.13 | 77.93 | 100 | 54.11 | 18.60 | 15.96 | 16.96 | 15.65 | 15.18 | 17.01 | 14.98 | 11.47 | 10.86 | 11.92 | 11.25 | 9.87 | 11.45 | 10.67 | 11.26 | 14.14 | 12.29 | [6] |
| **Cr14CLE** | 16.76 | 16.85 | 62.83 | 77.06 | 62.34 | 60.27 | 53.60 | 54.02 | 52.25 | 54.11 | 100 | 15.81 | 17.37 | 16.96 | 17.83 | 18.30 | 18.56 | 18.06 | 13.85 | 14.29 | 13.42 | 13.42 | 12.55 | 12.55 | 13.85 | 14.72 | 13.85 | 12.99 | [7] |
| **McCut** | 17.30 | 18.00 | 19.50 | 19.10 | 15.30 | 18.10 | 16.70 | 20.00 | 18.60 | 18.60 | 15.81 | 100 | 56.30 | 40.90 | 36.70 | 45.60 | 49.00 | 42.30 | 12.60 | 11.60 | 13.00 | 12.60 | 11.60 | 11.60 | 13.50 | 12.09 | 13.49 | 11.63 | [8] |
| **AoCut** | 15.60 | 16.90 | 16.00 | 17.40 | 15.50 | 15.00 | 15.50 | 15.96 | 16.43 | 15.96 | 17.37 | 56.30 | 100 | 43.70 | 43.70 | 46.50 | 43.30 | 43.70 | 15.50 | 12.20 | 13.60 | 13.10 | 13.60 | 11.70 | 14.10 | 14.55 | 13.62 | 13.15 | [9] |
| **FsCut1** | 17.90 | 18.50 | 15.90 | 15.20 | 17.00 | 15.20 | 14.90 | 12.50 | 12.61 | 16.96 | 16.96 | 40.90 | 43.70 | 100 | 75.70 | 45.10 | 51.50 | 53.70 | 13.50 | 14.30 | 13.50 | 13.50 | 13.50 | 12.20 | 13.00 | 13.91 | 12.17 | 13.48 | [10] |
| **FoCut** | 19.60 | 18.50 | 16.80 | 16.50 | 15.20 | 15.20 | 16.20 | 16.07 | 17.57 | 15.65 | 17.83 | 36.70 | 43.70 | 75.70 | 100 | 46.40 | 50.50 | 53.30 | 14.30 | 13.90 | 14.80 | 13.50 | 13.90 | 10.90 | 13.90 | 13.04 | 14.78 | 13.91 | [11] |
| **CgCut** | 16.80 | 18.50 | 17.40 | 15.60 | 17.90 | 17.00 | 16.70 | 18.30 | 17.12 | 15.18 | 18.30 | 45.60 | 46.50 | 45.10 | 46.40 | 100 | 55.70 | 50.90 | 14.70 | 12.50 | 12.50 | 12.90 | 14.70 | 12.50 | 15.60 | 12.05 | 12.50 | 14.29 | [12] |
| **HiCut** | 19.60 | 19.70 | 17.50 | 18.00 | 18.60 | 19.10 | 19.60 | 21.65 | 20.62 | 17.01 | 18.56 | 49.00 | 43.30 | 51.50 | 50.50 | 55.70 | 100 | 59.30 | 13.40 | 12.90 | 11.30 | 10.80 | 11.30 | 11.90 | 10.80 | 13.40 | 13.40 | 12.37 | [13] |
| **PCLE** | 17.30 | 20.20 | 15.50 | 18.10 | 16.30 | 15.20 | 12.60 | 12.50 | 12.61 | 14.98 | 18.06 | 42.30 | 43.70 | 53.70 | 53.30 | 50.90 | 59.30 | 100 | 13.20 | 12.30 | 13.70 | 15.00 | 14.50 | 12.30 | 13.20 | 13.66 | 14.54 | 13.22 | [14] |
| **RgCut** | 15.10 | 13.50 | 13.30 | 12.20 | 13.40 | 13.80 | 12.20 | 12.50 | 11.71 | 11.47 | 13.85 | 12.60 | 15.50 | 13.50 | 14.30 | 14.70 | 13.40 | 13.20 | 100 | 58.80 | 40.50 | 38.40 | 37.60 | 43.90 | 41.60 | 37.28 | 59.86 | 42.29 | [15] |
| **AdCut** | 14.50 | 15.70 | 12.40 | 12.70 | 13.00 | 12.90 | 13.50 | 12.05 | 13.06 | 10.86 | 14.29 | 11.60 | 12.20 | 14.30 | 13.90 | 12.50 | 12.90 | 12.30 | 58.80 | 100 | 39.70 | 38.50 | 36.80 | 44.70 | 41.30 | 39.59 | 76.21 | 40.53 | [16] |
| **PsCut** | 13.40 | 12.40 | 13.30 | 14.80 | 14.20 | 15.20 | 12.60 | 11.61 | 12.16 | 11.92 | 13.42 | 13.00 | 13.60 | 13.50 | 14.80 | 12.50 | 11.30 | 13.70 | 40.50 | 39.70 | 100 | 37.10 | 39.10 | 45.00 | 40.00 | 39.93 | 44.48 | 40.86 | [17] |
| **MtCut** | 13.40 | 13.50 | 13.30 | 14.80 | 13.40 | 12.90 | 14.40 | 13.39 | 14.86 | 11.25 | 13.42 | 12.60 | 13.10 | 13.50 | 13.50 | 12.90 | 10.80 | 15.00 | 38.40 | 38.50 | 37.10 | 100 | 58.90 | 57.60 | 54.70 | 46.76 | 38.28 | 53.49 | [18] |
| **SvCut** | 14.00 | 14.00 | 14.60 | 13.50 | 14.60 | 13.80 | 12.20 | 11.16 | 13.06 | 9.87 | 12.55 | 11.60 | 13.60 | 13.50 | 13.90 | 14.70 | 11.30 | 14.50 | 37.60 | 36.80 | 39.10 | 58.90 | 100 | 61.80 | 54.70 | 46.08 | 38.28 | 56.15 | [19] |
| **TcCut** | 12.30 | 11.80 | 12.40 | 11.40 | 11.30 | 12.10 | 12.20 | 12.05 | 12.61 | 11.45 | 12.55 | 11.60 | 11.70 | 12.20 | 10.90 | 12.50 | 11.90 | 12.30 | 43.90 | 44.70 | 45.00 | 57.60 | 61.80 | 100 | 82.10 | 53.05 | 45.42 | 99.62 | [20] |
| **TaCut** | 13.40 | 12.40 | 13.70 | 14.30 | 13.40 | 13.40 | 12.60 | 12.05 | 13.06 | 10.67 | 13.85 | 13.50 | 14.10 | 13.00 | 13.90 | 15.60 | 10.80 | 13.20 | 41.60 | 41.30 | 40.00 | 54.70 | 54.70 | 82.10 | 100 | 50.85 | 41.72 | 80.33 | [21] |
| **LCC** | 13.41 | 13.48 | 15.49 | 14.77 | 14.23 | 14.73 | 13.96 | 14.29 | 13.06 | 11.26 | 14.72 | 12.09 | 14.55 | 13.91 | 13.04 | 12.05 | 13.40 | 13.66 | 37.28 | 39.59 | 39.93 | 46.76 | 46.08 | 53.05 | 50.85 | 100 | 39.31 | 49.49 | [22] |
| **PETase** | 15.08 | 13.48 | 12.39 | 12.24 | 14.64 | 11.16 | 11.71 | 11.61 | 11.71 | 14.14 | 13.85 | 13.49 | 13.62 | 12.17 | 14.78 | 12.50 | 13.40 | 14.54 | 59.86 | 76.21 | 44.48 | 38.28 | 38.28 | 45.42 | 41.72 | 39.31 | 100 | 43.45 | [23] |
| **TfCut** | 13.41 | 13.48 | 12.39 | 13.92 | 12.97 | 12.05 | 13.51 | 12.50 | 13.51 | 12.29 | 12.99 | 11.63 | 13.15 | 13.48 | 13.91 | 14.29 | 12.37 | 13.22 | 42.29 | 40.53 | 40.86 | 53.49 | 56.15 | 99.62 | 80.33 | 49.49 | 43.45 | 100 | [24] |

**References:**

[1] Ueda, H., Tabata, J., Seshime, Y., Masaki, K., Sameshima-Yamashita, Y., Kitamoto, H., 2021. Cutinase-like biodegradable plastic-degrading enzymes from phylloplane yeasts have cutinase activity. Biosci. Biotechnol. Biochem. 85, 1890-1898. <https://doi.org/10.1093/bbb/zbab113>.

[2] Roman, V.A., Crable, B.R., Wagner, D.N., Gryganskyi, A., Zelik, S., Cummings, L., Hung, C.S., Nadeau, L.J., Schratz, L., Haridas, S., Pangilinan, J., Lipzen, A., Na, H., Yan, M., Ng, V., Grigoriev, I.V., Barlow, D., Biffinger, J., Kelley-Loughnane, N., Crookes-Goodson, W.J., Stamps, B., Varaljay, V.A., 2024. Identification and recombinant expression of a cutinase from *Papiliotrema laurentii* that hydrolyzes natural and synthetic polyesters. Appl. Environ. Microbiol. 90, <https://doi.org/10.1128/aem.01694-23>.

[3] Masaki, K., Kamini, N.R., Ikeda, H., Iefuji, H., 2005. Cutinase-like enzyme from the yeast *Cryptococcus* sp. strain S-2 hydrolyzes polylactic acid and other biodegradable plastics. Appl. Environ. Microbiol. 71, 7548-7550. <https://doi.org/10.1128/AEM.71.11.7548-7550.2005>.

[4] Shinozaki, Y., Morita, T., Cao, X.H., Yoshida, S., Koitabashi, M., Watanabe, T., Suzuki, K., Sameshima-Yamashita, Y., Nakajima-Kambe, T., Fujii, T., Kitamoto, H.K., 2013. Biodegradable plastic-degrading enzyme from *Pseudozyma antarctica*: Cloning, sequencing, and characterization. Appl. Microbiol. Biotechnol. 97, 2951-2959. <https://doi.org/10.1007/s00253-012-4188-8>.

[5] Jiang, Z., Chen, X., Xue, H., Li, Z., Lei, J., Yu, M., Yan, X., Cao, H., Zhou, J., Liu, J., Zheng, M., Dong, W., Li, Y., Cui, Z., 2024. Novel polyurethane-degrading cutinase BaCut1 from *Blastobotrys* sp. G-9 with potential role in plastic bio-recycling. J. Hazard. Mater. 472, 134493. <https://doi.org/10.1016/j.jhazmat.2024.134493>.

[6] Bischoff, F., K. Litwinska, A. Cordes, K. Baronian, R. Bode, F. Schauer, and G. Kunze. 2015. Three new cutinases from the yeast *Arxula adeninivorans* that are suitable for biotechnological applications. Appl. Environ. Microbiol. 81:5497-5510.

[7] Arunrattanamook, N., Mhuantong, W., Paemanee, A., Reamtong, O., Hararak, B., & Champreda, V. (2023). Identification of a plastic-degrading enzyme from Cryptococcus nemorosus and its use in self-degradable plastics. Appl. Microbiol. Biotechnol. [https://doi.org/10.1007/s00253-023-12816-6](https://doi.org/10.1007/s00253-023-12816-6)

[8] Duan, X., Liu, Y., You, X., Jiang, Z., Yang, S., Yang, S., 2017. High-level expression and characterization of a novel cutinase from *Malbranchea cinnamomea* suitable for butyl butyrate production. Biotechnol. Biofuels 10, 223. <https://doi.org/10.1186/s13068-017-0912-z>.

[9] Maeda, H., Yamagata, Y., Abe, K., Hasegawa, F., Machida, M., Ishioka, R., Gomi, K., Nakajima, T., 2005. Purification and characterization of a biodegradable plastic-degrading enzyme from *Aspergillus oryzae*. Appl. Microbiol. Biotechnol. 67, 778-788. <https://doi.org/10.1007/s00253-004-1853-6>.

[10] Egmond, M.R., de Vlieg, J., 2000. *Fusarium solani pisi* cutinase. Biochimie 82, 1015−1021. <https://doi.org/10.1016/S0300-9084(00)01183-4>.

[11] Dimarogona, M., Nikolaivits, E., Kanelli, M., Christakopoulos, P., Sandgren, M., Topakas, E., 2015. Structural and functional studies of a *Fusarium oxysporum* cutinase with polyethylene terephthalate modification potential. Biochim. Biophys. Acta. 1850, 2308-2317. <https://doi.org/10.1016/j.bbagen.2015.08.009>.

[12] Chen, Z., Franco, C.F., Baptista, R.P., Cabral, J.M., Coelho, A.V., Rodrigues, C.J., Jr., Melo, E.P., 2007. Purification and identification of cutinases from *Colletotrichum kahawae* and *Colletotrichum gloeosporioides*. Appl. Microbiol. Biotechnol. 73, 1306-1313. <https://doi.org/10.1007/s00253-006-0605-1>.

[13] Kold, D., Dauter, Z., Laustsen, A.K., Brzozowski, A.M., Turkenburg, J.P., Nielsen, A.D., Koldso, H., Petersen, E., Schiott, B., De Maria, L., Wilson, K.S., Svendsen, A., Wimmer, R., 2014. Thermodynamic and structural investigation of the specific SDS binding of *Humicola insolens* cutinase. Protein Sci. 23, 1023-1035. <https://doi.org/10.1002/pro.2489>.

[14] Suzuki, K., Noguchi, M.T., Shinozaki, Y., Koitabashi, M., Sameshima-Yamashita, Y., Yoshida, S., Fujii, T., Kitamoto, H.K., 2014. Purification, characterization, and cloning of the gene for a biodegradable plastic-degrading enzyme from *Paraphoma*-related fungal strain B47-9. Appl. Microbiol. Biotechnol. 98, 4457-4465. 10.1007/s00253-013-5454-0.

[15] Sagong, H.Y., Son, H.F., Seo, H., Hong, H., Lee, D., Kim, K.J., 2021. Implications for the PET decomposition mechanism through similarity and dissimilarity between PETases from *Rhizobacter gummiphilus* and *Ideonella sakaiensis*. J. Hazard. Mater. 416, 126075. <https://doi.org/10.1016/j.jhazmat.2021.126075>.

[16] Uchida, H., Shigeno-Akutsu, Y., Nomura, N., Nakahara, T., Nakajima-Kambe, T., 2002. Cloning and sequence analysis of poly(tetramethylene succinate) depolymerase from *Acidovorax delafieldii* strain BS-3. J. Biosci. Bioeng. 93, 245-247. <https://doi.org/10.1016/S1389-1723(02)80022-6>.

[17] Avilan, L., Lichtenstein, B.R., Konig, G., Zahn, M., Allen, M.D., Oliveira, L., Clark, M., Bemmer, V., Graham, R., Austin, H.P., Dominick, G., Johnson, C.W., Beckham, G.T., McGeehan, J.E., Pickford, A.R., 2023. Concentration-dependent inhibition of mesophilic PETases on poly(ethylene terephthalate) can be eliminated by enzyme engineering. ChemSusChem 16, e202202277. <https://doi.org/10.1002/cssc.202202277>.

[18] Liu, Y., Liu, C., Liu, H., Zeng, Q., Tian, X., Long, L., Yang, J., 2022. Catalytic features and thermal adaptation mechanisms of a deep sea bacterial cutinase-type poly(ethylene terephthalate) hydrolase. Front. Bioeng. Biotechnol. 10, 865787. <https://doi.org/10.3389/fbioe.2022.865787>.

[19] Kawai, F., Oda, M., Tamashiro, T., Waku, T., Tanaka, N., Yamamoto, M., Mizushima, H., Miyakawa, T., Tanokura, M., 2014. A novel Ca^2+^-activated, thermostabilized polyesterase capable of hydrolyzing polyethylene terephthalate from *Saccharomonospora viridis* AHK190. Appl. Microbiol. Biotechnol. 98, 10053-10064. <https://doi.org/10.1007/s00253-014-5860-y>.

[20] Herrero Acero, E., Ribitsch, D., Dellacher, A., Zitzenbacher, S., Marold, A., Steinkellner, G., Gruber, K., Schwab, H., Guebitz, G.M., 2013. Surface engineering of a cutinase from *Thermobifida cellulosilytica* for improved polyester hydrolysis. Biotechnol. Bioeng. 110, 2581-2590. <https://doi.org/10.1002/bit.24930>.

[21] Ribitsch, D., Acero, E.H., Greimel, K., Eiteljoerg, I., Trotscha, E., Freddi, G., Schwab, H., Guebitz, G.M., 2011. Characterization of a new cutinase from *Thermobifida albafor* PET-surface hydrolysis. Biocatal. Biotransform. 30, 2-9. <https://doi.org/10.3109/10242422.2012.644435>.

[22] Sulaiman, S., Yamato, S., Kanaya, E., Kim, J.J., Koga, Y., Takano, K., Kanaya, S., 2012. Isolation of a novel cutinase homolog with polyethylene terephthalate-degrading activity from leaf-branch compost by using a metagenomic approach. Appl. Environ. Microbiol. 78, 1556-1562. <https://doi.org/10.1128/AEM.06725-11>.

[23] Yoshida, S., Hiraga, K., Takehana, T., Taniguchi, I., Yamaji, H., Maeda, Y., Toyohara, K., Miyamoto, K., Kimura, Y., & Oda, K. 2016. A bacterium that degrades and assimilates poly(ethylene terephthalate). Science 351, 1196-1199. https://doi.org/10.1126/science.aad6359.

[24] Chen, S., Tong, X., Woodard, R.W., Du, G., Wu, J., Chen, J., 2008. Identification and characterization of bacterial cutinase. J. Biol. Chem. 283, 25854-25862. https://doi.org/10.1074/jbc.M800848200.
